# Interpenetrating Dual Epoxy Networks with Reversible Covalent Bonds: Shape-Constant Self-Healing Electrically Insulating Materials

**DOI:** 10.1021/acsapm.6c01094

**Published:** 2026-08-20

**Authors:** Beata Strachota, Adam Strachota, Petr Kadlec, Radek Polanský, Todor Batakliev

**Affiliations:** † Institute of Macromolecular Chemistry, 86879 Czech Academy of Sciences, Heyrovského nám. 2, Praha 16200, Czech Republic; ‡ Department of Materials and Technology, Faculty of Electrical Engineering, 48232 University of West Bohemia, Univerzitní 2795/26, Plzeň 30100, Czech Republic; § Institute of Mechanics, 234882 Bulgarian Academy of Sciences, Acad. G. Bonchev Str. Block 4, Sofia 1113, Bulgaria; ∥ Center of Competence for Mechatronics and Clean Technologies “Mechatronics, Innovation, Robotics, Automation and Clean Technologies” - MIRACle, Sofia 1113, Bulgaria

**Keywords:** self-healing polymer, Diels−Alder chemistry, electrical insulating material, dielectric spectroscopy, mechanical properties, epoxy resin, thermal analysis

## Abstract

Novel dual interpenetrating epoxy networks (IPNs) with highly promising self-healing and electroinsulating properties were synthesized and systematically optimized. These systems display healing of microcracks and electrical tree damage. Their structure consists of a permanent epoxy-amine subnetwork (EP) interpenetrated with a reversible epoxy network (DA), the latter being cross-linked via Diels–Alder chemistry, through the reaction of maleimide and furan groups. The DA subnetwork provides excellent thermally triggered self-healing, while the permanent EP network ensures shape stability throughout the healing process. Although damage to the permanent EP subnetwork cannot be reversed, the mechanical and tensile properties of disrupted specimens are almost completely recovered after thermal healing. Both subnetworks incorporate flexible linear poly­(propylene oxide) (PPO) segments of different molecular weights (230, 400, and 2000 g/mol). Increasing the PPO chain length significantly reduces the glass transition temperature and the rubbery modulus, while also slightly decreasing the dissociation temperature of the reversible DA subnetwork. Synthesis and healing conditions were carefully designed to suppress undesired internetwork cross-linking side reactions, thereby preserving the reversibility of the DA bonds over repeated healing cycles. The effectiveness of this strategy was confirmed by cyclic thermal analyses and tensile tests conducted on promising healed specimens. Dielectric analysis was performed to assess the suitability of the developed IPNs for use in electrical insulation applications. The highest self-healing efficiency was achieved for the IPN containing the longest PPO chains, whereas the best dielectric insulating performance was observed for the shortest PPO chains. The IPN containing PPO chains with a molecular weight of 400 g/mol provided the best balance between self-healing capability and electroinsulating performance, making it the most promising candidate for self-healing electrical insulation applications.

## Graphical abstract

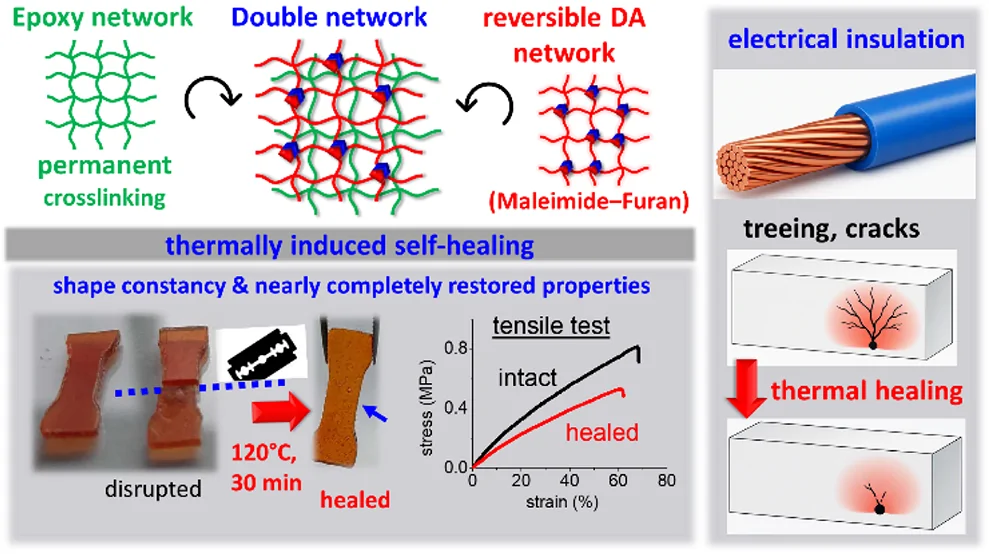

## 1 Introduction

This work focuses on the development of novel epoxy resins with self-healing ability, based on interpenetrating permanent and reversible polymer subnetworks, with particular emphasis on their potential application as electrical insulators. Growing research interest in such self-healing polymeric insulating materials is being driven by the increasing demand for durability and repairability in high-voltage applications.^1–5^ The ultimate goal is to improve the lifetime, reliability, and safety of modern electrical devices. With their ongoing miniaturization and the continuous increase in power density, the need for advanced insulating materials has become increasingly important. Polymers with high thermal conductivity and a low dielectric constant are of particular interest, as they enable the safe use of thinner insulating layers while maintaining the original voltage.^6,7^ Additional priorities include improved resistance to elevated operating temperatures (associated with increasingly demanding heat dissipation) and improved resistance to degradation processes (thereby increasing the reliability). In many cases, meeting these performance requirements is challenging, resulting in engineering trade-offs between optimal short-term functionality and the long-term product lifetime.^8^

Polymers, both thermoplastics (such as polyvinyl chloride and polyethylene) and thermosets (such as phenolic and epoxy resins), possess excellent electrical insulating properties.^9^ Among these materials, thermosets, and particularly epoxies and polyurethanes, are the dominant class of insulating materials used in electrical machines.^10–12^ Their principal limitations include brittleness and susceptibility to microcracking, which may result from prolonged vibration in rotating parts of electrical machines. Insulating materials used in electrical equipment are exposed not only to mechanical damage but also to electrical degradation. One of the primary forms of such damage is the formation of electrical trees—three-dimensional, dendritic, micrometer-sized channels that develop inside the material under the influence of a strong electric field.^13^ These structures are typically permanent and difficult to reverse. To address this challenge, self-healing polymeric materials have been investigated, as they should be able to restore structural integrity and recover the original material properties.^14–16^

Self-healing polymers (or polymer composites)^17^ belong to smart materials and can be classified according to both their structures and healing mechanisms. Self-healing thermosets may additionally offer attractive properties such as reprocessability and recyclability, which are not available in conventional thermosetting materials. True self-healing should occur without melting the material, whose original shape should stay preserved.^18^

Mechanisms of self-healing^19–21^ in polymers can be divided into two fundamental groups: extrinsic, based on microencapsulated healing agents released upon crack formation,^22^ and intrinsic, arising from the inherent properties of the polymer itself, as reviewed in ref 19 The intrinsic mechanism is based on reversible cross-linking, which governs the internal cohesion of the material,^20^ and, in principle, allows repeated healing cycles without significant deterioration of material performance. This type of self-healing may occur autonomously (at ambient or service temperature) or can be triggered by external stimuli, such as temperature, pressure, or light. Intrinsically self-healing polymers offer an important practical advantage of relatively straightforward synthesis. For glassy polymers, self-healing typically requires elevated temperatures to activate segmental mobility, either by exceeding the glass transition temperature or by reaching the onset temperature of bond-exchange reactions in adaptable networks, such as vitrimers.^23^

Chemical (reversible covalent bonding) or physical (molecular interdiffusion, partial melting, or physical cross-linking) interactions underlie both the extrinsic and the intrinsic self-healing mechanisms. However, systems based on reversible covalent chemistry have attracted the greatest research interest. The most extensively studied reversible covalent cross-linking mechanisms include disulfide exchange,^24^ imine exchange,^25^ ester exchange,^26,27^ and most importantly, the thermoreversible Diels–Alder (DA/retro-DA) cycloaddition between a diene and a dienophile (typically an activated monoolefin).^19,28^ Polyfunctional furan and maleimide derivatives are the most commonly used macro-monomers for DA-based systems.

Epoxy resins with self-healing capability have been reported previously, e.g., in refs 29–32 and 33. These systems exploit reversible DA/retro-DA bonding in the backbone of their epoxide and amine components, and this DA bonding typically is based on maleimide and furan groups.

Incorporation of reactive DA-polyfunctionalized graphene nanoplatelets has been shown to produce highly promising self-healing epoxy resins based on the DA/retro-DA mechanism.^34,35^ In ref 34, nanocomposite resins with significantly improved self-healing efficiency and mechanical properties were developed. In ref 35, the same concept was successfully extended to obtain highly attractive carbon-fiber-reinforced composites, which exhibited excellent mechanical performance together with repeatable self-healing capability.

Nanofiller-free special polymer architectures provide an alternative strategy for developing self-healing polymer networks:

Continuous permanent cross-linking, combined with an additional reversible one within the same polymer network backbone (“hybrid cross-linking”), represents one of two straightforward architectural approaches capable of combining (imperfect) intrinsic self-healing with shape stability.^18,36^ ^.^

Interpenetrating polymer networks (IPNs), also referred to as double networks, represent a second, highly promising architecture capable of achieving the same combination of properties.^37–39^ Moreover, IPNs are generally easier to synthesize than the “hybrid-cross-linked” systems.

Both the above architectures offer considerable potential for self-healing insulating coatings that retain their shape during healing. Nevertheless, complete recovery of the mechanical properties cannot be achieved, because damage to the permanent cross-links (e.g., epoxy–amine bonds) is irreversible.

In previous studies,^36–39^ the authors investigated several families of self-healing epoxy systems based on diglycidyl ether of bisphenol A (DGEBA) and poly­(propylene oxide) α,ω-diamine (D) as the main components. These systems were either fully or partly modified with DA functional groups (furan, maleimide), or they contained an intercalated DA subnetwork, thereby exploring both of the aforementioned architectural concepts. Several fully DA-based monomers were also evaluated. IPNs composed of DGEBA plus D (permanent network) and furan-modified D plus diphenylmethane bismaleimide (reversible network) were identified as particularly promising.^37,39^

The present work focuses on the development of a new electrical insulating material, intended for use as a thick insulating layer or protective coating, capable of self-healing of microcracks (electrical trees) generated during long-term exposure to high electric fields.

To this end, interpenetrating polymer networks (IPNs), consisting of a permanent epoxy–amine (EP) subnetwork interpenetrated with a reversible DA subnetwork, were prepared. The mesh size of both networks was systematically varied, building on the systems previously studied in ref 37. The synthesis and healing conditions were carefully designed to suppress undesired internetwork cross-linking side reactions, while enabling reproducible DA bond dissociation and recombination, thereby ensuring repeatable thermally triggered intrinsic self-healing.

The dielectric properties were optimized to achieve dielectric losses comparable to those of conventional DGEBA–diamine epoxy electrical insulators. The influence of the network mesh size, governed by the length and mobility of the partially polar elastic chains, on the dielectric behavior of the studied IPN systems was investigated by broadband dielectric spectroscopy (BDS). Particular emphasis was placed on the frequency and temperature dependence of the dielectric constant and the dielectric dissipation factor.

This work presents a new strategy for the design of self-healing insulating layers based on a double-network (IPN) architecture and DA/retro-DA chemistry. The topic of self-healing insulating polymers is comprehensively reviewed in ref 1, primarily with respect to thermoplastics used as cable insulation, rather than thermosets, which are the focus of the present study. For epoxy systems, the suppression of electrical tree formation via self-healing has previously been discussed in ref 2. However, that study focused primarily on the inorganic SiO_2_ filler, and the investigated epoxy matrix relied on a different self-healing mechanism. In contrast, the present work relies exclusively on thermoreversible Diels–Alder chemistry within the epoxy matrix itself, as the sole self-healing strategy, without the use of any fillers.

## 2 Experimental Section

### 2.1 Materials

The studied self-healing epoxy materials (IPNs) consisted of two interpenetrating polymer networks. The permanent epoxy–amine subnetwork (EP) was formed by curing a diepoxide with a diamine hardener. The second subnetwork (DA) was a reversible network formed through Diels–Alder chemistry. It comprised a commercial bifunctional dienophile and a tetrafunctional diene synthesized from commercially available precursors.

#### 2.1.1 Monomers for the Synthesis of Polymer Networks

The diepoxide monomer, diglycidyl ether of bisphenol A (DGEBA), was supplied by Huntsman (USA) as the technical product Araldite F and was used as received. The number-average molecular weight (*M*_*n*_) of this technical product is approximately 385 g/mol, calculated from the epoxy-equivalent value provided in the manufacturer’s datasheet. Araldite F is widely used in electrical engineering as a matrix resin for electrical insulating systems, including windings of rotating machines. Therefore, it was selected because of the intended electrical insulation application of the developed materials.

The amine curing agents were poly­(oxypropylene) α,ω-diamines (hereafter referred to as Diamines) sold under the trade name Jeffamine (Sigma-Aldrich, USA). Three grades were used: D230 (*M*_*n*_ = 230 g/mol), D400 (*M*_*n*_ = 430 g/mol), and D2000 (*M*_*n*_ = 1968 g/mol). All were used as received. The different poly­(oxypropylene) chain lengths enabled systematic control of the stiffness of the permanent EP network, including its glass-transition temperature, rubbery modulus, and network mesh size.

The bifunctional dienophile used for the synthesis of the reversible Diels–Alder network was 1,1′-(methylenedi-4,1-phenylene)­bismaleimide (BMI), obtained from Sigma-Aldrich (USA) and used as received. The second DA-network component, a tetrafunctional diene, was synthesized in-house from commercially available reagents (see below). Three tetrafunctional diene derivatives, abbreviated as FD, were prepared from the above-mentioned Diamines and furfuryl glycidyl ether (FGE, from Sigma-Aldrich, USA), which was also used as received. FGE underwent a 4-fold irreversible epoxy–amine addition reaction with Diamines D230, D400, and D2000, yielding the corresponding tetrafuran derivatives FD230, FD400, and FD2000. During the synthesis of the FD precursors and of all DA networks, hydroquinone was used as a radical polymerization inhibitor. Hydroquinone was obtained from Sigma-Aldrich (USA) and used as received.

#### 2.1.2 Tetra-Functional Dienes Synthesis

The synthesis of the tetra-functional dienes FD230, FD400, and FD2000 has been described previously.^37^ Briefly, the reaction was performed under an argon atmosphere by mixing stoichiometric amounts of the corresponding diamine (D230, D400, or D2000) with furfuryl glycidyl ether (FGE) without a solvent. Hydroquinone (1 wt % of the reaction mixture) was added as a radical polymerization inhibitor. The reaction mixture was stirred at 100 °C and 500 rpm for 6 h. The synthetic route is shown in Figure 1a,b and also as simplified step 1 in the overall preparation procedure for the self-healing materials in Figure 1c. The structure of the synthesized products was confirmed by 1H NMR spectroscopy, as reported previously.^37^

**Figure 1:**
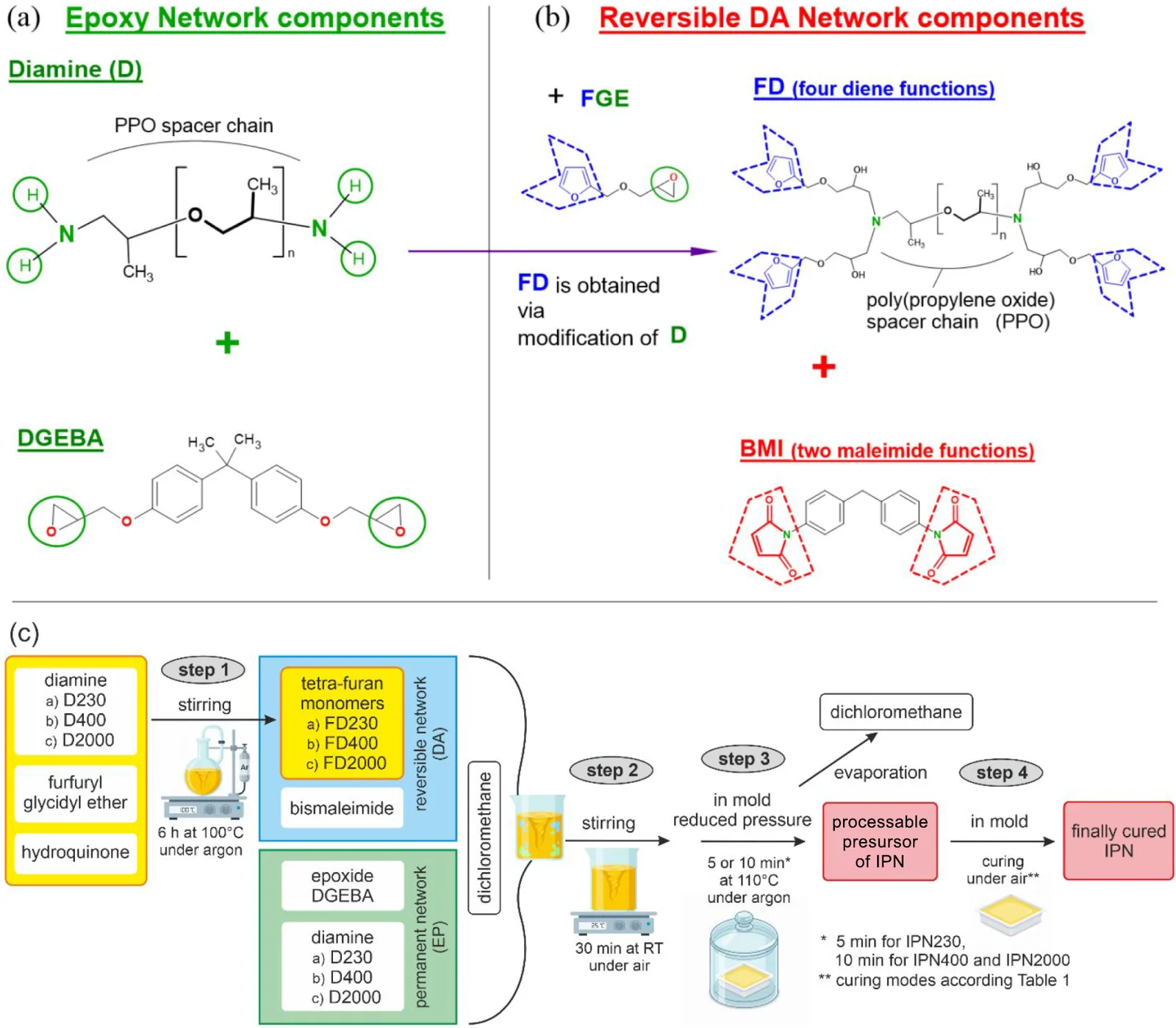
Structures of the chemical components of the (a) permanent network (DGEBA and diamine) and (b) the reversible Diels–Alder network (BMI and FD), including the synthesis of the tetra-functional diene component FD (tetra-furan) of the reversible network via modification of the diamine component of the permanent network (violet arrow); (c) schematic illustration of the preparation procedure for the studied self-healing materials.

#### 2.1.3 Synthesis of the Self-Healing Interpenetrating Networks

The synthesis of the studied self-healing materials comprising interpenetrating permanent (EP) and reversible (DA) networks is schematically illustrated in steps 2–4 in Figure 1c. The materials were synthesized by a one-pot process involving the simultaneous formation of both networks. The EP mixture consisted of DGEBA cured with Diamine (D230, D400, or D2000), whereas the DA mixture comprised bismaleimide (BMI) and the corresponding tetra-functional furan monomer (FD230, FD400, or FD2000), which formed the reversible network via the Diels–Alder reaction. The chemical structures of the reactants are shown in Figure 1a,b. The permanent epoxy–amine network maintains dimensional stability even at elevated temperatures, whereas the reversible DA network provides thermally activated self-healing. Upon heating, the DA network dissociates through the retro-Diels–Alder reaction, and it reforms during cooling, thereby reconnecting previously separated pieces of the material (see Figure 2a).

**Figure 2:**
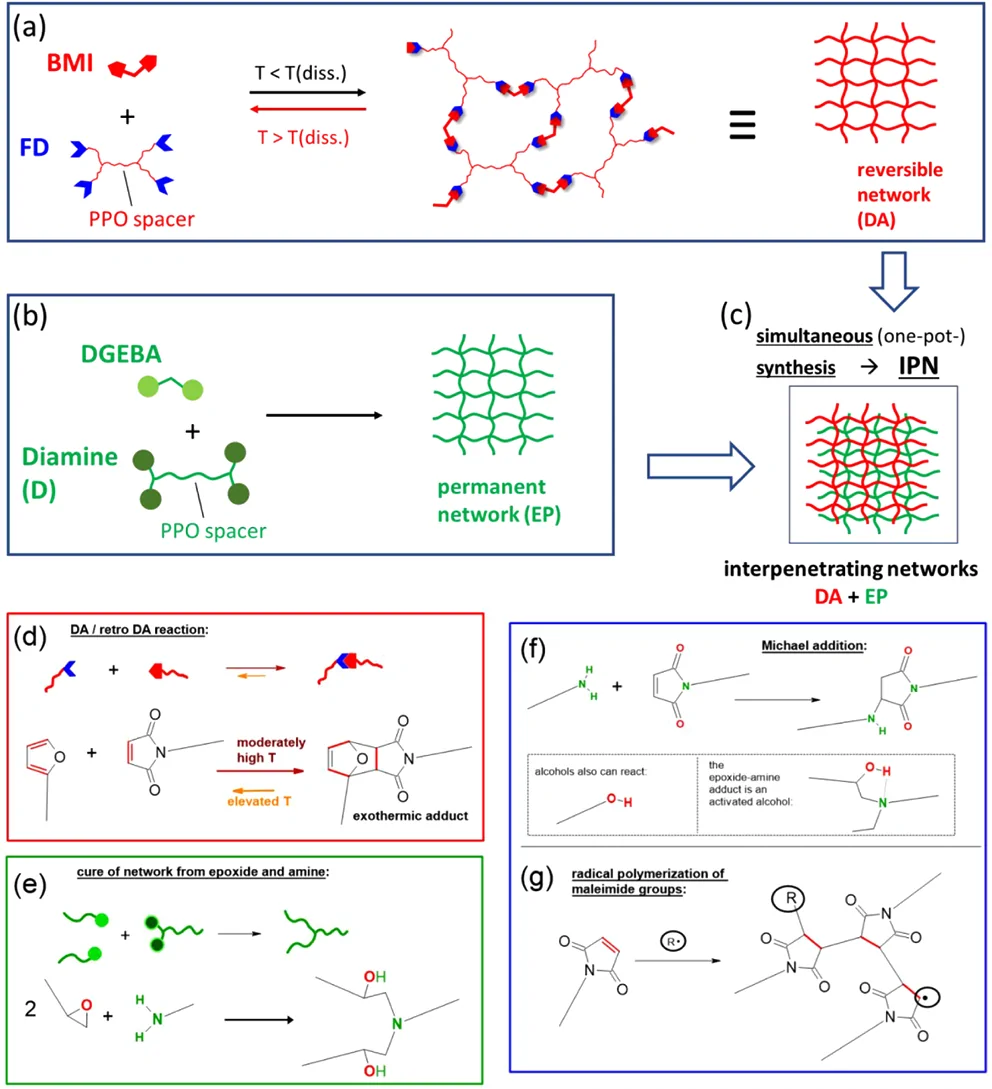
Formation of (a) reversible and (b) permanent networks, and (c) their combination into an interpenetrating polymer network. Desired reactions during curing: (d) reversible Diels–Alder cycloaddition between furan and maleimide groups, forming the reversible network, and (e) irreversible epoxy–amine curing, forming the permanent network. Undesired side reactions: (f) Michael addition between amine and maleimide groups and (g) radical polymerization of maleimide groups.

DGEBA (EP) and BMI (DA) are relatively small, rigid molecules (see Figure 1a,b). In contrast, Diamines (D230, D400, and D2000) and the corresponding FD230, FD400, and FD2000 monomers are based on flexible poly­(propylene oxide) chains. The shorter Diamines (D230 and D400) and their corresponding FD derivatives were selected to produce relatively rigid IPNs well suited for electrical insulation applications. Their rigidity is also expected to minimize dielectric losses under normal operating conditions despite the presence of polar ether groups. In contrast, the systems based on D2000 and FD2000, which contain long flexible chains and exhibit glass transition temperatures approximately 50 °C below room temperature, were investigated as model elastomeric systems. In these materials, (slow) thermally activated self-healing was expected to become observable even at room temperature.

The reactive functional groups, namely, the epoxide and amine groups in the EP system and the furan and maleimide groups in the DA system, were used in stoichiometric proportions. The mass ratio of the EP and DA mixtures was fixed at 1:2 for all IPN formulations. Dichloromethane (DCM) was used as the solvent to dissolve BMI, in an amount corresponding to ten times the BMI mass. In addition to the IPNs, reference materials consisting solely of the permanent or reversible network were also prepared.

At first, the EP and DA mixtures were combined and stirred at room temperature for 30 min (step 2 in Figure 1c). The resulting precursor was then poured into a Teflon mold preheated to 110 °C, and residual DCM was removed under reduced pressure at 110 °C for 5 min (D230 and D400 systems) or 10 min (D2000 system) (step 3 in Figure 1c).

Finally, the specimens were cured in the mold at atmospheric pressure (step 4 in Figure 1c) using different temperature–time programs (curing modes) to optimize the formation of both networks. The curing modes were designated a1–a3 (one-step curing) and b1–b4 (two-step curing). The one-step modes were a1, 90 °C for 24 h; a2, 90 °C for 34 h; and a3, 90 °C for 48 h. The two-step modes were b1, 120 °C for 2 h followed by 90 °C for 24 h; b2, 120 °C for 4 h followed by 90 °C for 16 h; b3, 120 °C for 4 h followed by 90 °C for 24 h; and b4, 120 °C for 4 h followed by 90 °C for 72 h. All prepared formulations and curing modes are summarized in Table 1.

**Table 1:** List of All the Synthesized Materials, Including a Description of Curing Modes

materials code	EP:DA networks ratio	curing agent for EP permanent network	FD type for DA reversible network	temperature (°C)/time (hours) of curing
EP230-a1	1:0	D230	-	90/24
EP230-a3	1:0	D230	-	90/48
EP230-b1	1:0	D230	-	120/2 + 90/24
EP230-b3	1:0	D230	-	120/4 + 90/24
EP400-a1	1:0	D400	-	90/24
EP400-a3	1:0	D400	-	90/48
EP400-b1	1:0	D400	-	120/2 + 90/24
EP400-b3	1:0	D400	-	120/4 + 90/24
EP2000-a1	1:0	D2000	-	90/24
EP2000-a3	1:0	D2000	-	90/48
EP2000-b3	1:0	D2000	-	120/4 + 90/24
EP2000-b4	1:0	D2000	-	120/4 + 90/72
DA230-a1	0:1	-	FD230	90/24
DA400-a1	0:1	-	FD400	90/24
DA2000-a1	0:1	-	FD2000	90/24
IPN230-a1	1:2	D230	FD230	90/24
IPN230-a3	1:2	D230	FD230	90/48
IPN230-b1	1:2	D230	FD230	120/2 + 90/24
IPN230-b3	1:2	D230	FD230	120/4 + 90/24
IPN400-a1	1:2	D400	FD400	90/24
IPN400-a2	1:2	D400	FD400	90/34
IPN400-a3	1:2	D400	FD400	90/48
IPN400-b1	1:2	D400	FD400	120/2 + 90/24
IPN400-b2	1:2	D400	FD400	120/4 + 90/16
IPN400-b3	1:2	D400	FD400	120/4 + 90/24
IPN2000-a1	1:2	D2000	FD2000	90/24
IPN2000-a3	1:2	D2000	FD2000	90/48
IPN2000-b1	1:2	D2000	FD2000	120/2 + 90/24
IPN2000-b4	1:2	D2000	FD2000	120/4 + 90/72

#### 2.1.4 Issues of Side Reactions and Mitigation of Their Effects

For the practical applicability of the selected IPN system, the formation of the permanent EP network and the reversible DA network must proceed independently without mutual interference during either curing or thermally induced self-healing at moderately elevated temperatures.

The desired reactions leading to IPN formation are schematically illustrated in Figure 2a–c and shown in detail in Figure 2d,e. The potential side reactions are presented in Figure 2f,g. These undesired reactions reduce the self-healing capability by creating irreversible covalent linkages between the EP and DA networks and by replacing reversible DA cross-links with permanent covalent bonds. Their occurrence was minimized by optimizing both the curing mode and the self-healing protocol (temperature and time).

### 2.2 Methodology

#### 2.2.1 Thermal Analysis

The thermal properties of the prepared materials were characterized by differential scanning calorimetry (DSC). DSC measurements were performed to evaluate the thermal transitions, heat-flow behavior, and curing state of the materials during repeated heating and cooling cycles.

DSC measurements were performed using a DSC Q2000 instrument (TA Instruments, New Castle, USA) at a heating/cooling rate of 10 °C min^–1^. Samples with a mass of approximately 9 mg were placed in sealed aluminum crucibles with a pinhole and measured in air at a flow rate of 50 mL min^–1^.

The viscoelastic and thermomechanical properties were investigated by dynamic mechanical thermal analysis (DMTA). This technique was used to evaluate the response of the materials to oscillatory shear deformation over a broad temperature range. The main DMTA parameters are the storage shear modulus (*G′*), loss shear modulus (*G″*), and mechanical loss factor (tan δ_m_), defined as the ratio of *G″* to *G′*. In this study, *G′* and tan δ_m_ were evaluated.

DMTA measurements were carried out using an ARES G2 rheometer (TA Instruments, New Castle, USA) equipped with a rectangular torsion geometry. The temperature dependence of the shear modulus was measured for rectangular specimens (30 × 6 × 1 mm) at a heating rate of 3 °C min^–1^ using oscillatory shear deformation at a frequency of 1 Hz.

Self-healing behavior was evaluated (among other methods) by repeated heating and cooling cycles using both DSC and DMTA measurements.

#### 2.2.2 Dielectric Analysis

Considering the intended application of the prepared self-healing materials as electrical insulation systems, their dielectric properties, including relative permittivity and dielectric dissipation factor (tan δ_d_), were investigated by broadband dielectric spectroscopy (BDS) over a wide frequency and temperature range.

BDS measurements were performed using an Alpha-A analyzer (Novocontrol Technologies, Montabaur, Germany) equipped with a ZGS capacitor electrode system for solid flat specimens. Samples were placed between cylindrical gold-plated electrodes with a diameter of 30 mm. The measurements were conducted in the frequency range of 0.5 Hz–1 MHz and at temperatures from −30 to 90 °C. The selected frequency range included 50 Hz, corresponding to the operating frequency of electrical machines, while the temperature range represented typical service conditions.

The dielectric response was obtained from impedance measurements using an applied voltage with an effective value of 1 V. The complex relative permittivity was calculated from the measured impedance considering the electrode geometry and sample dimensions. The real and imaginary components of the complex permittivity correspond to the dielectric constant (ε′) and dielectric loss factor (ε″), respectively, while the dielectric dissipation factor was calculated as the ratio ε″/ε′.

#### 2.2.3 Self-Healing Process Analysis

The efficiency of the self-healing process was evaluated by tensile testing and optical microscopy using specimens mechanically damaged by being cut into two separate parts.

*Static mechanical properties* were measured using an Instron 6025/5800R testing machine (Instron Limited, UK) equipped with a 100 N load cell at room temperature. Tensile tests were performed at a crosshead speed of 10 mm min^–1^ using dumbbell-shaped specimens with a total length of 15 mm, a narrowed section length and width of 5 and 3.2 mm, respectively, and a thickness of 1 mm. The reported table values represent the average of three independent measurements.

Optical analysis of the healing process was performed using a Dino-Lite Premier2 digital microscope (AD-4013MZT) with a resolution of 1.3 MP and magnification factors of 40× and 250×.

*Microcrack* *healing* experiments were performed using IPN400 and EP400 as reference materials. Both systems are glassy at room temperature, while IPN400 represents the most promising self-healing material developed in this work for dielectric applications. All specimens had a thickness of 1.25 mm. IPN400 samples were prepared as circular disks with a diameter of 25 mm, whereas EP400 reference specimens had a square geometry with a side length of 15 mm. Microcracks were generated by repeated bending of the specimens, until visible damage was observed by optical microscopy.

The healing treatment of IPN400 and EP400 samples was performed under the optimized conditions established for IPN400: heating at 140 °C for 30 min in an oven.

*Electrical breakdown* tests were performed in oil to prevent surface flashover, as illustrated in Scheme 1. The upper electrode was needle-shaped, whereas the lower electrode was cylindrical with a diameter of 10 mm. IPN400 and EP400 specimens with the same dimensions as those used in the microcrack healing experiments were placed between the electrodes. The applied voltage was increased continuously from 0 to 25 kV at a rate of 0.4 kV s^–1^.

**Scheme 1:**
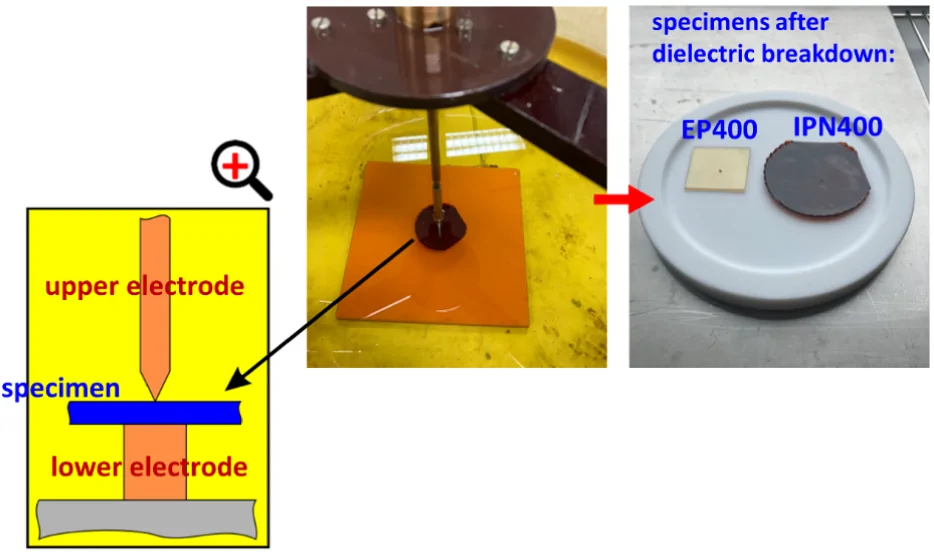
Schematic Setup of the Dielectric Breakup Experiments (Left), the Real Installation Immersed in Oil (Center), and Exemplary Specimens Shown after Enduring Dielectric Breakup Damage

After electrical breakdown, the damage location was documented by optical microscopy (Dino-Lite Premier2 digital microscope AD-4013MZT) from both the top and bottom surfaces of the specimens.

*Electrical treeing experiments* were performed on polymer specimens sized 10 × 10 × 4 mm^3^. During testing, they were positioned vertically on their 10 × 4 mm^2^ face, on a flat counter-electrode. Two systems were investigated, IPN2000 (self-healing interpenetrating network) and EP2000 (an analogous conventional epoxy lacking the self-healing capability). A sharp-tipped stainless-steel needle with a diameter of 0.25 mm served as the upper electrode, and was pressed into the volume of the tested polymer, so that the distance between its tip and the flat counter-electrode at the bottom of the specimen was ca. 3 mm (see the setup in Scheme 2). The needle tip precisely defined the initiation site of the electrical tree growth.

**Scheme 2:**
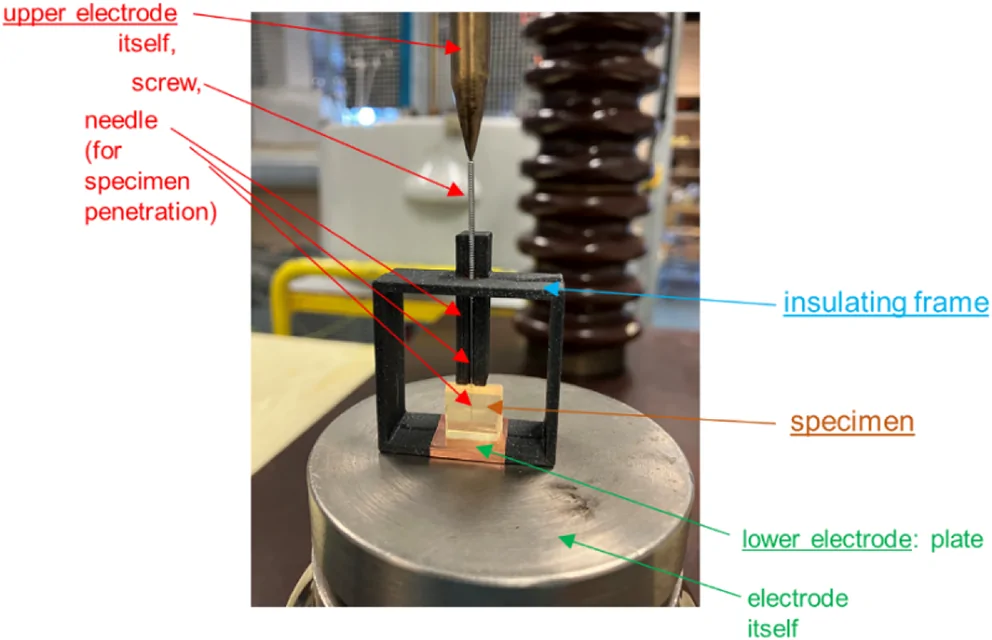
Setup of the Electrical Treeing Experiments

Electrical tree growth was initiated by applying an alternating (50 Hz) voltage with a root-mean-square value of 9.5 kV. Under these conditions, electrical propagation was monitored optically, and the experiment was intentionally terminated before dielectric breakdown could occur.

Healing of the electrical treeing damage was performed by transferring the respective specimen (with the needle left in place) to a forced-circulation air oven, and subjecting it to the optimized thermal-healing protocol corresponding to the respective tested material (120 °C for 30 min, for both IPN2000 and the EP2000 reference system).

The morphology of the electrical trees before and after the thermal healing treatment was examined using an optical microscope (Dino-Lite Premier2 digital microscope, type AD-4013MZT) operated in transmitted light mode.

## 3 Results and Discussion

### 3.1 Curing Mode Optimization

The studied self-healing interpenetrating epoxy networks (IPNs) were optimized to maximize both the shape stability and the dynamic bonding provided by the EP and DA subnetworks. The employed curing mode (i.e., the temperature–time curing protocol) was optimized to prevent, or at least minimize, undesired side reactions at elevated temperatures, as illustrated in Figure 2f,g, while promoting efficient network formation and a high degree of cure in both constituent networks (Figure 2d,e). To this end, curing modes a1–a3 and b1–b4 were tested, which are described in Section 2 (last paragraph of Section 2.1.3). The success of the cure optimizations was preliminarily evaluated via thermomechanical and thermal analyses, while the suitability of the formulation as an insulating material was assessed through dielectric tests. Additionally, self-healing performance was investigated using combined healing-tensile tests, microcrack healing analysis, and the evaluation of electrical stress-induced damage recovery (as detailed in the following sections).

To achieve the formation of the studied interpenetrating EP and DA networks, three principal curing pathways can be considered (Figure 3), which served as the basis for designing the curing modes employed in this study.

**Figure 3:**
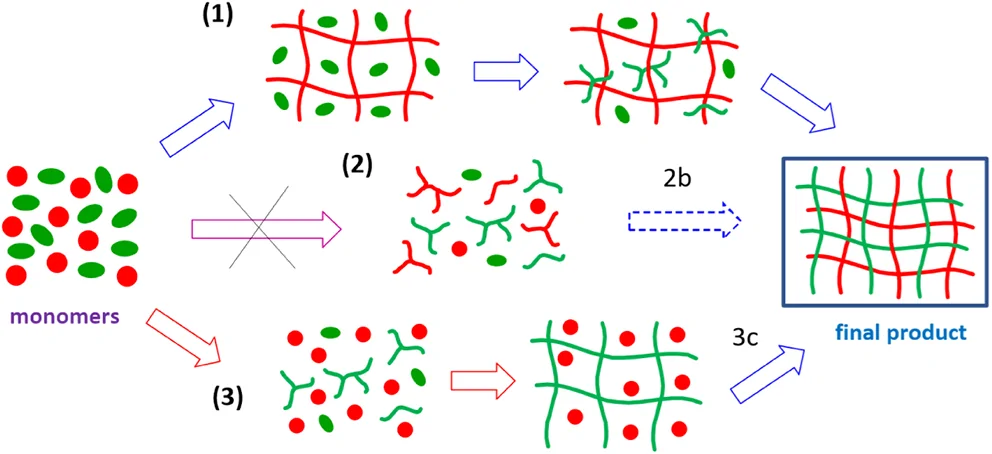
Three possible routes of curing the interpenetrating networks: (1) low-temperature cure—the reversible DA network (red) rapidly polymerizes, and the permanent EP network (green) grows slowly inside the cured DA; (2) simultaneous growth of both networks—difficult to achieve: temperature would need to be continuously adjusted; (3) high-temperature cure—the reversible DA network stays dissociated while the EP network rapidly grows, until in the final step the temperature is lowered and the DA network grows inside the EP one.

Route 1 in Figure 3 represents low-temperature curing, performed well below the dissociation temperature of the DA network (red), namely at 90 °C. Under these conditions, the DA network cures rapidly without undergoing dissociation or reorganization. In contrast, the EP network (green) develops considerably more slowly and therefore grows within the preformed DA network, where its development becomes spatially constrained. Furthermore, for the EP230 formulation, the EP network approaches vitrification during curing at this relatively low temperature, which reduces its chain-segment mobility and further hinders EP network formation. The curing modes a1–a3 (see Section 2) correspond to route 1.

In principle, route 2 in Figure 3 would enable the simultaneous growth of both constituent networks. However, such an approach is technically difficult to implement. The temperature would have to be continuously decreased from above the DA dissociation temperature (≥120 °C in the present study) to approximately 90 °C, where complete DA cross-linking occurs (see cooling in step 2b in Figure 3). Moreover, this temperature profile would need to be dynamically adjusted according to the real-time progress of EP network formation.

Route 3 in Figure 3 proved to be the most effective curing strategy and was therefore adopted in this work. Initially, the material is cured at approximately 120 °C, ensuring that the DA network remains fully dissociated while the EP network forms rapidly and without spatial constraints. After complete (ideal case) or nearly complete (practical case) cross-linking of the EP network, the system is cooled (step 3c in Figure 3) to approximately 90 °C, where the DA network forms (ideal case), while some remaining EP cross-linking reactions may also proceed additionally (practical case). This strategy formed the basis of the curing modes b1–b4 (see Section 2).

The high-temperature stage of route 3 was optimized to be sufficiently long to achieve a high degree of EP cross-linking, thereby ensuring dimensional stability, while remaining short enough to suppress excessive Michael addition. Excessive Michael addition would irreversibly couple the EP and DA networks, while also converting the reversible DA cross-links into permanent ones, and consequently eliminating the self-healing capability.

It should be noted that the reactivity of the amine hardener in the EP system (in both the desired epoxy–amine reaction and the undesired Michael addition) decreases with increasing length of the polypropylene oxide spacer between the terminal amino groups (see structure in Figure 1), following the order: D230 > D400 ≫ D2000. This trend arises from the decreasing molar concentration of reactive amino groups in the neat diamine hardeners as the fraction of chemically inert polypropylene oxide segments increases.

The suitability of the different curing modes was evaluated by dynamic mechanical thermal analysis (DMTA), as discussed below. The desired outcome was a material exhibiting a step-like thermoreversible decrease in storage modulus (*G*′) above 120 °C, corresponding to the reversible dissociation of the DA subnetwork, together with pronounced dimensional stability (observable as a plateau in *G*′ = f­(*T*), with no melting) at *T* > 120 °C.

### 3.2 Thermomechanical and Viscoelastic Behavior

Dynamic mechanical thermal analysis (DMTA) was employed to characterize the thermomechanical behavior of the prepared IPNs, with particular emphasis on distinguishing the contributions of the permanent EP network and the reversible DA subnetwork. The corresponding neat EP (permanent) and DA (reversible) networks were used as reference materials. The glass-transition temperature (*T*_g_), the temperature of DA bond dissociation (*T*_diss_), the possible occurrence of melting following DA dissociation, and the repeatability of the dissociation/recombination process were evaluated. On the basis of the DMTA results, the optimal IPN compositions and curing protocols were identified, namely, IPN400-b2 and IPN2000-b4, which exhibited shape stability alongside prominent thermally dissociable cross-linking, a feature essential for self-healing.

Permanent EP (epoxy–amine) networks display a thermomechanical behavior that is summarized in Figure 4a,b, where EP230, EP400, and EP2000 are compared. A more detailed analysis is provided in the Supporting Information. All EP networks were prepared from the rigid aromatic diepoxide DGEBA (see Figure 1) and differed only in the α,ω-diamine curing agent, whose polypropylene oxide (PPO) spacer contained 4, 8, or 33 repeat units, respectively. Increasing the spacer length increases chain flexibility while simultaneously reducing the cross-link density of the EP network. Consequently, both the rubbery storage modulus and *T*_g_ decrease progressively from EP230 to EP2000. Notably, the curing protocol had little influence on the thermomechanical response of the EP networks (see Figure S1 and associated detailed comments).

**Figure 4:**
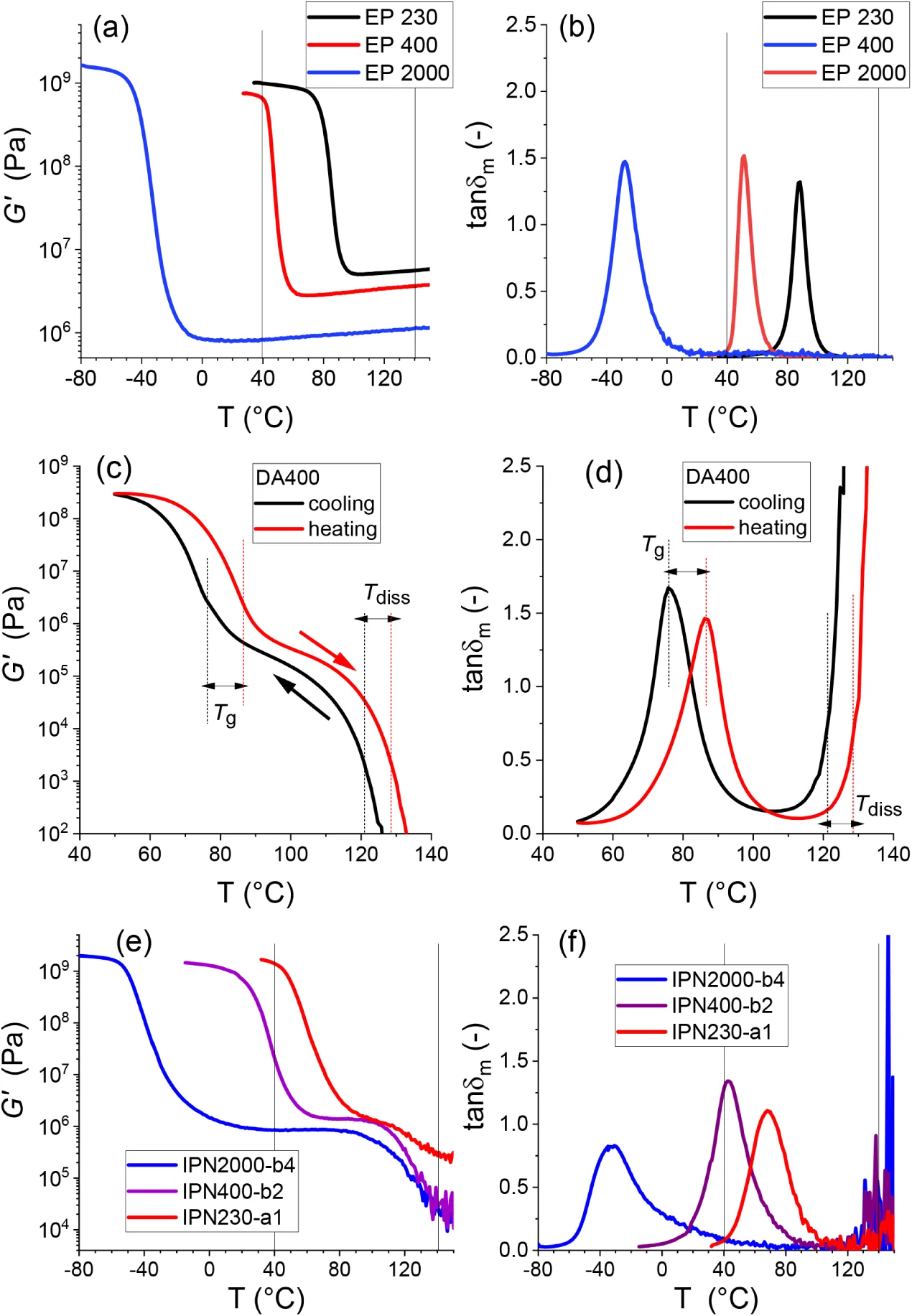
DMTA profiles of (a, b) pure permanent EP networks EP230, EP400, and EP2000; (c, d) pure reversible DA network DA400; (e, f) interpenetrating networks IPN230, IPN400, and IPN2000; temperature dependencies of (a, c, e) storage modulus *G′* and (b, d, f) loss factor tan δ_m_. The temperature scale of the DMTA profiles of DA400 in graphs (c, d) is narrower than in the case of EP and IPN—hence the vertical thin lines in graphs (a, b, e, f) indicate the depicted temperature window of DA.

Neat reversible DA networks display a thermomechanical behavior that is compared in Figure 4c,d (DA400) and Figure S2 (the remaining DA systems; detailed discussion also is provided there). Similar to the EP systems, complete network formation was readily achieved, and the curing protocol a1 (24 h at 90 °C) was sufficient to obtain fully cross-linked products.

Unlike the EP networks, the DA systems exhibit not only a distinct glass transition but also a thermally induced melting transition associated with dissociation of the DA adducts. This process appears as a second drop in the storage modulus (Figure 4c) and a sharp increase in tan δ_m_ (Figure 4d). Another characteristic feature is the pronounced thermal hysteresis between heating and cooling scans (performed at 3 °C/min; see Figure 4c,d: red vs black curves), reflecting the finite kinetics of the retro-Diels–Alder/Diels–Alder recombination process (see ref 36).

Both *T*_diss_ and, more markedly, *T*_g_ decrease with increasing PPO spacer length of the FD precursor (see trend lines in Figure S2f). At the same time, the hysteresis associated with DA bond dissociation becomes substantially larger from DA400 to DA2000 (Figure S2f). As expected, all DA networks undergo complete melting near *T*_diss_, as evidenced by an abrupt collapse of the storage modulus and a nearly vertical rise in tan δ_m_.

The *T*_g_ values of the DA networks were generally higher than those of the corresponding EP analogues. For DA2000, however, the glass transition is split into two relaxation processes (see Figure S2c,d). The dominant transition (*T*_g1_) appears as a broad peak with maxima at 13 and 36 °C (average approximately 20 °C), while a second, less intense transition (*T*_g2_) occurs at 42 °C). This behavior suggests partial microphase separation within the DA2000 network.

The rubbery storage moduli of the DA networks were consistently lower than those of the corresponding EP systems. Comparison of Figure 4a,b with Figure 4c,d and Figure S2 indicates that the DA networks, particularly those based on shorter PPO spacers, do not achieve quantitative cross-linking, most likely because steric constraints limit the efficiency of the Diels–Alder reaction. Accordingly, the rubbery plateau of the DA networks is less well defined and gradually decreases with increasing temperature.

The IPN systems prepared using different curing modes display DMTA responses which are compared in Figure 5 (IPN400), Figures S3 and S4 (IPN230–IPN2000). A comprehensive discussion is provided in the Supporting Information. Overall, the two-step curing protocols clearly outperformed the one-step procedures. Among the investigated materials, IPN400-b2 emerged as the most promising candidate for self-healing electrical insulation, combining efficient and repeatable self-healing with a moderate *T*_g_ of approximately 43 °C. The more flexible IPN2000-b4 also exhibited excellent self-healing performance while maintaining sufficient permanent cross-linking; however, its *T*_g_ of approximately −40 °C resulted in a rubbery material at room temperature. In contrast, the IPN230 system proved unsuitable for self-healing. Only IPN230-a1 retained a DMTA-detectable fraction of reversible DA cross-links, whereas all other curing protocols resulted in predominantly permanent cross-linking owing to side reactions that irreversibly coupled the EP230 and DA230 subnetworks.

**Figure 5:**
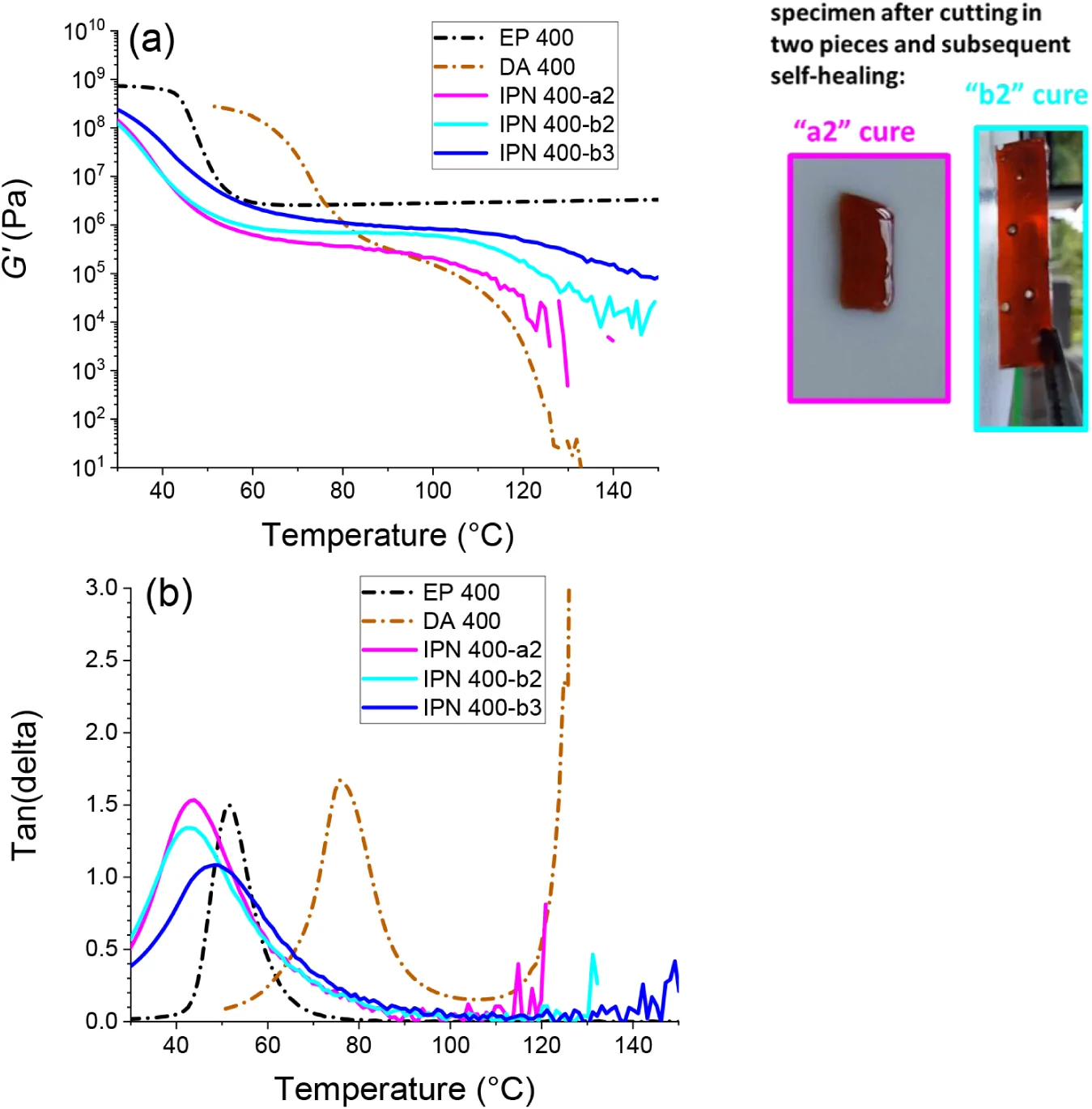
Effect of curing mode on the DMTA profile of IPN400; EP400-b3 and DA400-a1 are shown as reference materials: temperature dependence of (a) storage modulus G′ and (b) loss factor tan δ_m_; inlay photographs: samples IPN400-a2 and IPN400-b2 after disruption (by cutting) and subsequent self-healing at 140 °C for 30 min.

In general, the *T*_g_ values and transition shapes of the IPNs closely resemble those of the corresponding EP networks. Unlike the neat DA systems, the IPNs do not exhibit measurable thermal hysteresis during heating and cooling. Their rubbery storage moduli are intermediate between those of the EP and DA reference networks, while the rubbery plateau remains well defined, approaching the behavior observed for the permanently cross-linked EP materials.

### 3.3 Curing Protocol Effect on the IPNs Thermomechanical Behavior

The chosen curing mode (one-step modes at 90 °C in variants a1–a3 and two-step curing at 90/120 °C in variants b1–b4, see Table 1) has a profound influence on the thermomechanical behavior of the IPNs (Figure 5, Figures S3 and S4), in contrast to the corresponding neat EP and DA networks. Because of the side reactions discussed above (particularly the Michael addition of hydroxyl groups generated during epoxy curing to the dissociated BMI species, see Figure 2f), the optimal curing mode must provide the highest attainable degree of cross-linking of the permanent EP subnetwork, thereby ensuring dimensional stability, while simultaneously minimizing high-temperature side reactions that irreversibly cross-link and covalently couple the reversible DA subnetwork, which is essential for self-healing.

Optimally cured IPNs exhibit thermomechanical behavior that combines features of both constituent networks. A key difference from the neat DA systems is that the second modulus drop, associated with DA bond dissociation, should be followed by a second rubbery plateau (only its initial region below 150 °C could be measured), rather than by the continuous modulus decrease characteristic of the neat DA networks. This second plateau originates from the permanently cross-linked EP network, which remains intact after dissociation of the reversible DA subnetwork.

Accordingly, optimization of the curing protocol was based primarily on the shape of the second modulus drop. A continuous melting-like decrease, similar to that observed for the neat DA network, indicated insufficient cross-linking in the EP subnetwork, whereas a weak or completely absent second modulus drop suggested excessive irreversible cross-linking in the DA network caused by side reactions. Because the concentrations and mobilities of the reactive functional groups differ substantially in IPN230, IPN400, and IPN2000, different curing protocols were required for each formulation.

For IPN400, the effects of selected curing modes (a2, b2, and b3) on its DMTA profiles are analyzed in Figure 5. Results obtained using additional curing variants (a1, a3, and b1) are presented in Figure S4c,d. The curing modes a3 and b3 produced materials showing only a weak second modulus transition, and the corresponding self-healing experiments (30 min at 120 °C) resulted in poor reconnection of the cut specimens. Samples prepared using modes a1, a2, and b2 exhibited promising thermomechanical characteristics; however, IPN400-a1 melted above 120 °C, whereas IPN400-a2 still exhibited slight flow at this temperature (see inlay in Figure 5). In contrast, IPN400-b2 exhibited complete dimensional stability together with efficient healing after thermal treatment (Figure 5, inlay), making it the optimal product within the IPN400 series.

IPN230 contains the highest concentration of reactive functional groups among the investigated IPN systems, which results in very high rates of both the desired curing reactions and the undesired Michael addition, as documented by DMTA results in Figure S3a,b and Figure S4a,b. Only IPN230-a1 retains a weak second modulus transition, indicating the presence of a limited fraction of reversible DA cross-links. However, its DMTA response suggests an inferior self-healing performance compared to the already unsatisfactory IPN400-b3 formulation. Attempts to apply even milder curing conditions resulted in incompletely cured materials that partially melted during DMTA while simultaneously undergoing irreversible cross-linking through Michael addition. Overall, the high intrinsic reactivity of the IPN230 components made this formulation difficult to process successfully.

IPN2000 contains the lowest concentration of reactive functional groups (in contrast to IPN230), which leads to slower formation of both the EP and DA subnetworks. Consequently (as illustrated by DMTA results in Figures S3 c,d and S4 e,f), a more energetic two-step curing protocol (b4), involving prolonged dwell times at both 120 and 90 °C, was required to achieve both dimensional stability and efficient self-healing. Whereas self-healing was easily achieved in IPN2000, obtaining a sufficiently cross-linked permanent EP subnetwork that prevents melting proved considerably more challenging.

#### 3.3.1 Cyclic DMTA Tests: Assessing Repeatable Self-Healing Capability

The DMTA analyses in Figures 4 and 5 (above) seem to indicate that 120 °C is the appropriate healing temperature for all the promising IPN systems. This prediction was confirmed experimentally for IPN2000-b4, where complete healing of cut specimens was achieved within approximately 30 min. In contrast, IPN400 required a higher healing temperature, as discussed further below.

To evaluate the long-term stability of the reversible DA subnetwork and the self-healing repeatability, cyclic heating–cooling DMTA experiments with a maximum temperature of 150 °C were conducted (Figure 6a). Particular attention was paid to possible irreversible changes arising from the Michael addition between hydroxyl groups in the cured EP network and dissociated BMI species (Figure 2f). This test evaluated the repeatability of the self-healing response.

**Figure 6:**
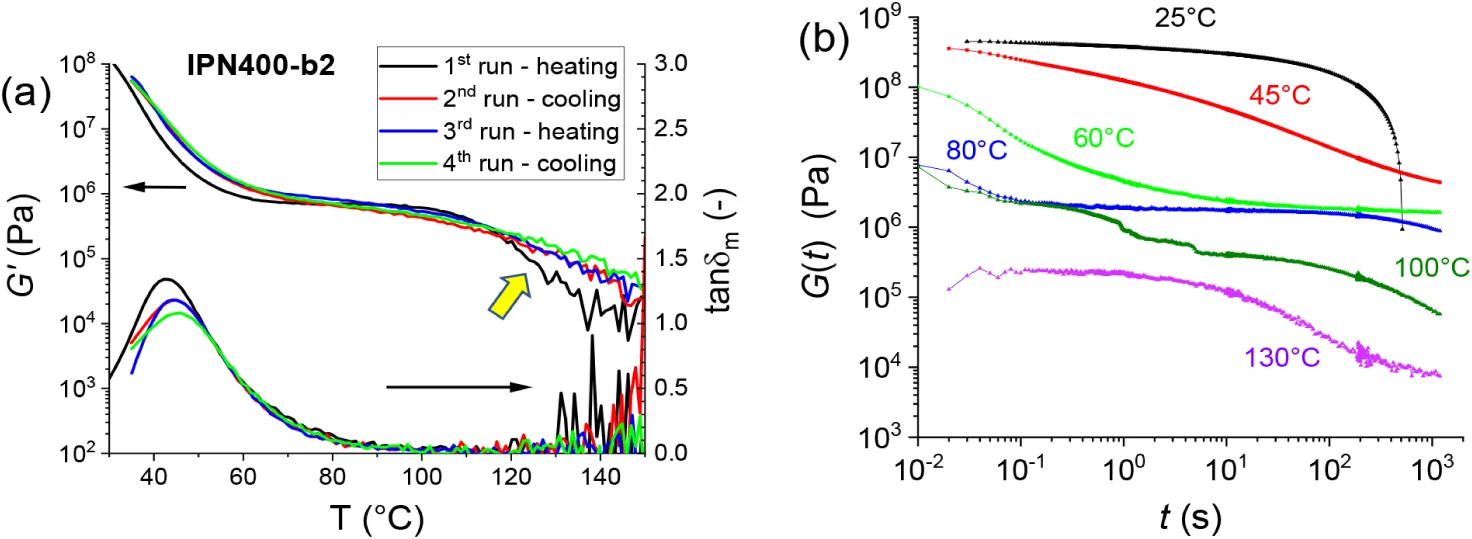
Reversible cross-linking analyzed by DMTA and relaxation tests: (a) cyclic heating/cooling tests with the sample IPN400b2, between 30 and 150 °C; depicted are the temperature-dependent curves of the storage modulus *G*′ (a: top) and of the loss factor tan δ_m_ (a: bottom); the yellow arrow in (a) highlights the region of reversible cross-linking; (b) stress relaxation tests at different temperatures: time-dependent relaxation modulus.

IPN400-b2, the most promising formulation, showed the behavior presented in Figure 6a. After the first heating cycle to 150 °C, the retro-Diels–Alder transition became slightly less pronounced, but no further changes were observed during subsequent heating–cooling cycles. This initial change is likely associated with either residual curing of the EP network or a limited extent of irreversible Michael addition. Interestingly, the neat DA networks also undergo slight structural rearrangement during repeated DMTA cycling, manifested by an upward shift of the DA dissociation and recombination temperatures, while their glass-transition temperature remains essentially unchanged (Figure S5a,b). The *T*_g_ transition of DA continues to exhibit the above-mentioned characteristic thermal hysteresis between heating and cooling scans, also in the case of the cyclic tests.

IPN2000-b4, which is more flexible, was also subjected to cyclic DMTA analysis (see Figure S5c). In this case, the DA/retro-DA transition remained essentially unchanged throughout repeated heating–cooling cycles, demonstrating excellent stability of the reversible subnetwork. This behavior is attributed to the lower concentration of reactive functional groups, which reduces the probability of irreversible side reactions.

Stress relaxation measurements were performed to evaluate the creep resistance of IPN400-b2 at characteristic temperatures (see Figure 6b). At 25 °C, the material is already within the onset of its glass transition (see DMTA in Figure 6a): The storage modulus remains initially constant and subsequently relaxes over several hundred seconds. At 45 °C, corresponding to the later stage of the glass transition, the relaxation modulus approaches the equilibrium rubbery value within approximately 100 s. At 60 °C, near the upper limit of the rubbery plateau, relaxation is completed within approximately 0.1 s, followed by a stable rubbery modulus, indicating the absence of detectable DA bond dissociation over the experimental timescale (1000 s). At 100 °C, just above the rubbery region, the progressive dissociation of weaker DA bonds together with partial dissociation of the stronger DA cross-links becomes evident as two distinct relaxation steps. Finally, at 130 °C, within the main DA dissociation region identified by DMTA (see Figure 6a), the strongest DA cross-links dissociate within approximately 100 s, after which the relaxation modulus reaches a stable low-value rubbery plateau governed by the permanent EP subnetwork.

### 3.4 Thermal Analysis of the Curing Process and Final Polymers

The thermal transitions of the prepared materials were investigated by differential scanning calorimetry (DSC). The results are presented in Figure 7 and Figures S6–S9. Figure 7a compares the DSC thermograms of the most promising interpenetrating network IPN400-b2 with those of the fully reversible DA400-a1 network and of the permanently cross-linked EP400-b3 network, which represent the two constituent subnetworks of IPN400-b2.

**Figure 7:**
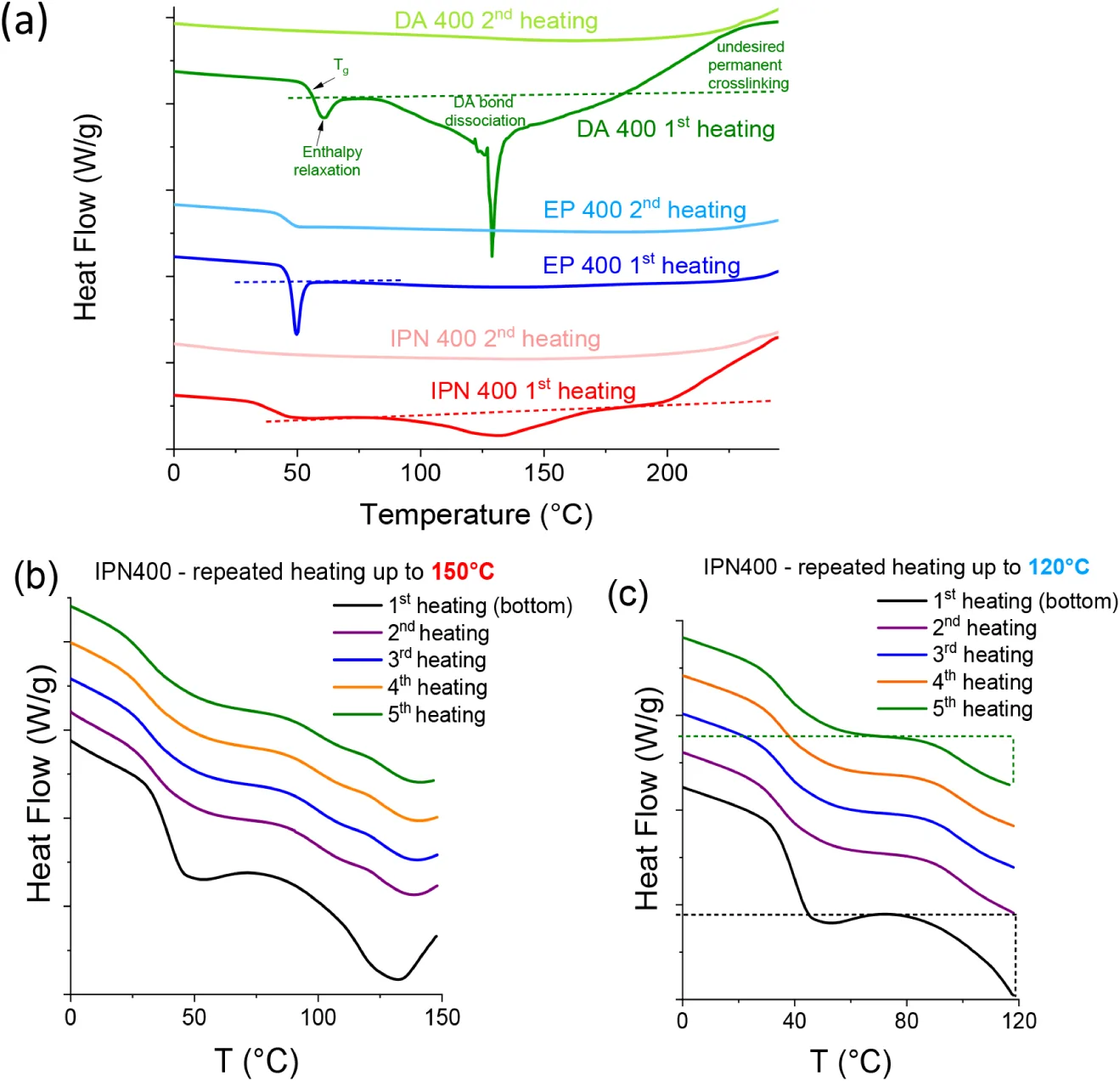
DSC curves: (a) thermograms of the pure reversible network DA400-a1 (top: light/dark green), of the pure permanent network EP400-b3 (center: light/dark blue), and of the related interpenetrating network IPN400-b2 (bottom: light/dark red); the heating scans were performed up to 250 °C; the respective lighter lines represent the 1st heating scans, the darker ones the respective 2nd heating scans; (b, c) results of the repeated heating of the most promising interpenetrating network IPN400-b2: (b) up to T = 150 °C and (c) up to T = 120 °C; cooling traces are omitted for clarity in (a–c).

DA400-a1, the pure reversible network, displays four characteristic processes in DSC (see Figure 7a and Figures S6–S8): The first is the glass transition, observed as a baseline step near 56 °C, immediately followed by an endothermic enthalpy-relaxation peak centered at 61 °C. This peak originates from the recovery of the physically aged glassy state and is commonly attributed to the reversal of structural densification during aging (see, e.g., refs 40,41 ). The third feature is a broad endothermic transition, beginning at 84 °C, which corresponds to dissociation of the Diels–Alder adducts. Its maximum extends between 122 and 132 °C, with a distinct sharp subpeak at 129 °C that is observed only during the first heating scan (Figure S6a). Finally, above ca. 180 °C, a broad exothermic region is observed, with heat evolution becoming particularly pronounced above 200 °C. The onset of this exothermic process overlaps with the completion of DA bond dissociation. It is attributed to irreversible Michael addition together with radical polymerization of the dissociated DA species, resulting in permanent cross-linking and irreversible loss of self-healing capability (see Figure 2f,g above).

The irreversible nature of the latter reaction is confirmed by the second heating scan of the same DA400-a1 specimen after exposure to 250 °C (see Figure 7a). The glass-transition step becomes markedly less pronounced, indicating a less-ordered network structure, while the DA dissociation peak disappears completely. Only a weak residual exothermic signal remains above 180 °C, confirming that nearly all reactive DA groups were destroyed during the first heating cycle.

IPN400-b2, an interpenetrating double network, exhibits most of the characteristic thermal features of the DA400-a1 subnetwork (see Figure 7a, Figures S7 and S8), including a glass transition at approximately 40 °C, although without the adjacent enthalpy-relaxation peak, followed by a broad endothermic peak associated with DA bond dissociation (onset at 88 °C, flat summit between 127 and 135 °C) and an exothermic region corresponding to irreversible destruction of the DA groups above approximately 190 °C. Similar to DA400-a1, the second heating scan after exposure to 250 °C becomes essentially featureless, demonstrating that the reversible DA functionality has been almost completely eliminated by the high-temperature treatment.

Self-healing in principle could be expected over the entire temperature range between the glass transition and the onset of the undesired irreversible cross-linking of DA. From a practical standpoint, however, for achieving practically relevant healing times, the most favorable healing conditions are located within the DA dissociation region, particularly between approximately 110 and 130 °C. This temperature window provides sufficiently fast Diels–Alder bond exchange while remaining well below the onset of irreversible Michael addition and radical polymerization.

EP400-b3, the pure permanent network, unlike its DA analogue, exhibits only a single persistent thermal transition, namely the glass transition at approximately 46 °C (see Figure 7a). This transition remains essentially unchanged even after heating to 250 °C, demonstrating the excellent thermal stability of the permanently cross-linked EP network (see the second heating scan in Figure 7a). Similar to DA400-a1, EP400-b3 also displays an enthalpy-relaxation peak adjacent to *T*_g_ (approximately 50 °C), which disappears during the second heating scan (see Figure S9).

The thermal stability of the reversible DA subnetwork under repeated heating was further evaluated by cyclic DSC experiments (see Figure 7b,c and Figures S6–S8). Figure 7b shows repeated heating scans of IPN400-b2 up to 150 °C, whereas Figure 7c presents analogous experiments with a maximum temperature of 120 °C. For clarity, only the heating scans are shown. The endothermic DA dissociation peak gradually decreases in area during repeated cycling; however, this decrease is considerably smaller when the maximum temperature is limited to 120 °C. The neat DA400-a1 network exhibits an analogous trend (Figure S6).

The tolerance of the IPN systems under self-healing conditions is discussed in greater detail in Section 3.5. Briefly, heating at 140 °C enables rapid and efficient healing of IPN400-b2, whereas at 120 °C it is substantially less effective. Figure S7a compares destructive DSC scans (up to 250 °C) recorded for a freshly prepared (intact) specimen of IPN400-b2 and for an identical specimen previously subjected to the optimal healing treatment (140 °C for 30 min). Only a slight change in the shape of the DA dissociation peak is observed, while its integrated area remains essentially unchanged. Likewise, the exothermic peak associated with irreversible side reactions is unaffected, indicating that the vast majority of DA groups survive the healing treatment. The analogous behavior of the neat DA400-a1 network is shown in Figure S7b. The proven preservation of the DA groups is essential because it enables repeated self-healing cycles without significant degradation of the material.

The thermal degradation of the DA functionality is further analyzed in Figure S8a. Destructive DSC scans (up to 250 °C) were recorded for IPN400-b2 specimens that had previously been heated, without an isothermal dwell, to maximum temperatures ranging between 120 and 150 °C. As the maximum temperature increased above 120 °C, a gradual decrease was observed in both the endothermic peak associated with DA bond dissociation and the exothermic peak corresponding to the irreversible polymerization of the dissociated DA species. The neat DA400-a1 network exhibited the same trend (see Figure S8b).

### 3.5 Analysis of Dielectric Properties

The dielectric performance of the developed materials was evaluated in terms of the relative permittivity (ε′) and dielectric dissipation factor (tan δ_d_) over a broad frequency and temperature range representative of potential electrical insulation applications. This comprehensive analysis enabled comparison of the reference permanent EP networks with the corresponding interpenetrating EP/DA networks, while aiming to identify IPN formulations combining low ε′ with the lowest possible tan δ_d_ over the investigated operating window. The IPN400 systems were found to possess the most attractive dielectric properties among the IPNs capable of efficient self-healing.

EP230 exhibited the most favorable dielectric performance among the permanent EP reference networks. Its dielectric constant remained below 10 even under the most demanding measurement conditions (80 °C and 0.5 Hz), while tan δ_d_ remained below 1, corresponding to a loss angle smaller than 45°, which represents a practical upper limit for insulating materials. In practice, however, high-performance electrical insulation requires dissipation factors in the range of 0.001–0.01, values that were achieved by several of the investigated materials within specific frequency and temperature intervals.

For EP400 and EP2000, both ε′ and tan δ_d_ increased markedly at elevated temperatures and lower frequencies. This behavior is closely related to their progressively lower glass-transition temperatures. Because *T*_g_ of EP230 lies near the upper limit of the investigated temperature range, its dielectric response was measured practically exclusively in the glassy state. In contrast, EP2000 was characterized predominantly above its *T*_g_, where enhanced molecular mobility increases dipolar polarization and, at higher temperatures, also the contribution of electrical conductivity. To sum up, the progressively lower *T*_g_ values of EP400 and EP2000 result in gradually increasing electrical conductivity noticeable at higher temperatures.

The above-discussed differences among the permanent EP networks strongly influence the dielectric properties of the corresponding IPNs. Although *T*_g_ and the dielectric response can be adjusted to some extent by modifying the curing mode, the curing conditions were primarily optimized to preserve the reversibility of the DA network. Therefore, the selected curing modes represent a compromise between the dielectric performance and self-healing capability.

The temperature–frequency dependence of the dielectric dissipation factor for all investigated IPN materials is summarized in Figure 8. The polarization positions associated with the glass transition are indicated by the orange arrows. Because the dissipation factor proved to be the most sensitive parameter for comparing the investigated systems, the corresponding relative permittivity maps are presented only in the Supporting Information (Figure S10).

**Figure 8:**
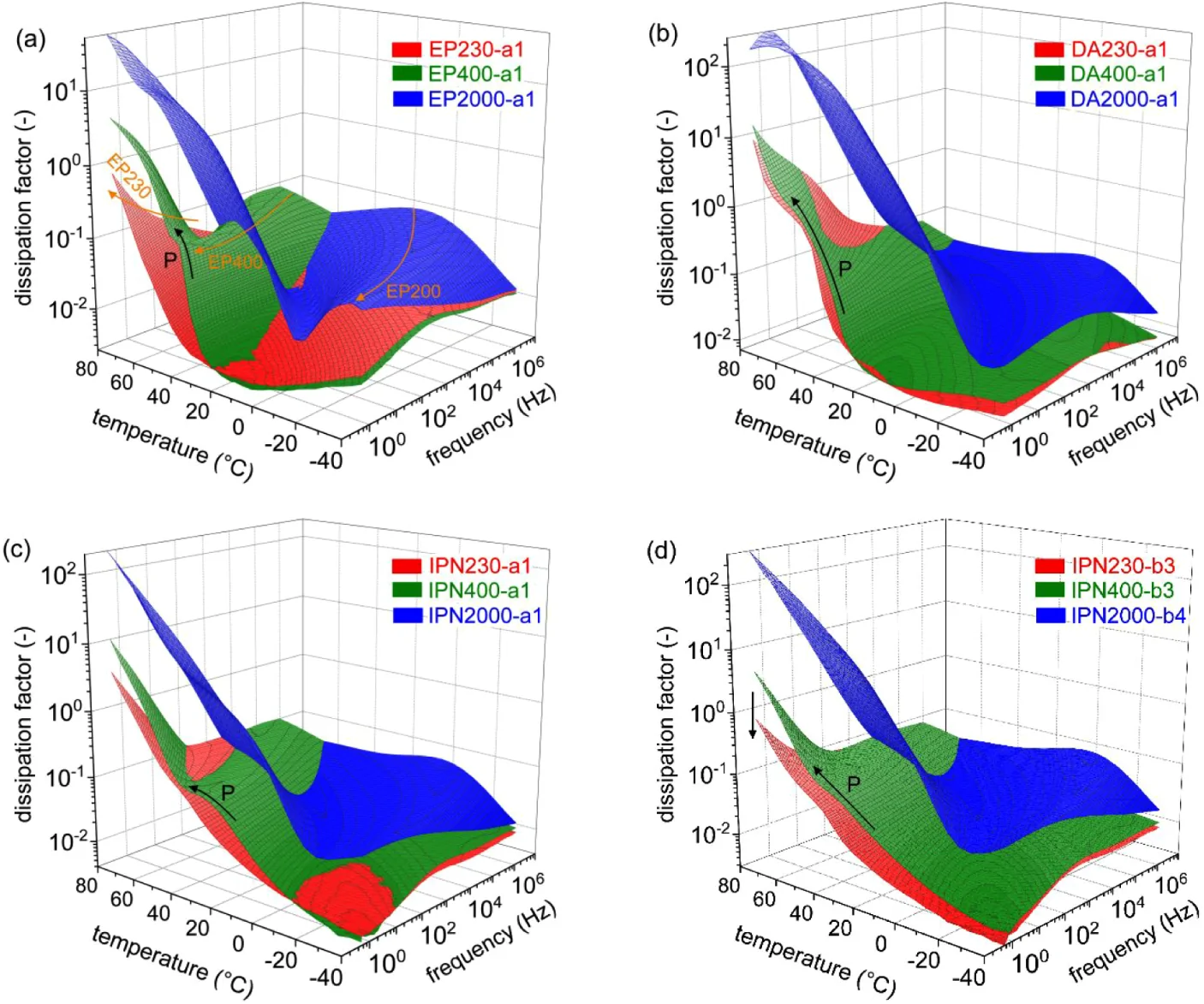
Frequency–temperature dependencies of dissipation factors for (a) EP, (b) DA, (c) IPN at the shortest curing mode a1, and (d) IPN at the most extended curing mode b3 or b4.

The influence of incorporating the reversible DA network into the permanent EP matrix, particularly in the polarization region marked P (black arrow), is illustrated in Figure 8c,d. Although the dielectric response of the IPNs is altered by the DA subnetwork, the properties of the EP subnetwork remain the dominant factor governing the overall dielectric behavior of the IPNs.

Comparison of the shorter (a1) and longer (b3, b4) curing protocols (Figure 8c vs Figure 8d) demonstrates that prolonged curing stabilizes the dielectric response of the IPNs, particularly for IPN400, and reduces the dissipation factor of both IPN230 and IPN400. In contrast, the effect of the curing mode on the dielectric characteristics of neat EP networks is considerably smaller (see Figure S12), indicating that incorporation of the DA network increases the temperature sensitivity of the dielectric response, even below 120 °C. For IPN2000, the influence of the curing mode on tan δ_d_ is less pronounced because of its generally higher dielectric losses. Nevertheless, the corresponding permittivity maps (Figure S11) reveal noticeable differences within selected frequency–temperature regions, suggesting that this system deserves further investigation.

#### 3.5.1 Dielectric Behavior at the Industrial Frequency (50 Hz)

For practical electrical insulation applications, dielectric performance at the industrial frequency of 50 Hz is of particular importance. The corresponding tan δ_d_ values at selected temperatures are presented in Figure 9, while the associated relative permittivity values are shown in Figure S13. The dielectric characteristics of IPN400-a2 and IPN400-b2 are omitted because they differ only marginally from the remaining IPN400 formulations; the numerical values are provided in Table S1.

**Figure 9:**
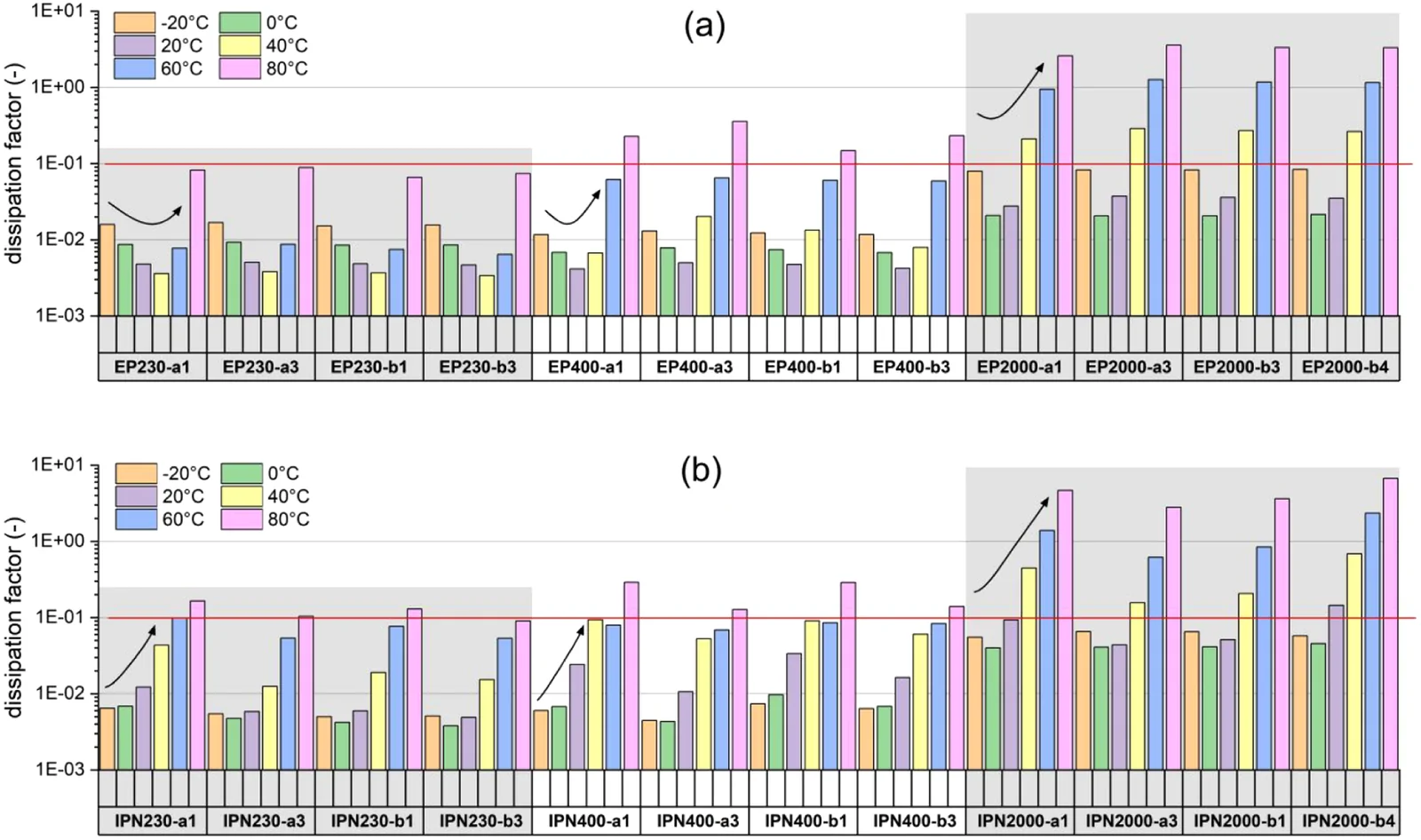
Comparative diagrams of dissipation factors for (a) EP and (b) IPN at a frequency of 50 Hz (IPN variants IPN400-a2 and IPN400-b2 are not included).

At 50 Hz, incorporation of the reversible DA network generally increases the dissipation factor, particularly at elevated temperatures. Nevertheless, the increase relative to the corresponding EP networks remains moderate. A notable difference between the permanent EP and IPN systems is the temperature dependence of tan δ_d_. The EP networks exhibit a decrease followed by an increase in tan δ_d_ between −20 and 80 °C, whereas the IPNs generally show a monotonic increase over the same temperature interval. This change in behavior reflects the interaction between dielectric polarization processes associated with the permanent EP and reversible DA subnetworks. The temperature dependence of tan δ_d_ is governed primarily by the proximity of the glass transition.

To sum up, the dielectric analysis demonstrates that IPNs based on EP2000, containing the longest PPO spacers, exhibit the least favorable electrical insulating properties. In contrast, the IPN400 series provides a considerably better balance between dielectric performance and reversible network functionality, while also showing a clear dependence on the curing protocol. Among the investigated materials, IPN400-a3 exhibited the lowest dielectric losses and relative permittivity, closely followed by IPN400-b3. At room temperature, both materials displayed tan δ_d_ values characteristic of good electrical insulators together with relative permittivity values below 4. Although tan δ_d_ increased with temperature, it remained below the practical limit of 0.1 even at approximately 80 °C (red line in Figure 9). Operation at higher temperatures is not desirable, however, because irreversible cross-linking of the reversible DA network becomes increasingly likely.

### 3.6 Self-Healing Capability

The most important functional property of the prepared interpenetrating polymer networks (IPNs) is their thermally activated self-healing capability, which originates from the reversible Diels–Alder cross-links within the DA subnetwork. The dominant contribution of these reversible cross-links to the rubbery modulus was demonstrated by the thermomechanical analyses discussed above. The self-healing experiments presented below (Figure 10) provide direct qualitative and quantitative evidence of the healing performance of the IPNs, namely of the formulations IPN2000-b4 and IPN400-b2, demonstrating recovery of the tensile properties approaching those of the intact materials (Figure 10a,b).

**Figure 10:**
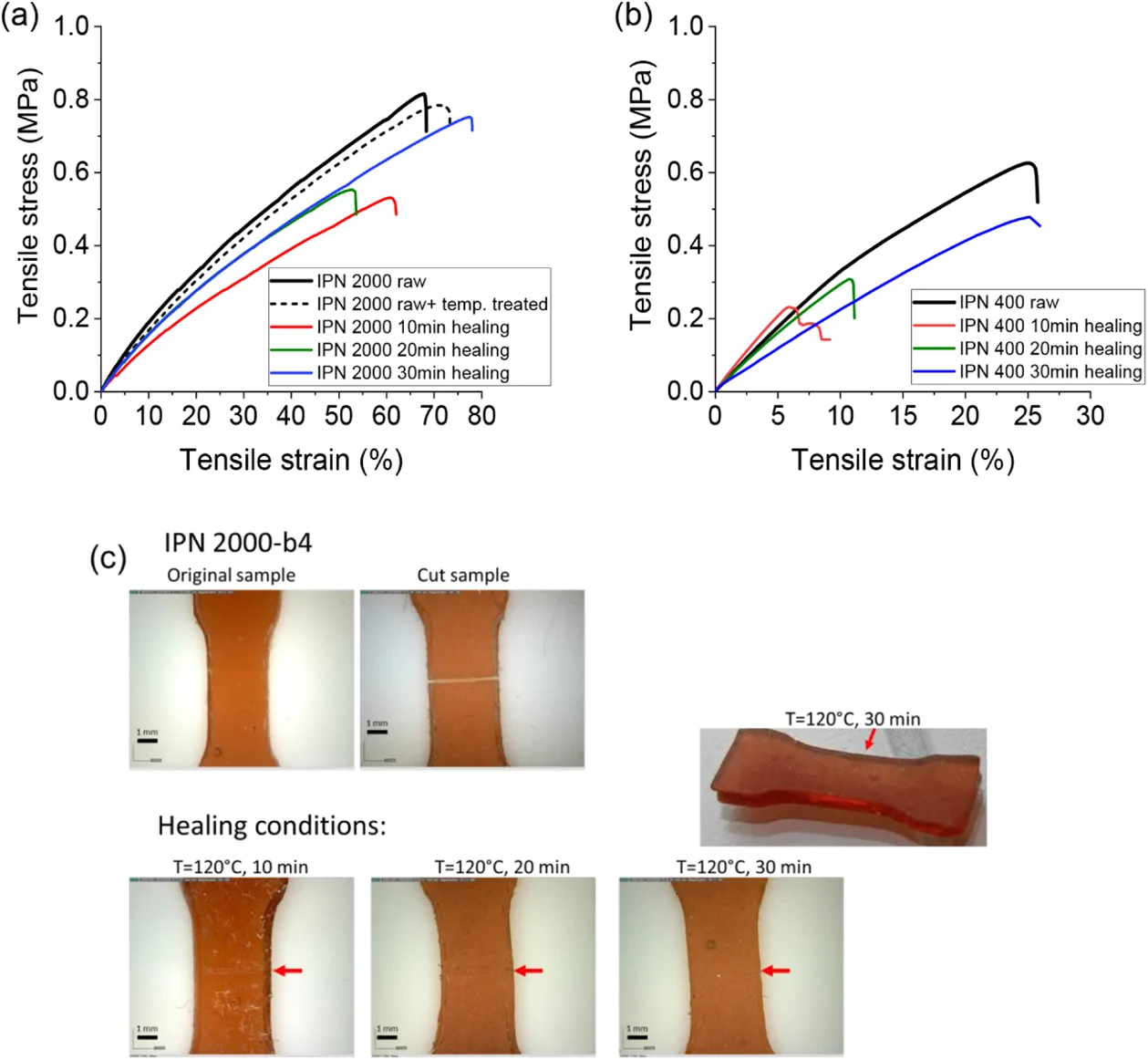
Self-healing: (a) tensile curves of intact and of disrupted and subsequently self-healed samples of the system IPN2000-b4 after different healing times at 120 °C, compared with the intact reference sample (black line), and with the intact reference annealed for 30 min at 120 °C (black dotted line); the tensile curves were recorded at room temperature, where IPN2000 is rubbery; (b) tensile curves of IPN400-b2: intact specimen (black line) and reconnected self-healed samples of IPN400 after healing at 140 °C for 10, 20, and 30 min (red, green, and blue lines, respectively); the tensile curves were recorded at 90 °C, where IPN400 is rubbery; (c) visual observation of the success of self-healing (at 120 °C) of a specimen of IPN2000, which was cut into two.

IPN2000-b4 was selected for the initial self-healing study because its rubbery character at room temperature allows tensile testing without additional heating. Dog-bone specimens were cut into two pieces, reassembled without applying external pressure, and healed at 120 °C (Figure 10c). After 10, 20, or 30 min, the healed specimens were cooled to room temperature and tested in tension (Figure 10a). Lower healing temperatures were also evaluated; however, healing at 110 °C remained significantly less efficient within the investigated 30 min period and was therefore not studied further.

The tensile curves (see Figure 10a; corresponding mechanical parameters are summarized in Figure S14) show that already after 10 min at 120 °C, substantial although incomplete recovery was achieved. Both the extensibility and the tensile modulus remained lower than those of the intact material. After 20 min, the modulus partially recovered, whereas the extensibility remained essentially unchanged or decreased slightly. After 30 min of healing, the extensibility exceeded that of the original intact specimen, whereas the tensile modulus remained only slightly lower. This behavior was reproduced in several independent experiments. If the healing efficiency (*HE*) is calculated as

HE=100%(tensile_strength_healed/tensile_strength_intact⁡⁢)

then its value can be given as 65.1, 67.8, and 92.2% after 10, 20, and 30 min of healing, respectively (using the data from Figure 10a).

The increase in extensibility after 30 min of healing (approximately 14% higher than that of the original material), accompanied by a slight decrease in modulus, suggests partial structural reorganization of the network during annealing. This interpretation was confirmed by subjecting an intact specimen to the same thermal treatment (120 °C for 30 min) without previous mechanical damage. As shown in Figure 10a, annealing alone produced a similar increase in extensibility together with a slight reduction in modulus, indicating a moderate increase in the effective mesh size of the reversible DA network and, consequently, a small decrease in the effective cross-link density.

IPN400-b2, identified above as the most promising formulation in terms of thermomechanical, dielectric, and self-healing performance, was also evaluated by tensile testing before and after healing (see Figure 10b; photographs in Figure S15). Because this material is glassy at room temperature, tensile measurements were performed at 90 °C, where the network exhibits rubber-like behavior.

The initially tested healing protocol was identical to that used for IPN2000-b4 but proved insufficient. Owing to its substantially higher cross-link density and shorter elastic PPO segments, IPN400-b2 exhibited poor healing at both 120 and 130 °C. Only heating at 140 °C resulted in satisfactory reconnection of the cut specimens.

Unlike IPN2000-b4, where appreciable healing was achieved after only 10 min, IPN400-b2 required the full 30 min treatment at 140 °C to restore its original extensibility completely (100% in Figure 10b). Healing times of 10 and 20 min produced only limited recovery, characterized by markedly reduced elongation at break. The slower healing kinetics are attributed to the lower segmental mobility associated with the shorter elastic chains and higher cross-link density of the IPN400 network.

The corresponding healing efficiencies (for IPN400-b2) were 36.9, 49.2, and 76.4% after 10, 20, and 30 min, respectively (using data from Figure 10b). In contrast to IPN2000-b4, thermal treatment did not increase the extensibility beyond that of the original material; instead, the initial mechanical response was essentially restored.

Overall, the above tensile experiments demonstrate that the developed IPNs, consisting of reversible DA and permanent EP subnetworks in a 2:1 mass ratio, exhibit rapid and highly efficient thermally activated self-healing at their optimized healing temperatures (120 °C for IPN2000-b4 and 140 °C for IPN400-b2). Remarkably, efficient restoration of the mechanical properties is achieved despite irreversible damage to the permanently cross-linked EP network, which cannot actively participate in the healing process. The following section examines more specialized self-healing scenarios relevant to morphological defects that may develop in electrical insulating layers.

#### 3.6.1 Microcrack Self-Healing

The ability of the developed IPNs to repair mechanically induced microcracks was evaluated using the optimized IPN400-b2 formulation (see Figure 11a,b). Because this material exhibited the best overall combination of thermomechanical, dielectric, and self-healing properties, it was selected for this investigation. The permanently cross-linked EP400 network was used as a reference. Since both materials are glassy at room temperature, microcracks were readily introduced by repeated bending. The damaged specimens were subsequently subjected to the optimized healing treatment identified for IPN400-b2, namely heating at 140 °C for 30 min.

**Figure 11:**
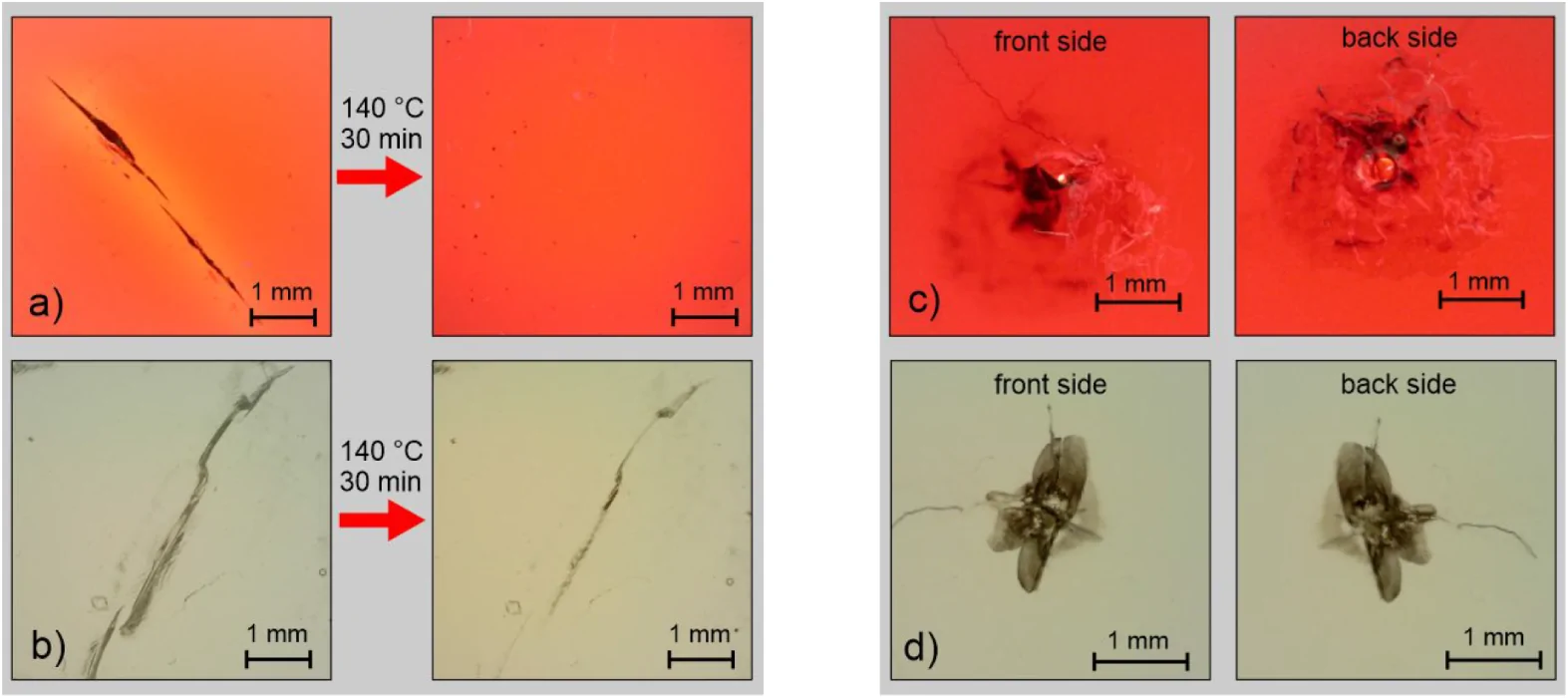
(a, b) healing (at 140 °C for 30 min) of microcracks caused by bending and (c, d) damage caused by dielectric breakdown at 25 kV, as observed by light microscopy: (a, c) the IPN400-b2 system; the orange (up to dark red) color is characteristic of the studied IPN double networks; (b, d) the EP400 system.

As shown in Figure 11a, the well-defined microcrack in IPN400-b2 disappeared completely after thermal treatment, indicating effective crack healing. In contrast, the corresponding crack in the reference EP400 remained clearly visible after the same treatment (Figure 11b), although partial crack narrowing was observed, particularly near its lower end. This effect is attributed to elastic stress relaxation of the permanently cross-linked EP network after heating above *T*_g_, rather than to any intrinsic healing mechanism.

#### 3.6.2 Dielectric Breakdown

The response of IPN400-b2 and the EP400 reference to dielectric breakdown is compared in Figure 11c,d. Although both materials exhibited nearly identical dielectric strength (approximately 20 kV mm^–1^, corresponding to a breakdown voltage of 25 kV for specimens 1.25 mm thick), their failure mechanisms differed markedly.

The reference EP400 displayed extensive cracking, accompanied by pronounced carbonization surrounding the breakdown channel. In contrast, IPN400-b2 exhibited virtually no crack formation in the vicinity of the channel; only localized material deformation was observed, while the level of carbonization was substantially reduced.

These differences are attributed to localized heating generated during an electrical discharge. Whereas the conventional EP network undergoes irreversible thermal degradation (carbonization), the temperature increase in IPN400-b2 is also sufficient to activate the reversible Diels–Alder network. The resulting DA bond exchange is therefore believed to promote (partial) in situ self-healing during the dielectric breakdown event, suppressing crack initiation and propagation while simultaneously reducing the extent of carbonization.

#### 3.6.3 Electrical Treeing and Self-Healing (Prebreakdown Damage)

The susceptibility of the IPN networks to electrical stress-induced damage and their ability to recover through thermally activated self-healing were investigated using electrical treeing experiments followed by a thermal healing treatment. The IPN2000 network was selected for these studies because compared with IPN400, it exhibits a higher tendency toward degradation under prolonged electrical stress, particularly through the formation of large electrical trees. This behavior is attributed to the higher dielectric loss of IPN2000, as discussed in Section 3.4.

The treeing damage in IPN2000 and its subsequent thermal healing are presented in Figure 12, together with the behavior of the chemically analogous, nonself-healing epoxy network EP2000, which serves as a reference. Electrical treeing (see, e.g., refs 42,43 ) is a progressive degradation phenomenon characterized by the formation and propagation of branched conductive channels within the bulk of an electrically insulating material. In polymers, the process is initiated by localized partial discharges at microscopic defects, or in microregions of enhanced electric field concentration. These discharges cause progressive degradation of the polymer matrix, while the formation of volatile gaseous degradation products further accelerates tree propagation. Under continued electrical stress, the tree eventually grows to span the entire thickness of the insulating layer, thus resulting in dielectric breakdown and a complete loss of insulation performance. The growth rate, morphology, and branching density of the electrical tree are governed by the intrinsic properties of the polymer, the characteristics of the applied electric field (particularly the applied voltage), and the geometry of the tree initiation site, including the needle tip diameter and cone angle.

**Figure 12:**
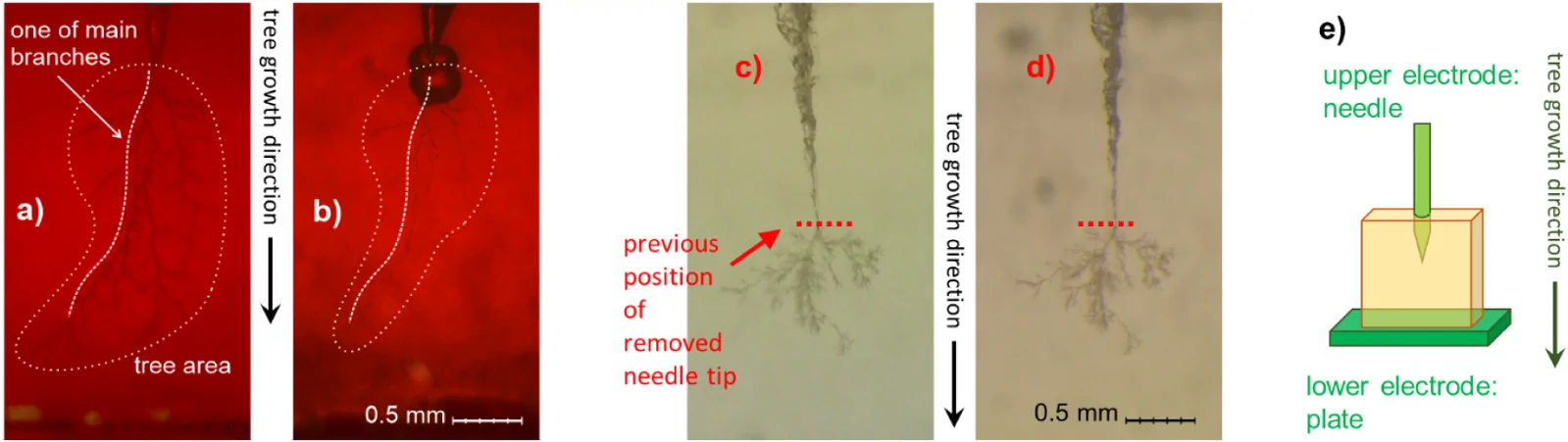
Graphical overview of the electrical treeing and subsequent thermal self-healing experiments performed on IPN2000 and on the EP2000 reference system: a) optical micrograph of an electrical tree generated in an IPN2000 specimen; b) the same specimen imaged from the identical viewing direction after thermal self-healing; in both images, the needle electrode remained embedded in the specimen; c) optical micrograph of an EP2000 reference specimen after electrical treeing; d) the same EP2000 specimen after exposure to the identical thermal treatment like the one applied to IPN2000; in the case of the EP2000 specimens, the needle electrode was removed prior to imaging, just after terminating the tree growth; e) schematic illustration of the experimental electrical treeing setup.

Figure 12a presents an exemplary electrical tree generated in an IPN2000 specimen (4 mm thick) under an applied voltage of 9.5 kV, recorded by transmitted-light optical microscopy. The same specimen was subsequently subjected to the optimized thermal self-healing protocol for this composition (120 °C for 30 min). The regenerated specimen, observed from the same viewing direction, is shown in Figure 12b. A pronounced reduction in the extent of the electrical tree is evident, with several of the primary tree branches becoming discontinuous after the healing treatment. Additionally, a newly formed gas bubble can be noted in the vicinity of the needle tip, which remained embedded in the specimen. The bubble was most likely generated by the thermal expansion of gaseous pyrolysis products, previously generated during electrical tree formation.

In contrast, EP2000, the permanently cross-linked reference system (expectedly) exhibited no detectable recovery of the treeing damage: As demonstrated by Figure 12c vs d, the morphology of the electrical tree remained completely unchanged after exposure to the same thermal treatment that effectively promoted healing in the IPN2000 system.

## 4 Conclusions

Advanced self-healing interpenetrating epoxy networks (IPNs) were successfully developed by combining a permanent epoxy–amine (EP) subnetwork with a reversible Diels–Alder (DA)-based one. The DA subnetwork was formed through maleimide–furan cycloaddition, and its dynamic DA chemistry provided the thermally triggered self-healing capability. The EP network, on the other hand, ensured structural (shape) stability during the healing process.

Optimally cured and healed IPNs exhibited near-complete recovery of mechanical properties after damage.

The dual IPN demonstrated efficient microcrack repair, partial suppression of degradation caused by dielectric breakdown, as well as recovery from electrical treeing damage, thus highlighting their potential as multifunctional self-healing dielectric materials.

The influence of the length of flexible poly­(propylene oxide) (PPO) segments (230, 400, and 2000 g mol^–1^) on the thermal, mechanical, and self-healing properties of the IPNs was systematically investigated. Increasing PPO molecular weight reduced the glass transition temperature and rubbery-state modulus, moderately decreased the DA dissociation temperature, and improved the self-healing efficiency. This confirms the key role of chain flexibility in controlling network dynamics.

In contrast, dielectric analysis revealed the best insulating properties for systems containing the shortest PPO segments.

The IPN based on PPO chains with a molecular weight of 400 g mol^–1^ provided the most balanced combination of mechanical performance, self-healing ability, and dielectric properties, thus representing the most promising candidate for self-healing electrical insulation applications.

The synthesis and healing protocols were optimized to preserve DA reversibility and minimize undesired side reactions, such as Michael addition and radical polymerization of maleimide units. The most effective IPN network formation was achieved using a two-step curing procedure involving an initial high-temperature stage (120 °C, DA network dissociated), followed by prolonged curing at a lower temperature (90 °C). Under optimized conditions, efficient self-healing was achieved after only 10 to 30 min at 120 °C with IPN2000 and after 30 min at 140 °C with IPN400.

The preservation of dynamic DA chemistry after a healing treatment was confirmed by thermomechanical analysis and DSC measurements, including cyclic experiments.

## Supplementary Material



## Notes

DMTA: Thermomechanical properties of EP permanent networks, DA reversible networks, and IPN networks; yclic DMTA tests; DSC: Thermal transitions of the studied materials; Dielectric properties of the IPN, EP, and DA networks; self-healing: Tensile parameters of intact and self-healed IPN2000; Figure S1: EP permanent networks: DMTA: Curing mode effect on the profiles of EP230, EP400, and EP2000: temperature dependence of (a, c, e) storage shear modulus *G′* and (b, d, f) mechanical loss factor tandm; Figure S2: DA reversible networks: (a–d) DMTA profiles of the DA230 and DA2000 systems: Temperature dependence of (a, c) storage shear modulus *G′* and (b, d) mechanical loss factor tan δ_m_; (e) overlay of the *G*′ = f­(*T*) curves (including hysteresis) for all the DA systems: DA230, DA400, and DA2000; (f) trendlines for the comparison of the DA networks, which describe trends of *T*_g_, *T*_diss_, and also of the well-visible hysteresis (as Δ*T*) of both temperatures in heating/cooling cycles; the *x*-axis of (a, b) is zoomed: in the remaining diagrams, the *T*-region of (a, b) extends to the left of the added vertical lines; Figure S3: Interpenetrating networks IPN230 and IPN2000: DMTA: Curing mode effect and comparison with related EP and DA networks as references: Temperature dependence of (a, c) storage shear modulus *G′* and (b, d) mechanical loss factor tandm; Figure S4: IPN230, IPN400, and IPN2000 networks: DMTA: Detailed overview of all tested curing modes, and comparison with related EP and DA networks as references: Temperature dependence of (a, c, e) storage shear modulus *G′* and (b, d, f) mechanical loss factor tandm; Figure S5: Cyclic DMTA tests: Successive cooling/heating runs with (a) the neat reversible network DA230-a1 between 145 and 70 °C, (b) DA400-a1 between 150 and 50 °C and (c) the interpenetrating network IPN2000-b4 between 25 (or 30) and 150 °C; the series of experiments started from the molten state as the “1st run” in the case of (a) and (b), or from the “cold” solid state in the case of (c) which was heated up to 150 °C during the “1st run”; depicted are the temperature-dependent curves of the storage modulus *G′* (top) and of the loss factor tandm (bottom); Figure S6: Repeated DSC tests with the reversible network DA400-a1: (a) repeated scans with a maximum temperature of 150 °C; (b) repeated scans up to the lower temperature of 120 °C; cooling traces are omitted for clarity; Figure S7: Self-healing treatment (30 min at 140 °C) effect on DSC traces of: (a) the interpenetrating network IPN400-b2, (b) the reversible network DA400-a1: comparison of DSC traces (heating scan) of freshly prepared “intact” specimens (respective bottom line in a, b) with the trace of a sample which endured 30 min at 140 °C (respective top line in a, b); integral areas of DA dissociation, as well as of the exothermic side reactions are estimated with the help of dotted lines; the temperature at which self-healing is carried out (140 °C) is highlighted by vertical red dotted lines (these always are found deep in the DA-dissociation region; the *y*-axis zoom in (a) and (b) is different, but in both graphs the major ticks correspond to 0.2 W/g, (the minor ones to 0.1 W/g); Figure S8: Thermal history effect in IPN and DA systems: Sets of DSC traces for (a) the interpenetrating network IPN400-b2 and (b) the reversible network DA400-a1; DSC traces were measured as heating scans up to 250 °C; the specimens’ thermal history consisted in (1) no previous heating (“intact specimen”), or in previous heating up to (2) 120 °C, (3) 130 °C, (4) 140 °C, and (5) 150 °C; cooling traces are omitted for clarity, except for the system (1) where the cooling trace (1c) is shown; Figure S9: DSC traces of the permanent network EP400: Repeated scans with maximum temperature of 120 or 250 °C; Figure S10: Relative permittivity: frequency–temperature dependencies for (a) EP, (b) DA, (c) IPN at the shortest curing mode a1, and (d) IPN at the longest curing mode b3 or b4; Figure S11: Dielectric constant: frequency–temperature dependencies for EP and IPN cured at different temperature programs—influence of curing temperature and time; Figure S12: Dissipation factor: frequency–temperature dependencies for EP and IPN cured at different temperature programs—influence of curing temperature and time; Figure S13: Comparative diagrams of dielectric constant for (a) EP and (2) IPN at a frequency of 50 Hz (IPN variants IPN400-a2 and IPN400-b2 are not included); Figure S14: Trends of individual tensile properties of the IPN2000 system, obtained for intact, or for disrupted and subsequently self-healed specimens after different healing times at 120 °C; one mechanically intact specimen (label: “raw + T”) also was subjected to annealing at 120 °C for 30 min for comparative reasons; the different specimen types are aligned on the *x*-axis; the compared properties are (a) Young’s moduli, (b) stress at break (strength), (c) strain at break (extensibility), and (d) toughness; Figure S15; visual observation of the success of self-healing (at 140 °C) of an IPN400 specimen, which was cut into two; Table S1: Summary of dielectric constant and dissipation factor values for selected temperatures above 0 °C for all tested samples (PDF)
