# Supplementary material for: Interpenetrating Dual Epoxy Networks with Reversible Covalent Bonds: Shape-Constant Self-Healing Electrically Insulating Materials
Source: ACS Appl Polym Mater. 2026 Aug 20;8(16):13537–55. doi: 10.1021/acsapm.6c01094 (PMC13532041; doi:10.1021/acsapm.6c01094)
Supplement: Supplementary file 1 [file ap6c01094_si_001.pdf]

Supporting Information

Interpenetrating Dual Epoxy Networks with Reversible Covalent Bonds: Shape-Constant Self-Healing Electrical Insulating Materials

Beata Strachota[1)], Adam Strachota[1)*], Petr Kadlec[2)], Radek Polanský[2)], Todor Batakliev[3,4)]

[1]*Institute of Macromolecular Chemistry, Academy of Sciences of the Czech Republic, Heyrovského nám. 2, 162 00 Praha 6, Czech Republic.*

[2]*Department of Materials and Technology, Faculty of Electrical Engineering, University of West Bohemia, Univerzitní 2795/26, 301 00 Plzeň, Czech Republic.*

[3]*Institute of Mechanics, Bulgarian Academy of Sciences, Acad. G. Bonchev Str., Block 4, 1113 Sofia, Bulgaria.*

[4]*Center of Competence for Mechatronics and Clean Technologies "Mechatronics, Innovation, Robotics, Automation and Clean Technologies" - MIRACle, 1113 Sofia, Bulgaria.*

*Corresponding author: E-mail address: strachota@imc.cas.cz

1 DMTA – Thermo-mechanical properties

1.1 EP permanent networks

EP230

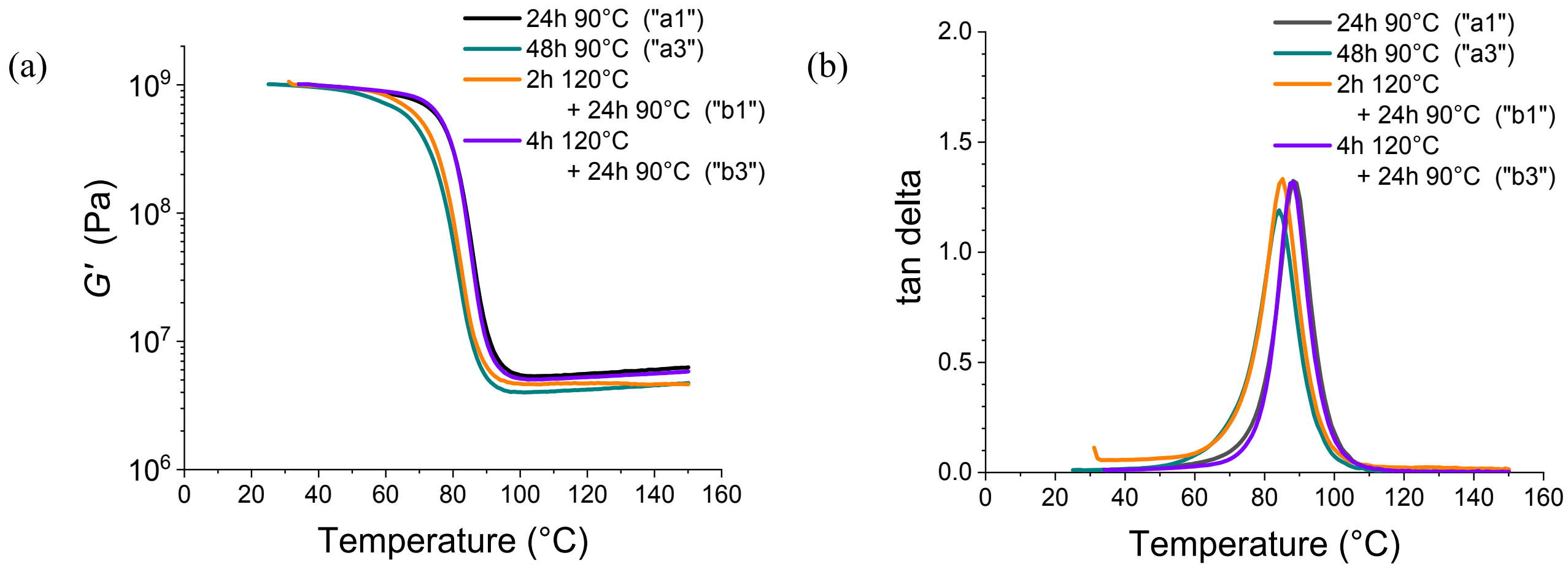

EP400

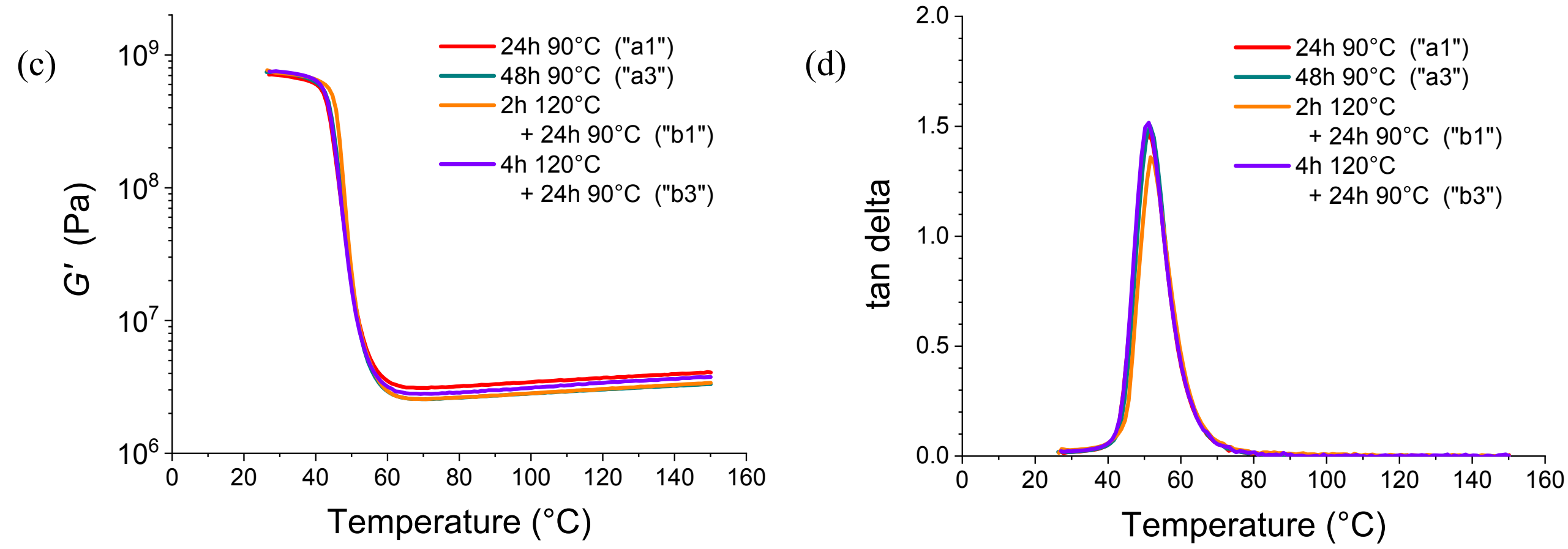

EP2000

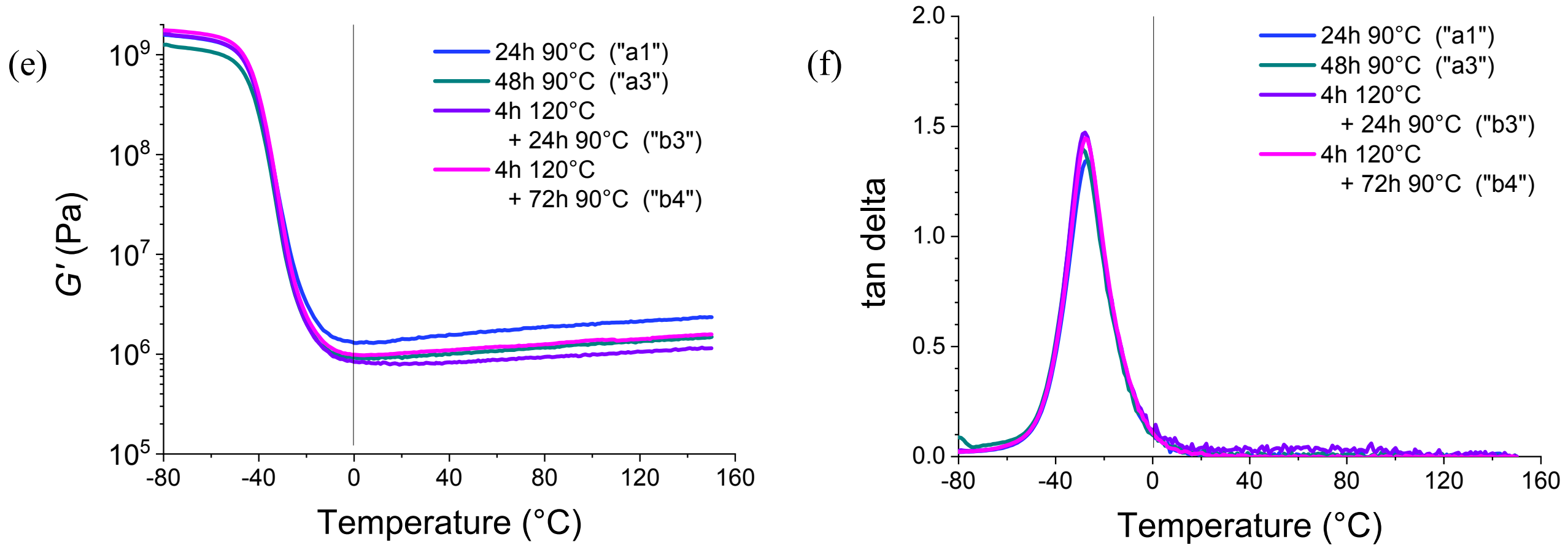

Fig. S 1. *EP permanent networks: DMTA: Curing Mode effect on the profiles of EP230, EP400, and EP2000: temperature-dependence of (a, c, e) storage shear modulus G' and (b, d, f) mechanical loss factor* $\tan\delta_m$.

The results of thermo-mechanical analysis via DMTA of the simple systems based only on permanent EP (epoxide-amine) network, namely EP230, EP400 and EP2000, are presented in ***Fig. 4***a, b in the main manuscript. The curves of the shear modulus and of the loss factor (DMTA profiles) clearly illustrate one distinct glass transition region (a step in G' and a simple peak in tan δ). Moreover, at a heating/cooling rate of 3°C/min, the DMTA profiles show no visible hysteresis when recorded in either heating or cooling runs.

The values of the glass transition temperature (T_g) and the moduli in the high-temperature (rubbery) region are controlled by the molecular structures of the α, ω-diamine hardeners D230, D400, and D2000. These are similar (see ***Fig. 1***, main manuscript), but the lengths of their linear chains are different (ca. tetramer, ca. octamer, and ca. 33-mer, respectively). With increasing molecular chain length and thus molecular mass, the T_g value decreases, due to a higher volume fraction of the flexible polypropylene oxide chains from the Diamines. The value of the rubbery modulus is also lowered by the longer chains, because with longer chains (which act as elastic springs in the network), the number of these chains per volume unit (concentration of elastically active chains = 'crosslinking density') decreases.

It was noted that the curing mode "a1" (just 90 °C for 24 hours) is sufficient to achieve nearly perfect cure of all tested EP networks, as demonstrated in ***Fig. S 1***. Only the system EP230 was found to be significantly sensitive to the choice of curing mode (small but visible shifts in the glass transition temperature T_g), due to its high reactivity (high molar concentrations of both types of functional groups, because both monomers are small molecules). Nevertheless, even in the case of EP230, the effect of the chosen curing mode is small. In the case of the IPN networks discussed further below, however, their growth is more difficult, and the different curing modes result in distinctly different products.

1.2 DA reversible networks

DA230-a1

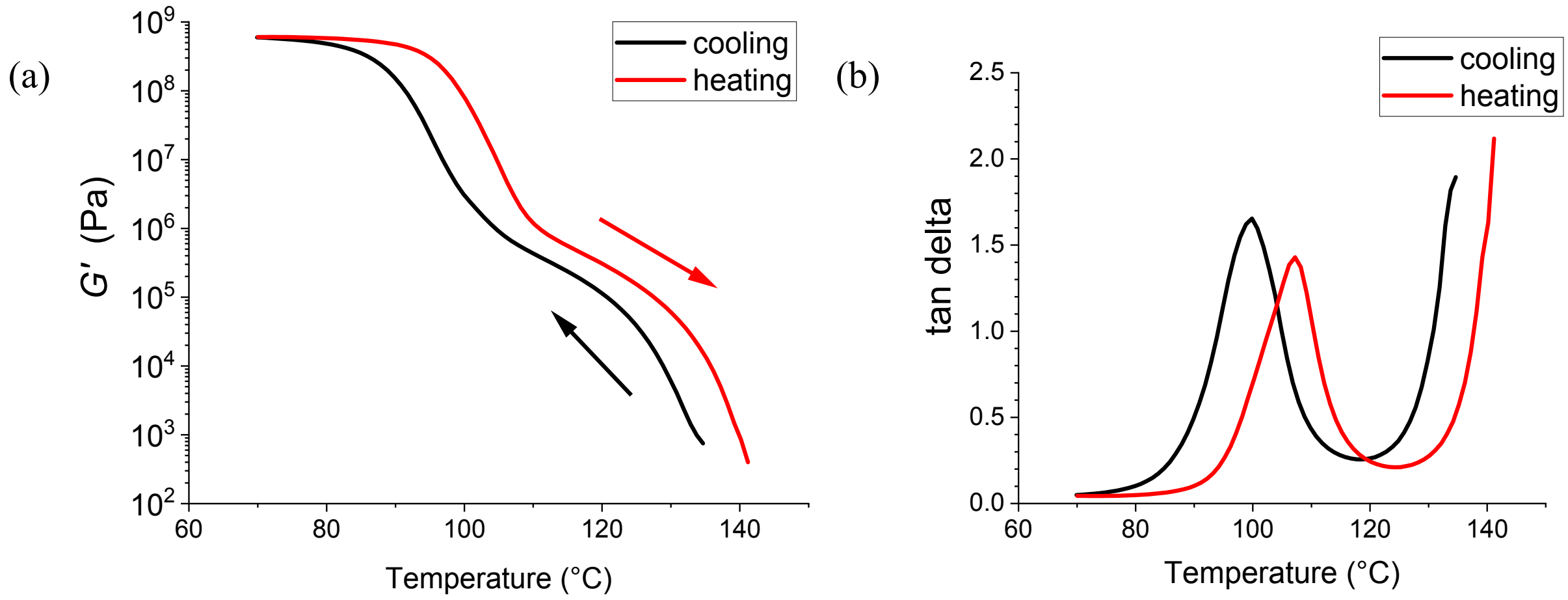

DA2000-a1

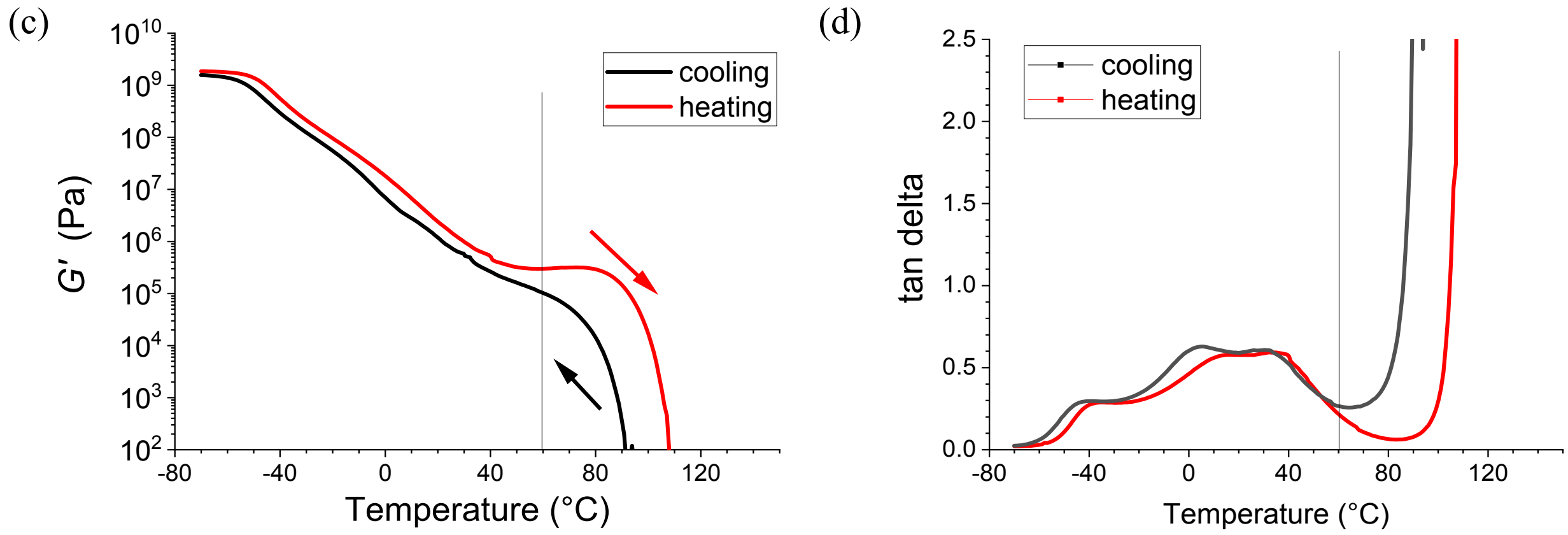

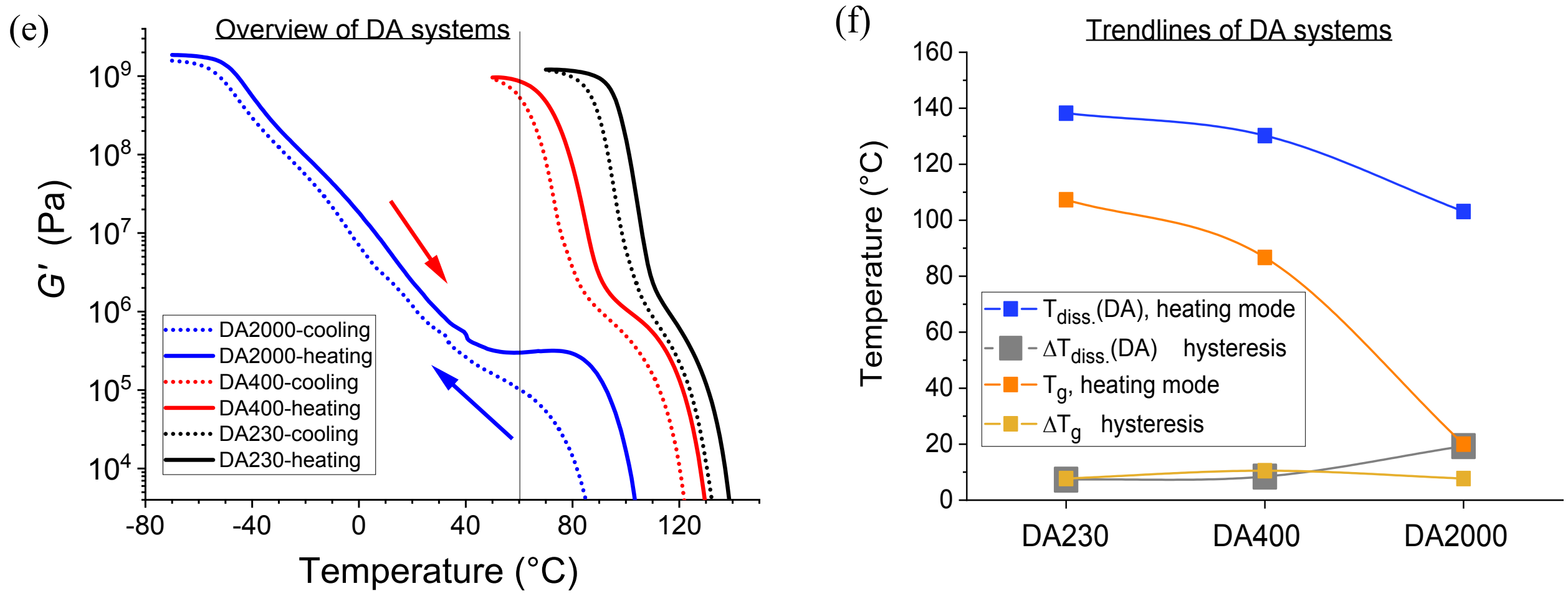

Fig. S 2. *DA reversible networks: (a–d) DMTA profiles of the DA230 and DA2000 systems: Temperature-dependence of (a, c) storage shear modulus G' and (b, d) mechanical loss factor* $\tan\delta_m$*; (e) overlay of the G' = f(T) curves (including hysteresis) for all the DA systems: DA230, DA400, and DA2000; (f) trendlines for the comparison of the DA networks, which describe trends of* T_g*,* $T_{diss.}$ *and also of the well-visible hysteresis (as* ΔT*) of both temperatures in heating/cooling cycles; the x-axis of (a, b) is zoomed: in the remaining diagrams, the T-region of (a, b) extends to the left from the added vertical lines.*

The crosslinking in DA is more sophisticated than the epoxy-amine reaction, which yields EP. The kinetics and the reversibility of the cure of DA networks similar to the ones studied in this work, at varied temperatures, was recently studied by the authors in [36]: It was found, that the DA cure slowly proceeds already at room temperature (cure time: 33.3 h), while at 70°C it is moderately fast (cure time: 3.33 h). At 90°C, the process is even faster (1.67 h needed), but also partly reversible and dynamic (equilibrium conversion lower than 100%), while above 120°C, the dissociation increasingly prevails. The temperature of 90°C (mode "a1") was hence chosen as the standard cure temperature for the neat DA networks, also because it was high enough to exceed the T_g of the stiffest system, DA230.

In the case of **DA230** (see ***Fig. S 2*** a, b), the disconnection reaction (retro-Diels-Alder, 'melting step') becomes dramatically dominant near 135°C, while upon cooling, the reconnection reaction (Diels-Alder) becomes very rapid near 127°C, at an 8°C lower temperature. Also, the glass transition is 'delayed' by 10°C in the case of the cooling experiment ('hysteresis'). Both the observed effects can be attributed to the kinetics of retro-DA/DA. The heating and cooling experiments were carried out at a rate of 3°C/min, which, normally, for simple polymers, does not generate any well-visible hysteresis when comparing cooling- and heating scans (e.g., in the case of permanent EP networks).

In the case of DA400, an analogous behaviour is observed, like for the above-discussed system, with characteristic melting/solidification (retro-DA / DA) temperatures of ca. 130°C and 123°C (see ***Fig. 4***c, d in the main manuscript).

In the case of **DA2000** (***Fig. S 2*** c, d), the retro-DA / DA temperatures are markedly smaller: 104°C and 85°C, respectively.

If the heating and cooling runs, as in ***Fig. 4***c and d, and ***Fig. S 2***, are cyclically repeated several times, similar curves are obtained, as in the first heating/cooling scans, although both characteristic temperatures slightly shift to higher values (see ***Fig. S 5***a, b). This might be attributed to gradual network ordering. The network ordering and its kinetics might also explain why hysteresis is observed not only for the melting transition but also for T_g. The wide hysteresis and the significantly lowered melting point in DA2000 could be attributed to the dilution of the functional groups and to the high segmental mobility in this system. Generally, it can also be noted that after mixing the components of the pure DA systems and after one heating run to 140°C, the DMTA profiles, as shown in ***Fig. 4c***, d and ***Fig. S 2***, do not depend on the previous cure history (curing mode).

---

[36] Strachota, B.; Morand, A.; Dybal, J.; Matějka, L. Control of gelation and properties of reversible Diels–Alder networks: Design of a Self-Healing Network. *Polymers* **2019**, *11* (6), 930. https://doi.org/10.3390/polym11060930.

1.3 IPN networks

IPN230

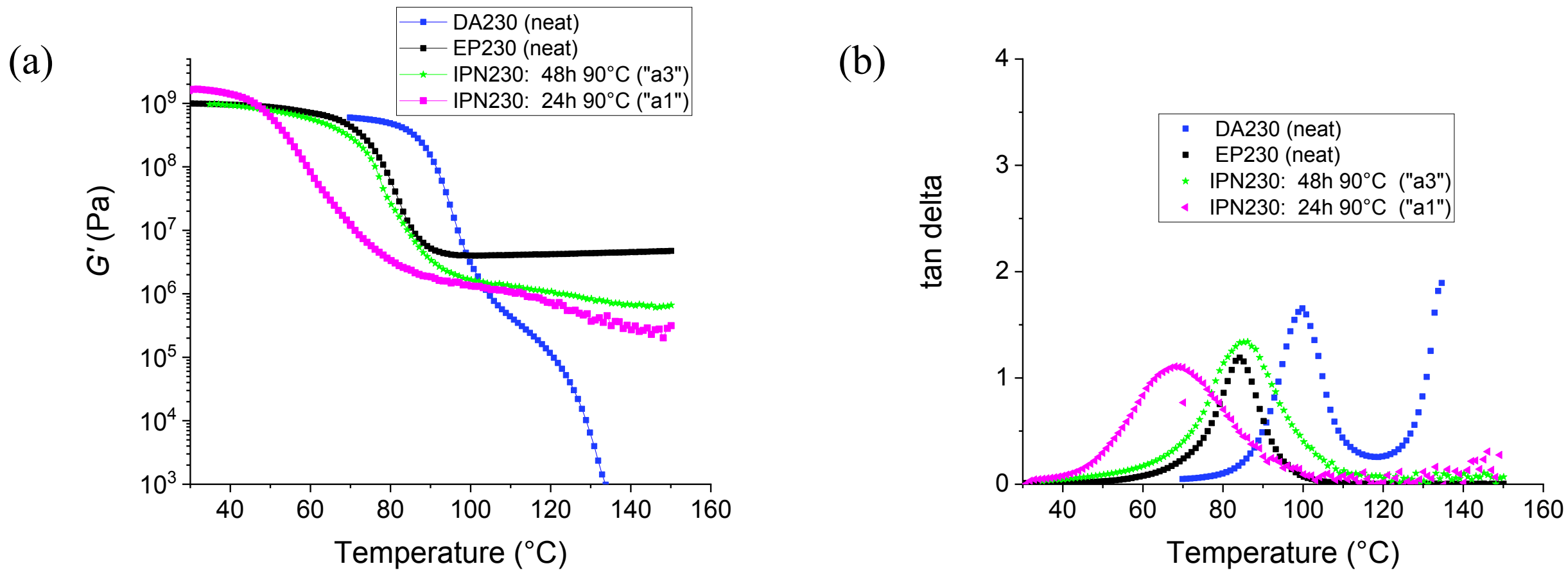

IPN2000

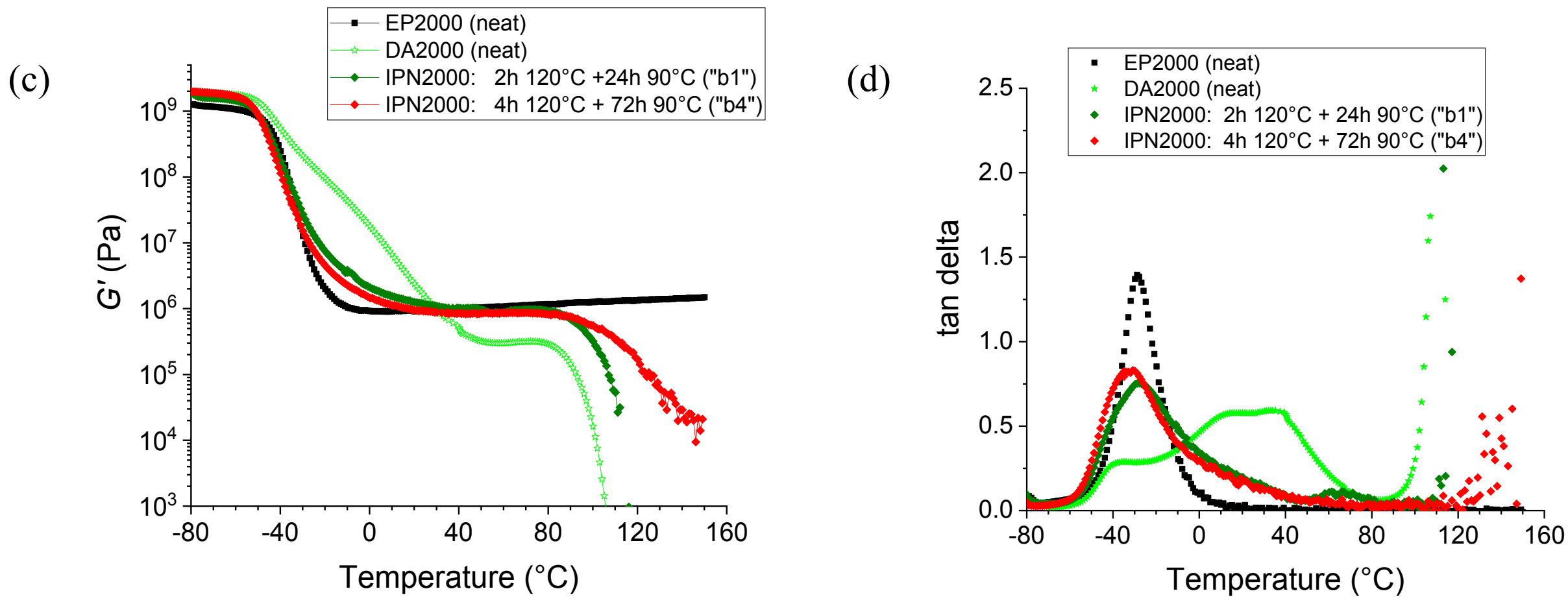

Fig. S 3. *Interpenetrating networks IPN230 and IPN2000: DMTA: Curing mode effect and comparison with related EP and DA networks as references: Temperature-dependence of (a, c) storage shear modulus G' and (b, d) mechanical loss factor tan*δ_m.

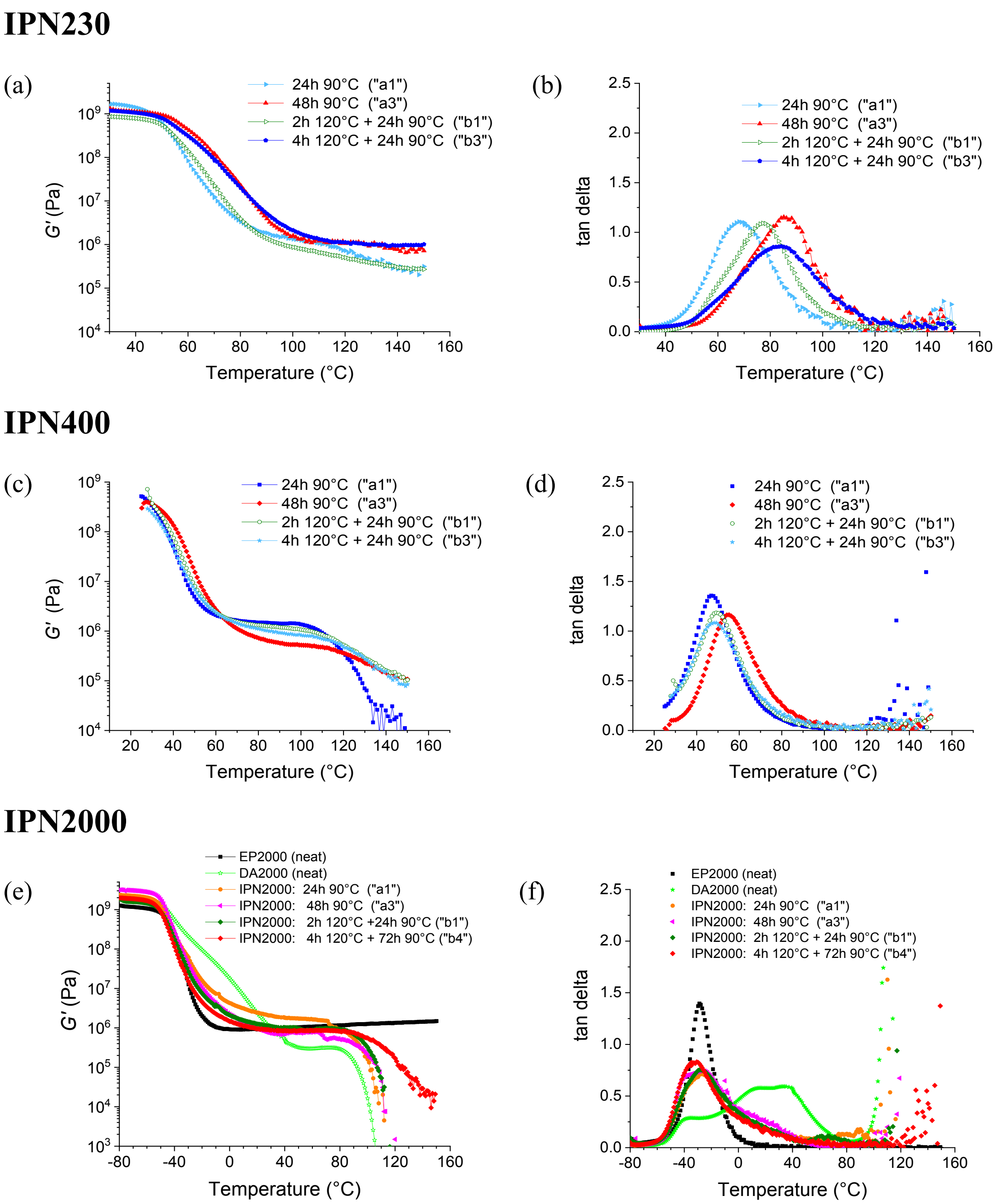

Fig. S 4. *IPN230, IPN400, and IPN2000 networks: DMTA: Detailed overview of all tested curing modes, and comparison with related EP and DA networks as references: Temperature-dependence of (a, c, e) storage shear modulus G' and (b, d, f) mechanical loss factor tanδ$_m$.*

In the case of the cure of IPN networks, several effects play an important role:

(1) The (macro)monomers of the permanent EP sub-network are strongly diluted by the components of the dominant DA sub-network (ratio of EP and DA weights is 1:2), which results in a significantly slower cure of the EP sub-network.

(2) The DA sub-network is also diluted by EP (macro)monomers and reacts more slowly.

(3) The further-above-discussed side reactions of the DA monomers (see section 3.1 Curing mode optimisation), especially of BMI, lead to permanent crosslinking instead of a reversible one, and to covalent inter-connection of both networks. The occurrence of these reactions is favoured by high temperatures and by longer exposure to them.

IPN230 (see ***Fig. S 3***a,b and ***Fig. S 4***a,b) subjected to the mildest curing mode "a1" (24h at 90°C) yields a moderately promising IPN product (***Fig. S 3***a,b), albeit the retro-DA step above 100°C, which indicates potential self-healing ability, is only moderate. Prominent irreversible crosslinking of the DA component via the undesired Michael addition can be suspected. Already, the somewhat more intense curing mode "a3" (48h at 90°C) leads to a nearly complete suppression of the retro-DA step (***Fig. S 4***a,b), thus practically preventing self-healing. The milder high-temperature mode "b1" (2h at 120°C + 24h at 90°C) yields a similarly crosslinked product like in case of "a3" (see ***Fig. S 4***a,b), while the high-temperature mode "b3" achieves a complete covalent crosslinking of the DA system in IPN230-b3 (see ***Fig. S 4***a,b).

All the IPN230 systems display a reduced crosslinking density (lower G') in the rubbery region in comparison to the neat system EP230. This difference is the strongest (ca. 1.5 orders) in the case of the curing mode "a1". Some IPN230 systems display a markedly lower glass transition temperature than the neat EP system EP230 (T_g = 103°C). This is especially the case of IPN230-a1 (T_g = 68°C) and IPN230-b1 (T_g = 77°C), while the drop in T_g is markedly smaller in the case of IPN230-a3 and IPN230-b3 (T_g = 86 and 84°C, respectively). These differences in the T_g values of the IPN230 products could be attributed to more or less disordered network structures, with longer curing times favouring more ordered structures and higher T_g. The variation in T_g values is much smaller in the more flexible IPN systems, IPN400 and IPN2000 (see below).

The system IPN230 proved to be very challenging due to the high reactivity of its components.

IPN400 (see ***Fig. 5*** in the main Manuscript and ***Fig. S 4***c,d), as illustrated by its DMTA profiles, displayed a less dramatic reactivity, a smaller tendency to side reactions, and hence more promise as a self-healing shape-constant material than IPN230. The curing mode "a1" yielded a product which completely melted at ca. 130°C due to incomplete cure of the EP component (see ***Fig. S 4***c,d), while the remaining curing modes "a3", "b1", and "b3" yielded products with a significant but somewhat flattened retro-DA step near 130°C, which was more distinct than in case of IPN230-a1. Further tuning led to curing modes "a2" (34h at 90°C) and "b2" (4h at 120°C + 16h at 90°C), which produced non-melting and strongly self-healing IPN400 products with a large retro-DA step (see ***Fig. 5***). Among these optimized products, the system IPN400-b2 proved to be the better one, because IPN400-a2 tended to some flow during self-healing of disrupted samples at 140°C, in contrast to IPN400-b2 (see inlay photos in ***Fig. 5***).

IPN2000 was prepared as a model material, which is rubbery at room temperature. It displayed a further decrease in reactivity compared to the cure of IPN230 and IPN400, which

can be attributed to the dilution of functional groups in the reaction mixture. Consequently, products cured using the modes “a1”, “a3”, and “b3” were all displaying complete melting near 110°C, at the temperature of the retro-DA dissociation of the DA component network (see ***Fig. S 3*** c, d and ***Fig. S 4*** e, f), due to insufficient cure of the EP component, which was not attractive for self-healing applications. Consequently, the harsher curing mode “b4” (4h at 120°C + 72h at 90°C) was applied and ultimately yielded an infusible product, IPN2000-b4, which simultaneously displayed a distinct retro-DA step above 120°C.

All the IPN2000 systems displayed very similar DMTA profiles, like the EP system EP2000, except for the additional distinct retro-DA step in the IPN products.

1.4 Cyclic DMTA tests

DA230-a1

(a)

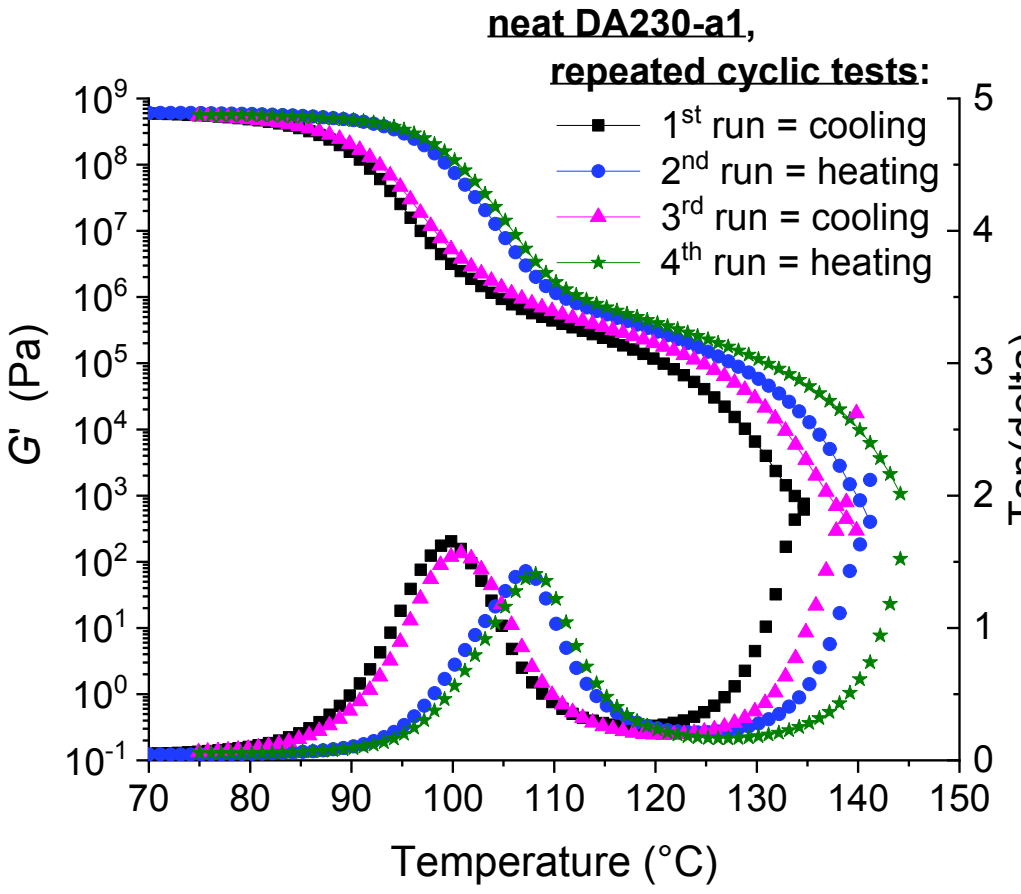

DA400-a1

(b)

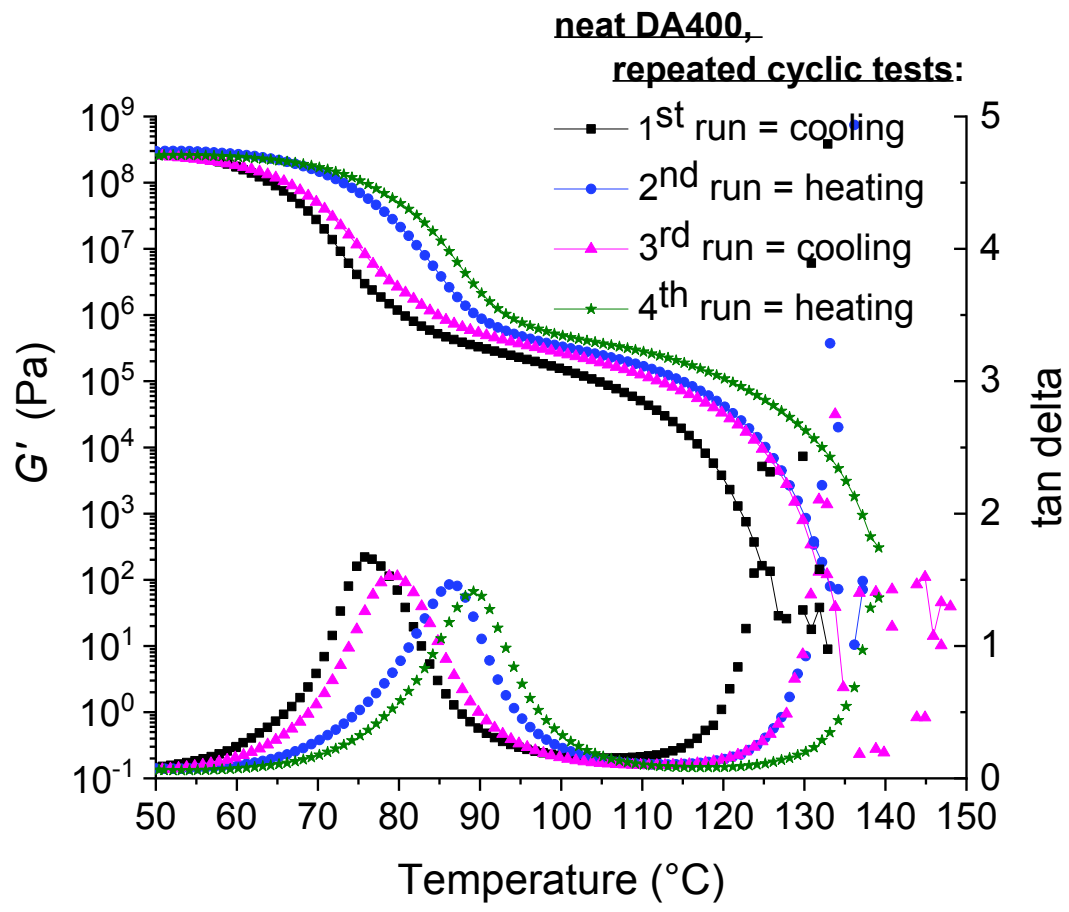

IPN2000

(c)

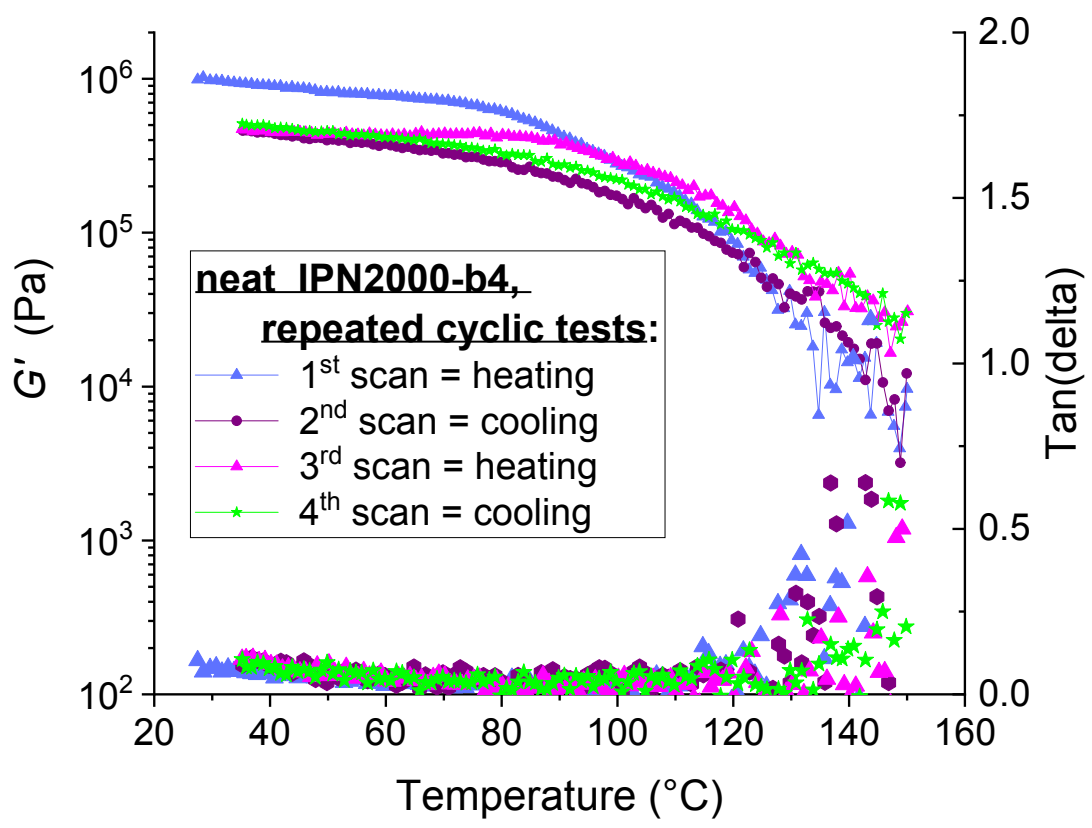

Fig. S 5. *Cyclic DMTA tests: Successive cooling/heating runs with (a) the neat reversible network DA230-a1 between 145 and 70°C, (b) DA400-a1 between 150 and 50°C and (c) the interpenetrating network IPN2000-b4 between 25 (or 30) and 150°C; the series of experiments started from the molten state as the "1st run" in case of (a) and (b), or from the 'cold' solid state in case of (c) which was heated up to 150°C during the "1st run"; depicted are the temperature-dependent curves of the storage modulus G' (top) and of the loss factor* $\tan\delta_m$ *(bottom).*

2 DSC – Thermal transitions

(a)

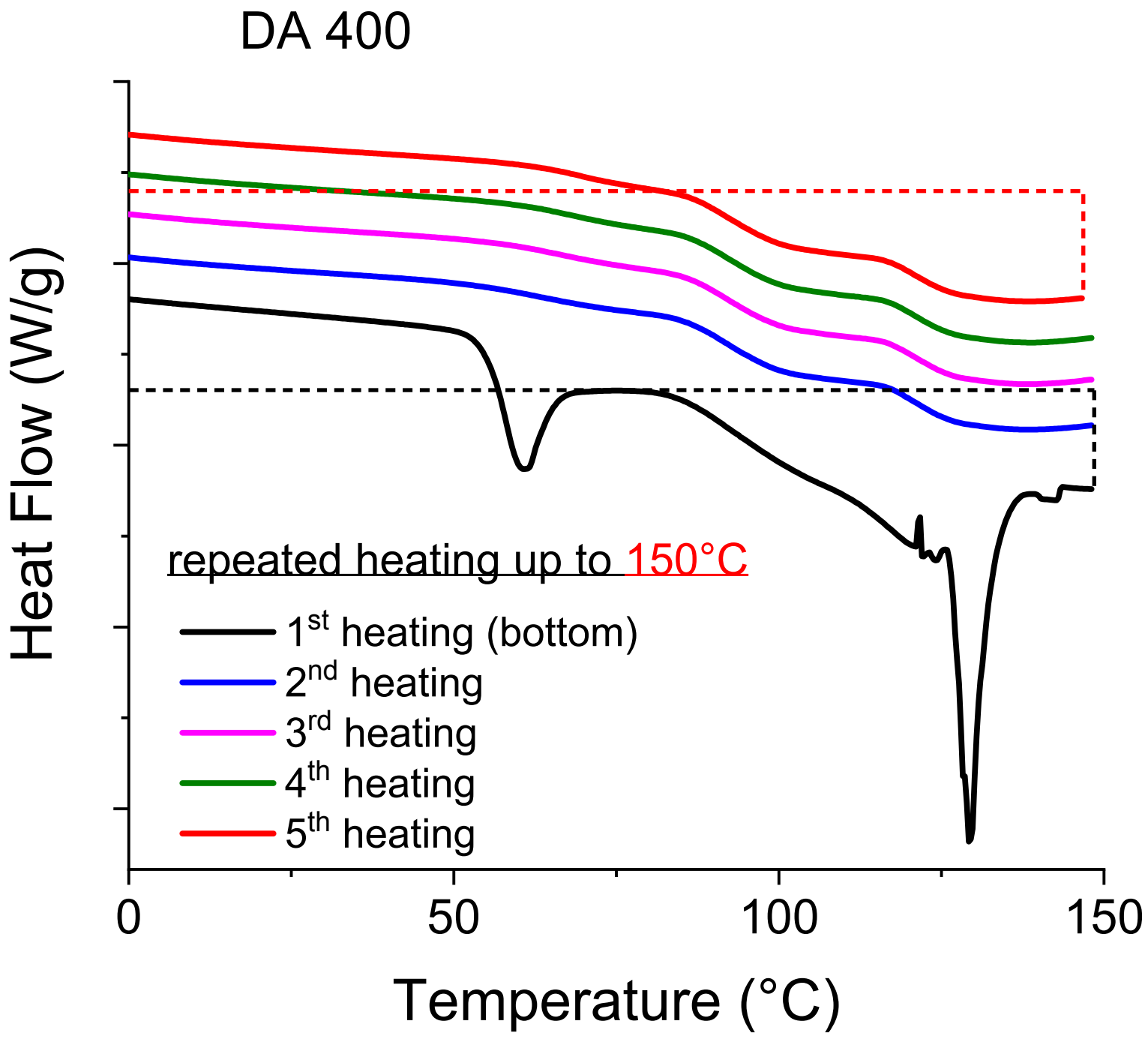

(b)

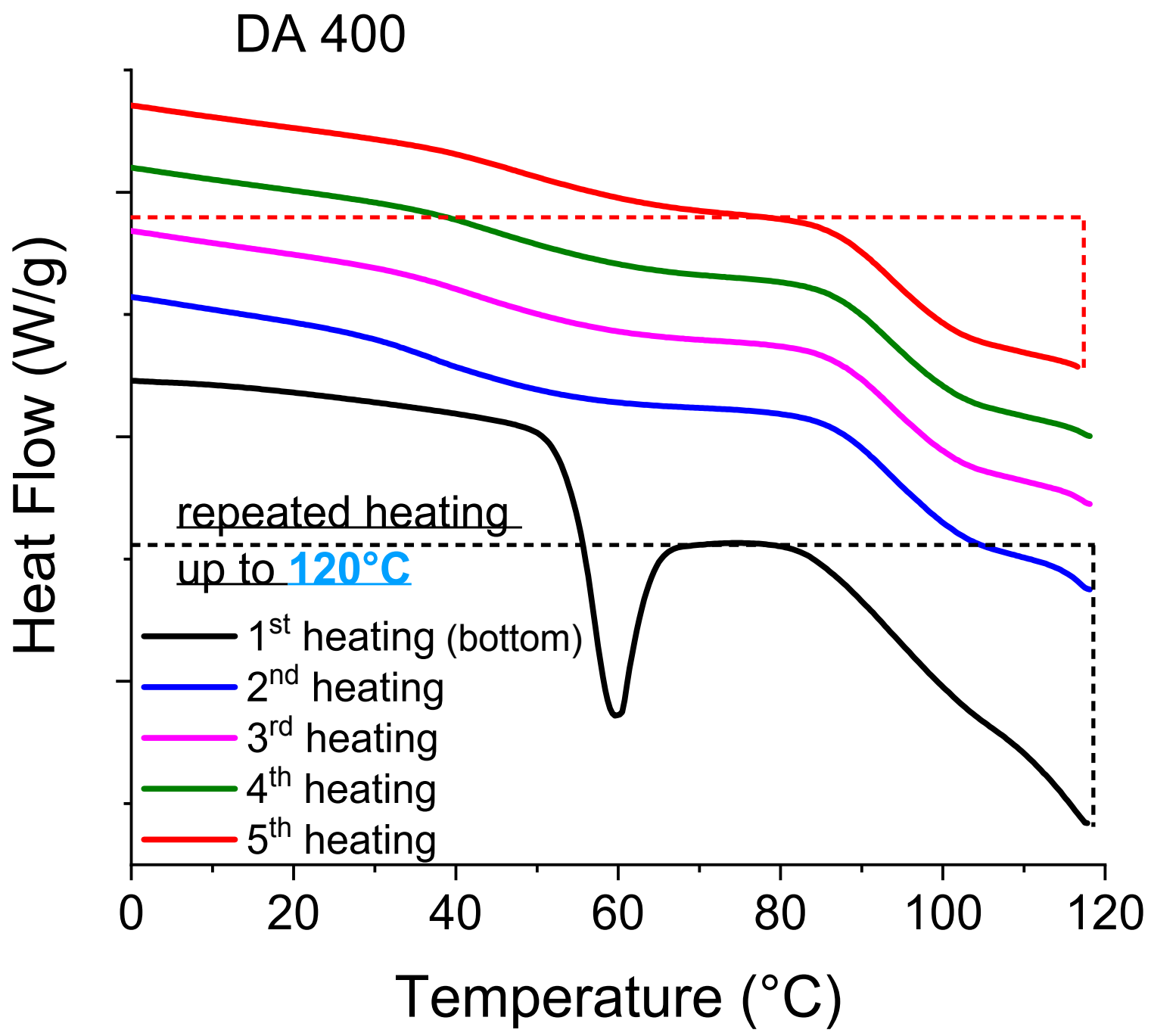

Fig. S 6. *Repeated DSC tests with the reversible network DA400-a1: (a) repeated scans with maximum temperature of 150°C; (b) repeated scans up to the lower temperature of 120°C; cooling traces are omitted for clarity.*

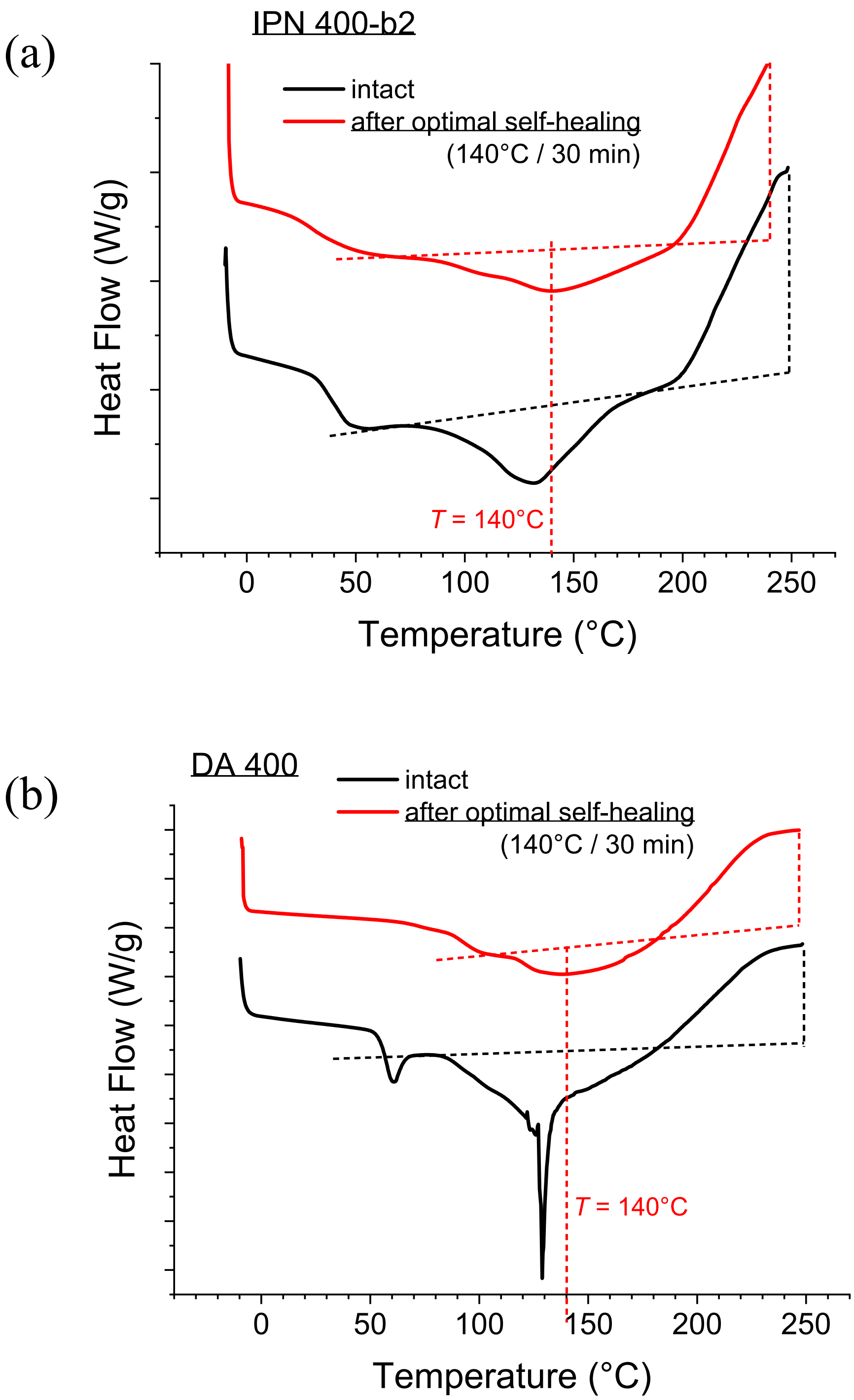

Fig. S 7. *Self-healing treatment (30 min at 140°C) effect on DSC traces of: (a) the interpenetrating network IPN400-b2, (b) the reversible network DA400-a1: comparison of DSC traces (heating scan) of freshly prepared 'intact' specimens (respective bottom line in a, b) with the trace of a sample which endured 30 min at 140°C (respective top line in a, b); integral areas of DA-dissociation, as well as of the exothermic side-reactions are estimated with the help of dotted lines; the temperature at which self-healing is carried out (140°C) is highlighted by vertical red dotted lines (these always are found deep in the DA-dissociation region; the Y-axis-zoom in (a) and (b) is different, but in both graphs the major ticks correspond to 0.2 W/g, (the minor ones to 0.1 W/g).*

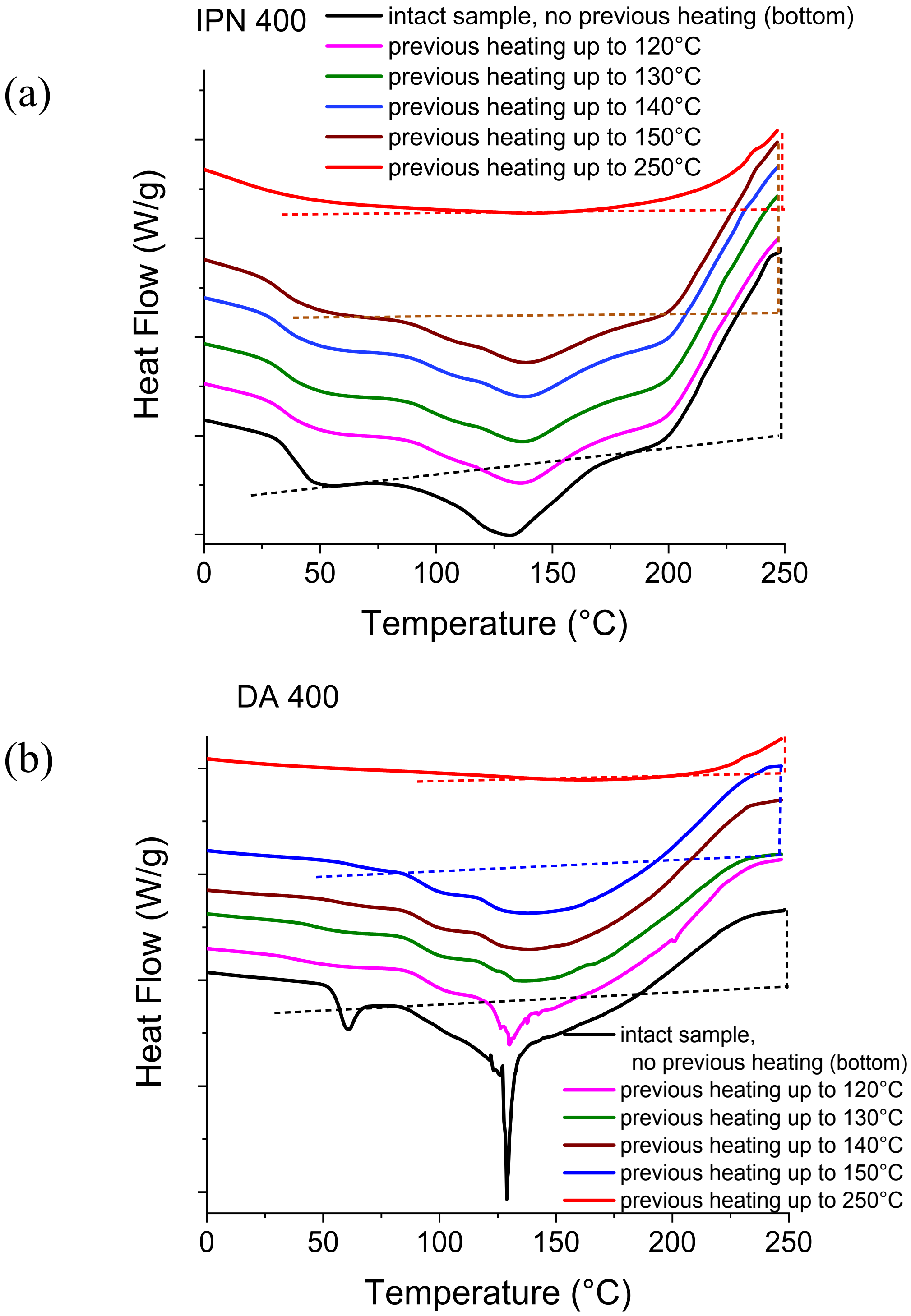

Fig. S 8. *Thermal history effect in IPN and DA systems: Sets of DSC traces for (a) the interpenetrating network IPN400-b2 and (b) the reversible network DA400-a1; DSC traces were measured as heating scans up to 250°C; The specimens' thermal history consisted in: (1) no previous heating ('intact specimen'), or in previous heating up to: (2) 120°C, (3) 130°C, (4) 140°C, and (5) 150°C; cooling traces are omitted for clarity, except for the system (1) where the cooling trace (1c) is shown.*

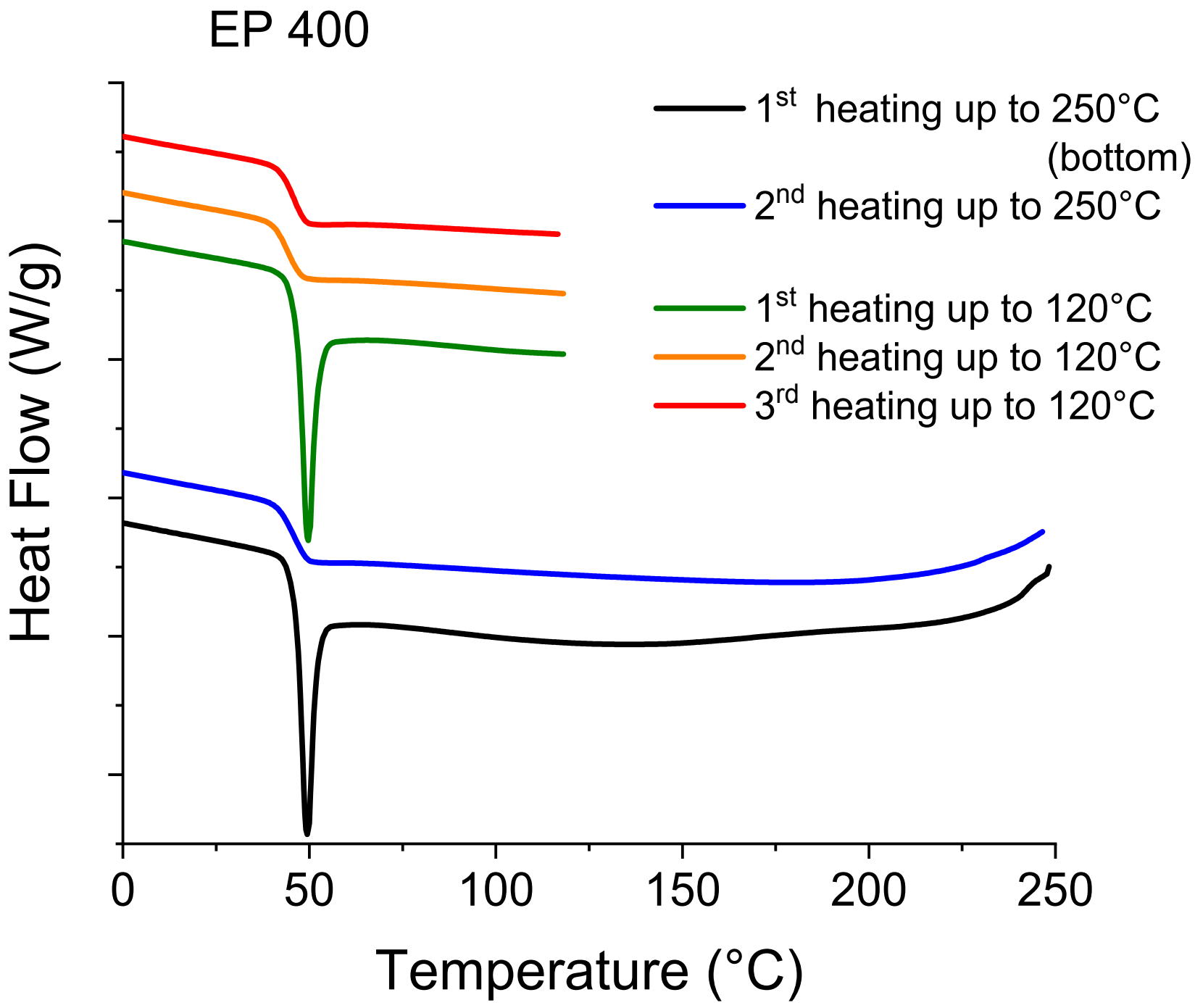

Fig. S 9. *DSC traces of the permanent network EP400: Repeated scans with maximum temperature of 120°C or 250°C.*

3 Dielectric properties of the IPN, EP and DA networks

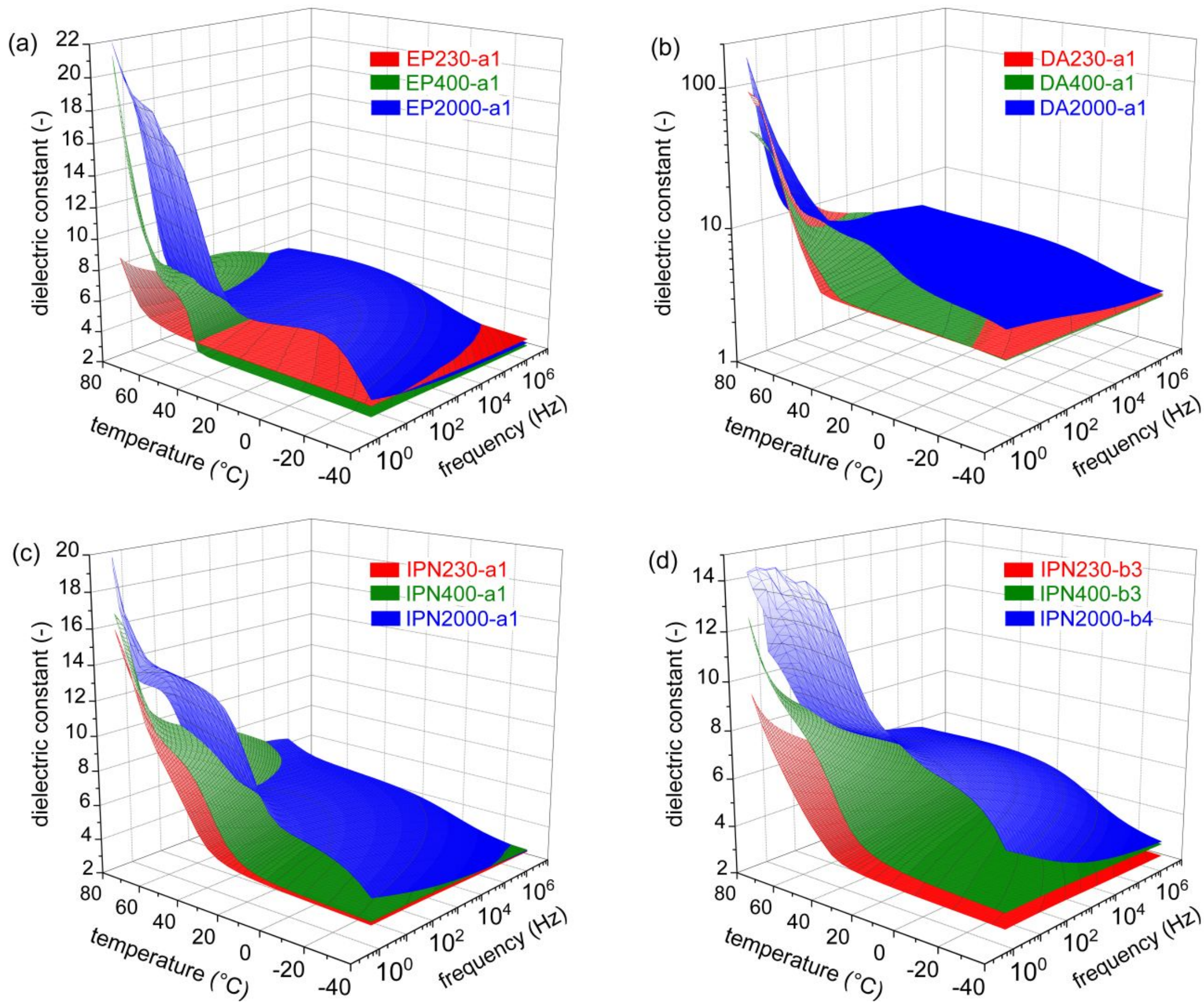

Fig. S 10. *Relative permittivity: frequency-temperature dependencies for (a) EP, (b) DA, (c) IPN at the shortest curing mode a1, and (d) IPN at the longest curing mode b3 or b4.*

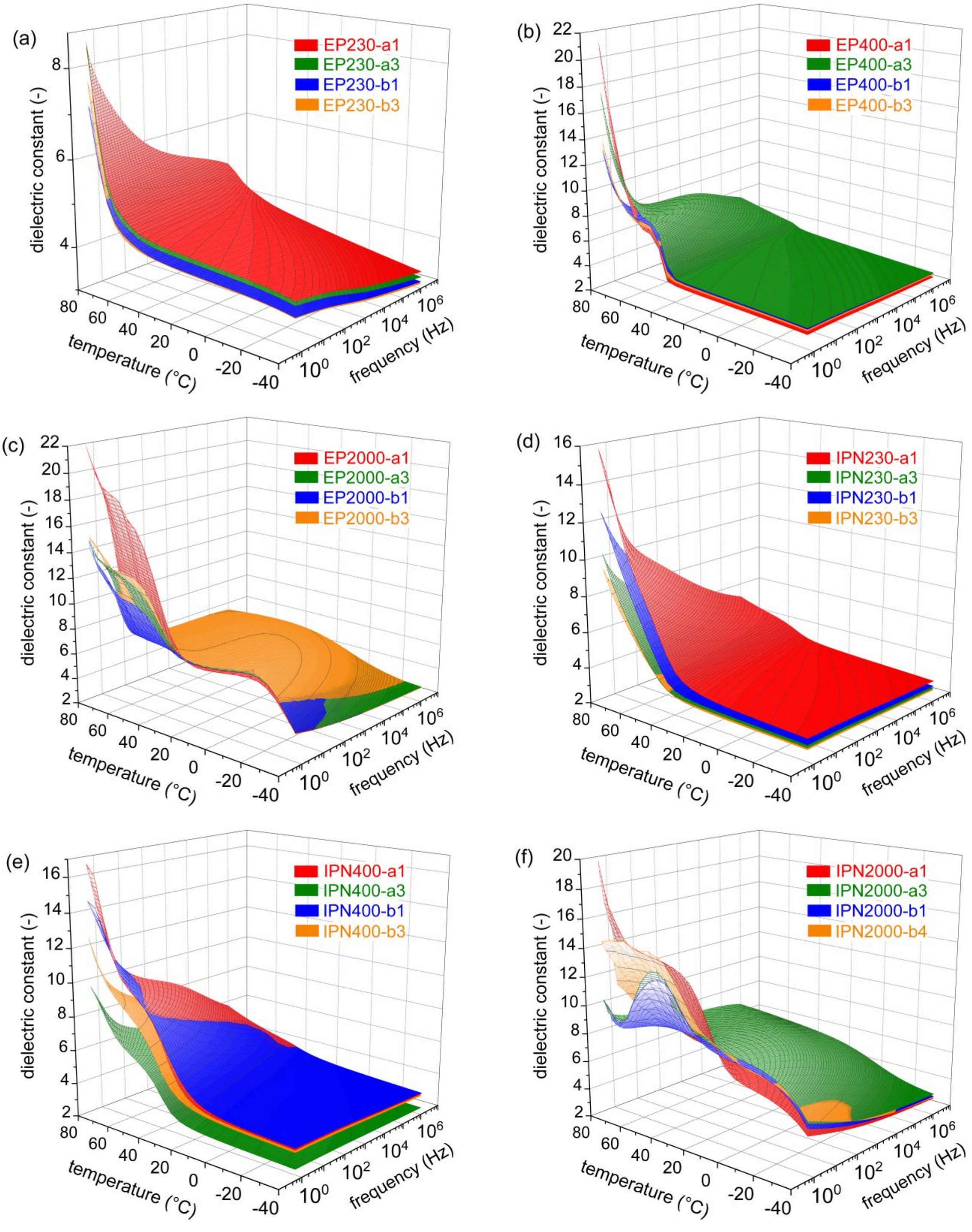

Fig. S 11. *Dielectric constant: frequency-temperature dependencies for EP and IPN cured at different temperature programs – influence of curing temperature and time.*

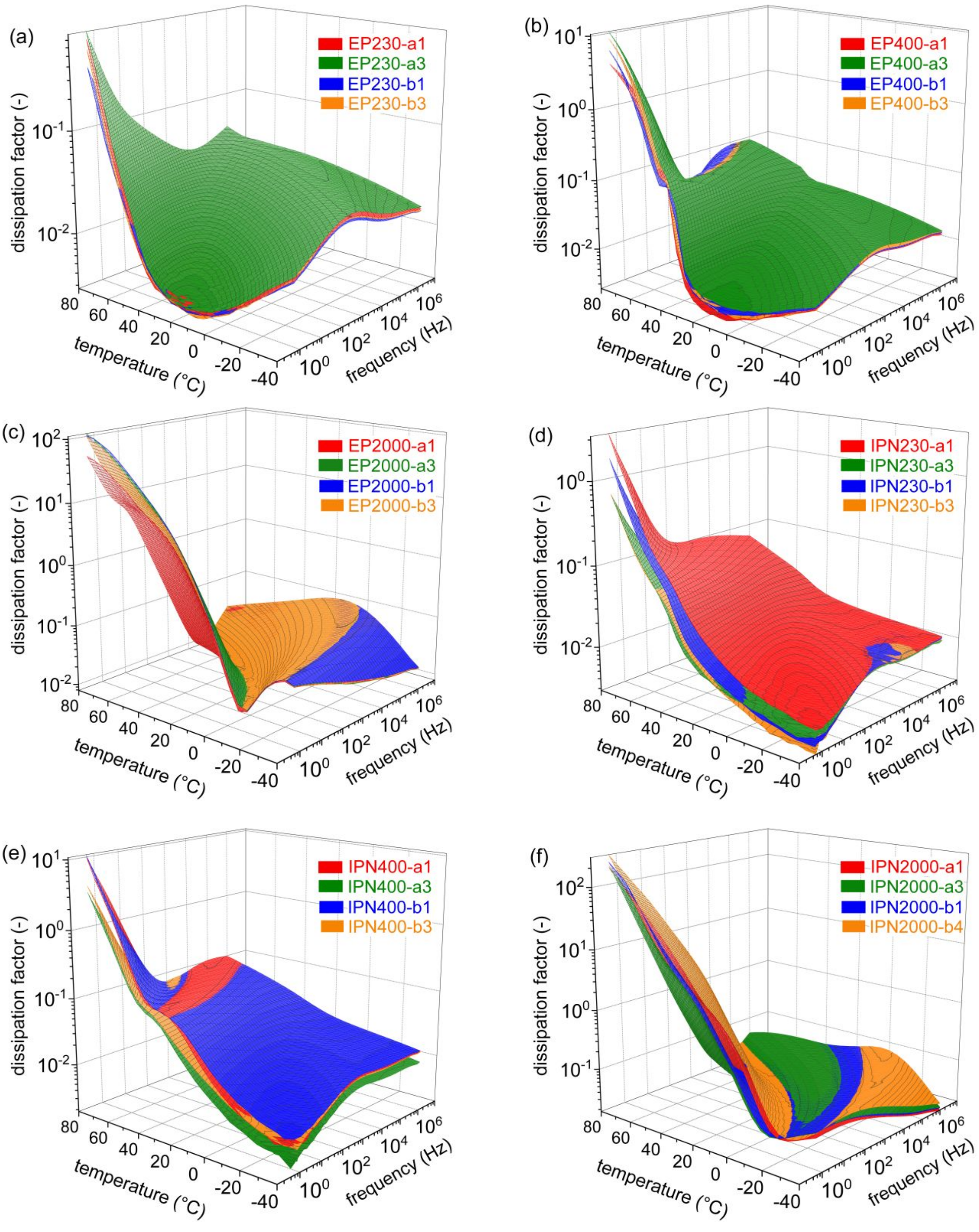

Fig. S 12. *Dissipation factor: frequency-temperature dependencies for EP and IPN cured at different temperature programs – influence of curing temperature and time.*

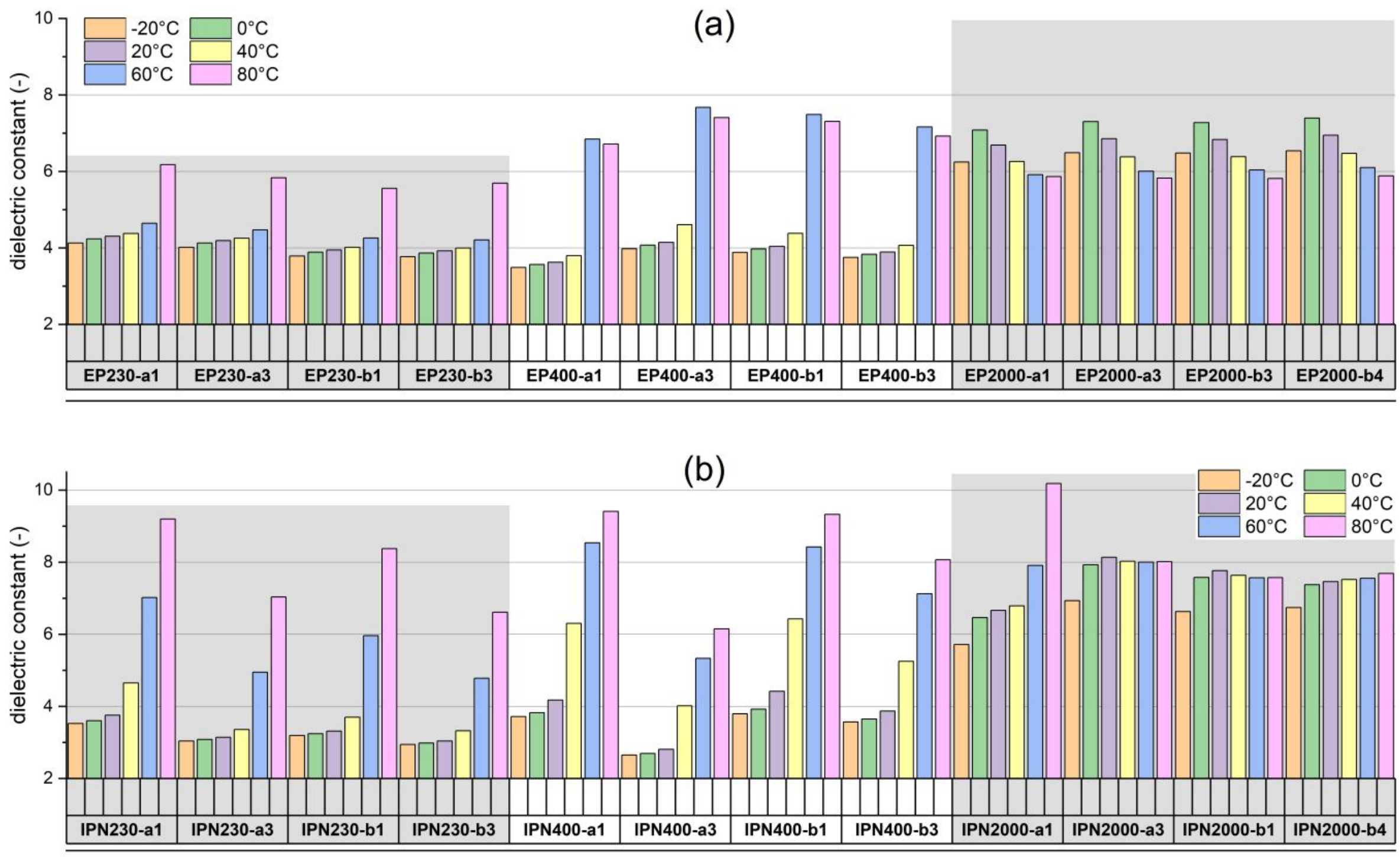

Fig. S 13. *Comparative diagrams of dielectric constant for (a) EP and (b) IPN at a frequency of 50 Hz (IPN variants IPN400-a2 and IPN400-b2 are not included).*

Table S 1. *Summary of dielectric constant and dissipation factor values for selected temperatures above 0 °C for all tested samples.*

materials code	Dielectric constant (-)				Dissipation factor (-)			
	20°C	40°C	60°C	80°C	20°C	40°C	60°C	80°C
EP230-a1	4.30	4.38	4.65	6.18	4.80E-03	3.60E-03	7.77E-03	8.19E-02
EP230-a3	4.19	4.26	4.47	5.83	5.09E-03	3.81E-03	8.74E-03	8.86E-02
EP230-b1	3.95	4.02	4.26	5.56	4.85E-03	3.68E-03	7.46E-03	6.61E-02
EP230-b3	3.93	4.00	4.21	5.69	4.68E-03	3.39E-03	6.42E-03	7.41E-02
EP400-a1	3.62	3.80	6.85	6.71	4.15E-03	6.71E-03	6.17E-02	2.28E-01
EP400-a3	4.15	4.61	7.68	7.41	5.00E-03	2.03E-02	6.50E-02	3.60E-01
EP400-b1	4.04	4.38	7.49	7.31	4.75E-03	1.34E-02	6.06E-02	1.48E-01
EP400-b3	3.89	4.07	7.16	6.92	4.24E-03	7.95E-03	5.92E-02	2.33E-01
EP2000-a1	6.69	6.26	5.92	5.86	2.77E-02	2.11E-01	9.45E-01	2.60E+00
EP2000-a3	6.85	6.38	6.00	5.82	3.74E-02	2.88E-01	1.26E+00	3.59E+00
EP2000-b3	6.83	6.39	6.04	5.81	3.61E-02	2.70E-01	1.18E+00	3.33E+00
EP2000-b4	6.95	6.47	6.10	5.89	3.51E-02	2.64E-01	1.16E+00	3.32E+00
DA230-a1	3.81	3.91	5.60	11.34	7.77E-03	8.32E-03	1.36E-01	1.34E+00
DA400-a1	3.88	4.28	6.51	9.46	1.04E-02	2.24E-02	1.44E-01	1.20E+00
DA2000-a1	6.67	7.28	7.96	8.09	4.77E-01	2.40E+00	8.71E+00	3.49E+01
IPN230-a1	4.26	4.48	6.64	9.36	1.23E-02	4.35E-02	9.91E-02	1.65E-01
IPN230-a3	3.14	3.36	4.95	7.04	5.85E-03	1.25E-02	5.40E-02	1.04E-01
IPN230-b1	3.31	3.70	5.96	8.38	5.97E-03	1.90E-02	7.69E-02	1.30E-01
IPN230-b3	3.04	3.32	4.78	6.61	4.90E-03	1.54E-02	5.36E-02	9.01E-02
IPN400-a1	4.17	6.30	8.54	9.41	2.42E-02	9.36E-02	7.97E-02	2.91E-01
IPN400-a2	4.03	5.51	8.14	9.58	1.26E-02	7.17E-02	1.18E-01	3.77E-01
IPN400-a3	2.81	4.02	5.33	6.15	1.07E-02	5.28E-02	6.90E-02	1.28E-01
IPN400-b1	4.42	6.43	8.43	9.33	3.37E-02	9.06E-02	8.54E-02	2.89E-01
IPN400-b2	4.60	6.80	8.65	9.27	3.85E-02	9.53E-02	9.21E-02	4.04E-01
IPN400-b3	3.87	5.25	7.13	8.07	1.64E-02	6.08E-02	8.34E-02	1.40E-01

IPN2000-a1	6.66	6.79	7.91	10.18	9.31E-02	4.47E-01	1.39E+00	4.69E+00
IPN2000-a2	8.14	8.03	8.00	8.02	4.41E-02	1.57E-01	6.20E-01	2.82E+00
IPN2000-b1	7.77	7.64	7.57	7.57	5.14E-02	2.07E-01	8.42E-01	3.62E+00
IPN2000-b4	7.46	7.52	7.56	7.69	1.45E-01	6.86E-01	2.35E+00	6.71E+00

4 Self-healing – Tensile parameters of intact and self-healed IPN2000

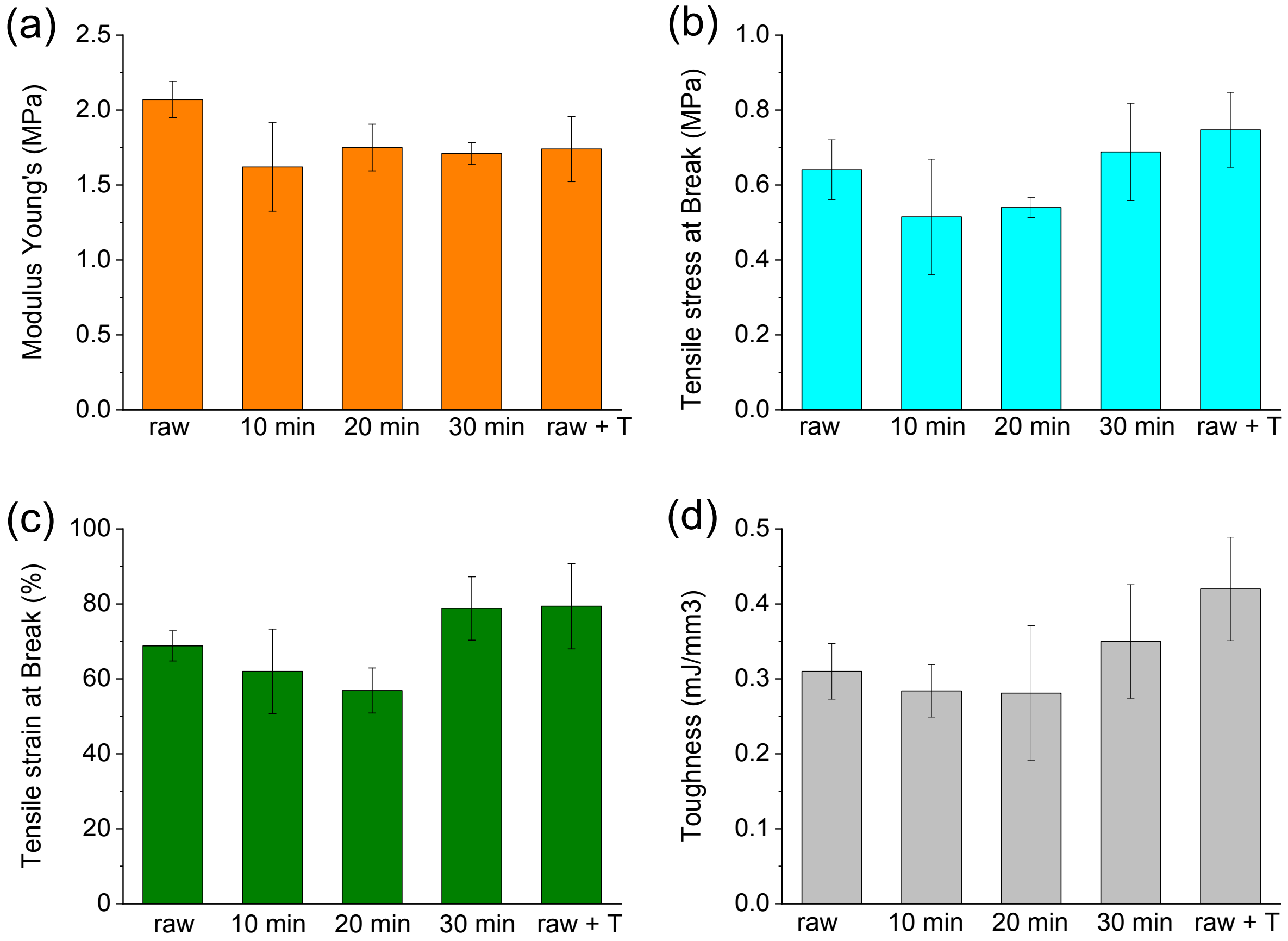

Fig. S 14. *Trends of individual Tensile properties of the IPN 2000 system, obtained for intact, or for disrupted and subsequently self-healed specimens after different healing times at 120°C; one mechanically intact specimen (label: "raw + T") also was subjected to annealing at 120°C for 30 min for comparative reasons; the different specimen types are aligned on the X-axis; Compared properties are (a) Young's moduli, (b) Stress at break (strength), (c) Strain at break (extensibility), (d) Toughness.*

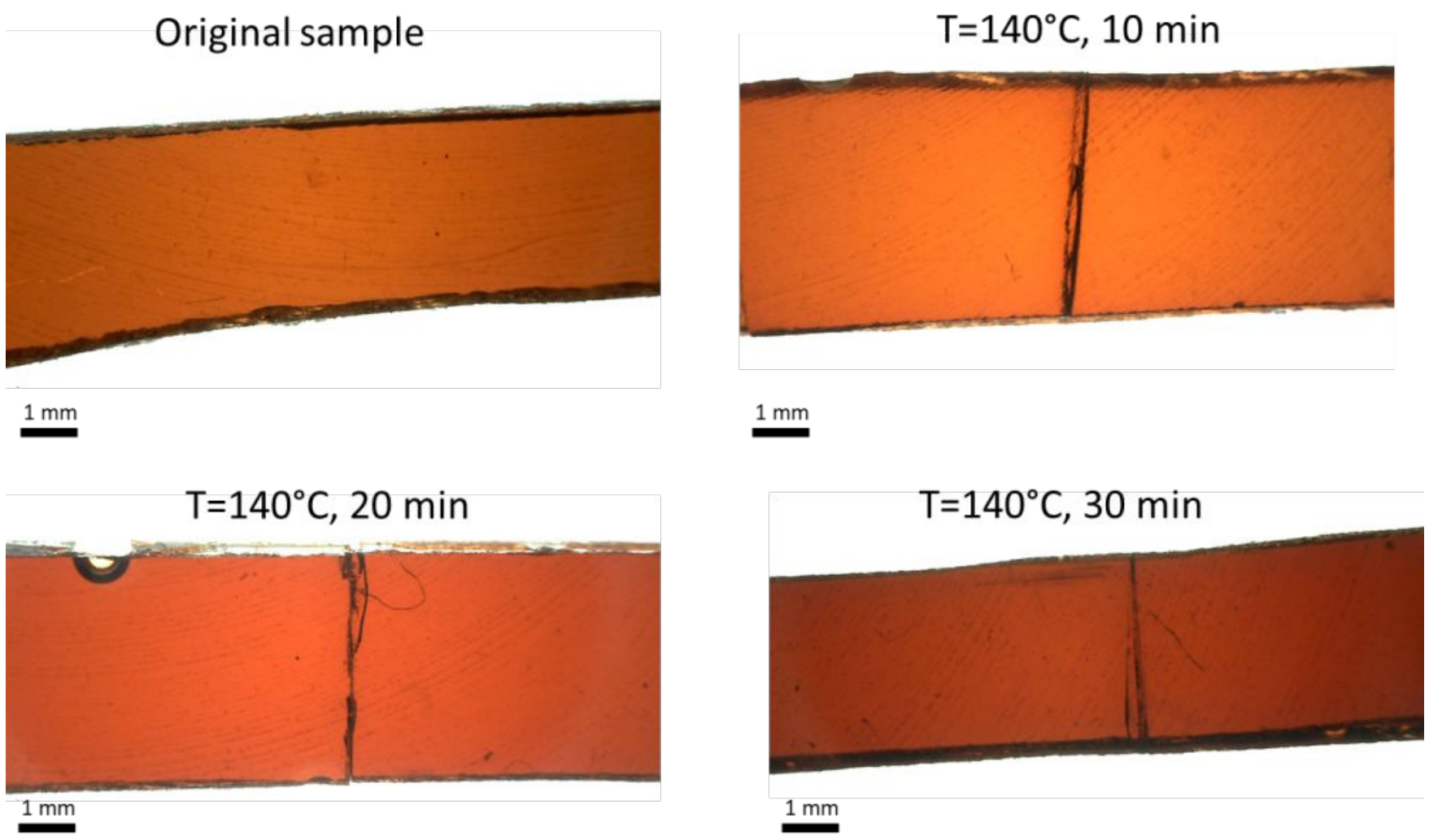

Fig. S 15. *Visual observation of the success of self-healing (at 140°C) of an IPN400 specimen, which was cut in two.*

5 List of Figures and Tables in the Main Manuscript

Fig. 1. *Structures of the chemical components of (a) the permanent network (DGEBA and Diamine), and (b) of the reversible Diels-Alder network (BMI and FD); synthesis of the tetra-functional diene component FD (tetra-furan) of the reversible network via modification of the amine component D of the permanent epoxy network (violet arrow); (c) process steps during the synthesis of specimens of the studied materials.*

Fig. 2. *Formation of (a) reversible and (b) permanent networks, as well as (c) their combination to an interpenetrating network; The desired (d, e) and the undesired (f, g) reactions during the resins cure: (d) The DA addition reaction of furan and maleimide units which is reversible (retro-DA) at higher temperatures, and which leads to the formation of the reversible network; (e) the irreversible epoxy−amine curing reaction which yields the permanent network; Side reactions: (f) Michael addition of amine and maleimide, and (g) radical polymerization of maleinmide units.*

Fig. 3. *Three possible routes of curing the interpenetrating networks: (1) low-temperature cure – the reversible DA network (red) rapidly polymerizes, and the permanent EP network (green) grows slowly inside the cured DA; (2) simultaneous growth of both networks – difficult to achieve and temperature would need to be adjusted all the time; (3) high-temperature cure – the reversible DA network stays dissociated while the EP network rapidly grows, until in the final step the temperature is lowered and the DA network grows inside the EP one.*

Fig. 4. *DMTA profiles of: (a, b) pure permanent EP networks EP230, EP400, and EP2000; (c, d) pure reversible DA network DA400; (e, f) interpenetrating networks IPN230, IPN400, and IPN2000; temperature dependencies of (a, c, e) storage modulus G' and (b, d, f) loss factor* $\tan\delta_m$. *The temperature scale of the DMTA profiles of DA400 in the graphs (c, d) is narrower than in case of EP and IPN – hence the vertical thin lines in the graphs (a, b, e, f) indicate the depicted temperature window of DA.*

Fig. 5. *Effect of Curing Mode on the DMTA profile of IPN400; EP400-b3 and DA400-a1 are shown as reference materials; Inlay photographs: samples IPN400-a2 and IPN400-b2 after disruption (by cutting) and subsequent self-healing at 140°C for 30 min.*

Fig. 6. *Reversible crosslinking analysed by DMTA and relaxation tests: (a) Cyclic heating/cooling tests with the sample IPN400-b2, between 30 and 150°C; depicted are the temperature-dependent curves of the storage modulus G' (a: top) and of the loss factor* $\tan\delta_m$ *(a: bottom); the yellow arrow in (a) highlights the region of reversible crosslinking; (b) Stress relaxation tests at different temperatures: time-dependent relaxation modulus.*

Fig. 7. *DSC curves: (a) thermograms of the pure reversible network DA400-a1 (top: light/dark green), of the pure permanent network EP400-b3 (center: light/dark blue), and of the related interpenetrating network IPN400-b2 (bottom: light/dark red); the heating scans were performed up to 250°C; the respective lighter lines represent the* 1st *heating scans, the darker ones the respective* 2nd *heating scans; (b, c) Results of the repeated heating of the most promising interpenetrating network IPN400-b2: (b) up to T = 150°C and (c) up to T = 120°C; cooling traces are omitted for clarity in (a, b, c).*

Fig. 8. *Frequency-temperature dependencies of dissipation factor for (a) EP, (b) DA, (c) IPN at the shortest curing mode a1, and (d) IPN at the most extended curing mode b3 or b4.*

Fig. 9. *Comparative diagrams of dissipation factors for (a) EP and (b) IPN at a frequency of 50 Hz (IPN variants IPN400-a2 and IPN400-b2 are not included).*

Fig. 10. *Self-healing: (a) Tensile curves of intact and of disrupted and subsequently self-healed samples of the system IPN 2000-b4 after different healing times at 120°C, compared with intact reference sample (black line), and with intact reference annealed for 30 min at 120°C (black dotted line); the tensile curves were recorded at room temperature, where IPN 2000 is rubbery; (b) Tensile curves of IPN400: intact specimen (black line), and re-connected self-healed samples of IPN400 after healing at 140°C for 10, 20, and 30 min (red, green, and blue line, respectively); the tensile curves were recorded at 90°C, where IPN 400 is rubbery; (c) Visual observation of the success of self-healing (at 120°C) of a specimen of IPN2000, which was cut in two.*

Fig. 11. *(a, b) Healing (at 140°C for 30 min) of microcracks caused by bending and (c, d) damage caused by dielectric breakdown at 25 kV, as observed by light microscopy: (a, c) the IPN400-b2 system; the orange (up to dark red) color is characteristic of the studied IPN double networks; (b, d) the EP400 system.*

Fig. 12. *Graphical overview of the electrical treeing and of subsequent thermal self-healing experiments performed on IPN2000 and on the EP2000 reference system: a) optical micrograph of an electrical tree generated in an IPN2000 specimen; b) the same specimen imaged from the identical viewing direction after thermal self-healing; in both images, the needle electrode remained embedded in the specimen. c) Optical micrograph of an EP2000 reference specimen after electrical treeing; d) the same EP2000 specimen after exposure to the identical thermal treatment like the one applied to IPN2000; in case of the EP2000 specimens, the needle electrode was removed prior to imaging, just after terminating the tree growth; e) schematic illustration of the experimental electrical treeing setup.*

Scheme 1. *Schematic setup of the dielectric breakup experiments (left), the real installation immersed in oil (center), and exemplary specimens shown after enduring dielectric breakup damage.*

Table 1. *List of all the tested materials, including a description of curing modes.*
